# Catalytic transformation of functionalized carboxylic acids using multifunctional rhenium complexes

**DOI:** 10.1038/s41598-017-03436-y

**Published:** 2017-06-13

**Authors:** Masayuki Naruto, Santosh Agrawal, Katsuaki Toda, Susumu Saito

**Affiliations:** Graduate School of Science, Nagoya University, Chikusa, Nagoya 464-8602 Japan

## Abstract

Carboxylic acids (CAs) are one of the most ubiquitous and important chemical feedstocks available from biorenewable resources, CO_2_, and the petrochemical industry. Unfortunately, chemoselective catalytic transformations of CH_*n*_CO_2_H (*n* = 1–3) groups into other functionalities remain a significant challenge. Herein, we report rhenium^V^ complexes as extremely effective precatalysts for this purpose. Compared to previously reported heterogeneous and homogeneous catalysts derived from high- or low-valent metals, the present method involves a α-C–H bond functionalization, a hydrogenation, and a hydrogenolysis, which affords functionalized alcohols with a wide substrate scope and high chemoselectivity under relatively mild reaction conditions. The results represent an important step toward a paradigm shift from ‘low-valent’ to ‘high-valent’ metal complexes by exploring a new portfolio of selective functional group transformations of highly oxygenated organic substrates, as well as toward the exploitation of CAs as a valuable biorenewable feedstock.

## Introduction

Carboxylic acids (CAs) are abundantly available from a variety of natural resources and the petrochemical industry, while several emerging technologies aim to produce CAs via thermal^1–4^ or photo-induced^5–7^ immobilization of CO_2_ in organic frameworks. The functional group transformation (FGT) and subsequent reduction of CAs hence represent an important research subject to furnish alcohols for platform/fine chemicals^1–11^, biofuels^11–13^, and electric power-storage materials^14^. Among those FGTs, the catalytic C–H bond functionalization of CAs^15–17^, followed by hydrogenation^18–23^, could potentially widen the diversity of alcohols thus available. Unfortunately, this route remains unattainable, given that a general method for the selective catalytic activation of different functional groups (FGs) in CAs presents many challenges. Generally, CAs contain thermodynamically stable and kinetically inert α-C–H hydrogen atoms (carboxylates: p*K*_a_ = 34–40 in DMSO), which are less acidic than those of the CO_2_H group (p*K*_a_ = 10–13 in DMSO), while the carbonyl carbon atoms of CAs are less electrophilic than those of other carbonyl groups. The development of new catalytic strategies to chemoselectively activate the CH_*n*_CO_2_H (*n* = 1–3) groups of aliphatic CAs should expand the synthetic utility of functionalized CAs as important platform/fine chemicals.

**Figure 1:**
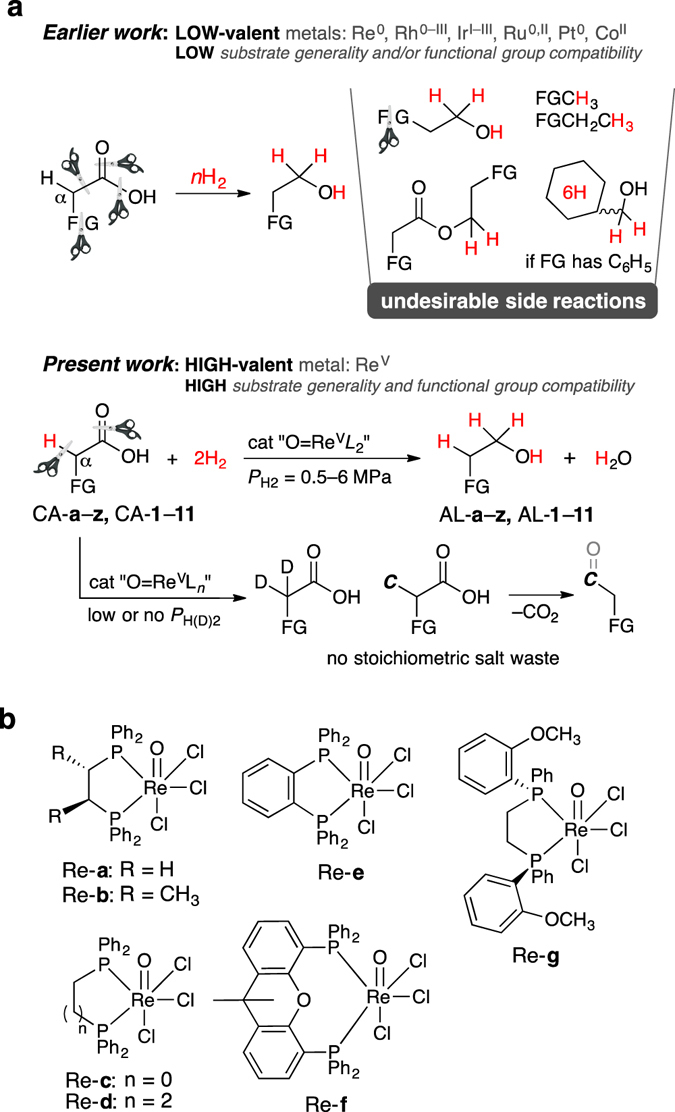
Overview of the present work (**a**). Overcoming the drawbacks of previously reported catalytic systems for the chemoselective hydrogenation of CAs. FG = functional group, L = ligand, ***C*** = carbon fragment. (**b)** Some Re^V^ = O complexes tested thus far for CA hydrogenation.

Previously reported attempts to selectively transform CAs are predominantly based on specifically developed molecular catalysts. For example, the selective α-C–H bond functionalization of CAs is accomplished by catalytic BH_3_ in the presence of stoichiometric amounts of amines^15^. Moreover, CO_2_H moieties have been used as directing groups for the Pd-catalyzed C–H bond functionalization of spatially distal sp^2^ or sp^3^ carbon atoms^16, 17^, and as traceless activating groups for decarboxylative carbon–carbon bond formations^24–27^. The latter two methods are based on redox catalysis, involving low-valent (d_3_–d_10_) metals such as Ni^0–III^, Pd^0,II^, Ru^0,II,IV^, Ir^I,III^, or Rh^I,III^. Despite the numerous beneficial features of low-valent metal species, the catalytic FGT of CAs (including hydrogenation) frequently suffers from several drawbacks, i.e., side reactions including the oxidative addition of the substrate at high temperatures, decarboxylation, and over-reduction (Fig. 1a)^23^, as well as catalyst deactivation by ligation/chelation of one or more heteroatom-containing FGs to the metal center. For example, in hydrodesulfurization reactions in biorefinery and mature oil refinery processes, hydrogenation catalysts are frequently deactivated by thiophenes^28, 29^. Recently developed molecular Ru^II^ complexes^18^ are able to catalytically hydrogenate CAs, but are not compatible with several FGs (e.g. bromoaryls, thienyls, and amides). To avoid such catalyst deactivation, the robustness and inertness of the catalyst toward many different FGs is of crucial importance, while new concepts for the selective activation of CH_*n*_CO_2_H groups (*n* = 1–3) in CAs must be developed. High-valent (d_0_–d_2_)^30^ transition metals may represent more promising prospectives than their low-valent analogues, considering that the former are less susceptible to oxidative addition and π-back donation than the latter. Therefore, we have developed molecular single-active-site rhenium^V^ complexes (Fig. 1b) that selectively hydrogenate and/or functionalize a wide range of functionalized CAs. The majority of high-valent Re complexes have thus far been used for oxidation^31^ and deoxydehydration^31, 32^, rather than for FGT of CA (α-C–H functionalization^15^ and hydrogenation^23, 33^).

## Results

### Catalytic hydrogenation of functionalized CAs

We recently reported a systematic study on the nature of cationic mononuclear Ru^II^ carboxylates as catalyst precursors for the self-induced hydrogenation of CAs^18^. Simultaneously, a similar hydrogenation mechanism involving cobalt(II)-triphos catalysts (triphos: CH_3_C[CH_2_PPh_2_]_3_) was proposed by Elsevier and Bruin^19^. Subsequently, we extended the putative hydrogenation mechanism to high-valent metal catalysts^34^, as the originally proposed mechanism seemed to involve acid–base cooperative catalysis rather than a redox-based catalytic cycle.

Initially, we evaluated a series of commercially available high-valent Re catalyst precursors. Treatment of a toluene solution of 3-phenylpropanoic acid (CA-**a**) with catalytic amounts of (CH_3_)Re^VII^O_3_, IRe^V^O_2_(PPh_3_)_2_, Cl_3_Re^V^O(O = PPh_3_)[(CH_3_)_2_S], Cl_3_Re^V^O(PPh_3_)_2_, or Cl_3_Re^V^O(Ph_2_PCH_2_PPh_2_) (Re-**c**, Fig. 1b; Ph = C_6_H_5_) under an H_2_ atmosphere (*P*_H2_ = 8 MPa) at 160 °C for 24 h induced virtually no reaction (*cf*. Table 1, entry 1 and Supplementary Table 1). In contrast, the cationic Re species from each of the latter four complexes (2 mol% each), formed upon treatment with Na[B(C_6_H_5_)_4_] (10 mol%) ([Re]_0_ = 2.5 mM), afforded 3-phenylpropan-1-ol (AL-**a**) in 14–62% and 3-phenylpropyl 3-phenylpropanoate (ES-**a**) in 5–11% (entry 2 and Supplementary Table 1). The highest yield of AL-**a** (72%) was obtained using Re-**c** (entry 3). Encouraged by these results, we used several Re^V^ precatalysts (Re-**a**,**d**–**f**) obtained from Cl_3_Re^V^O(O = PPh_3_)[(CH_3_)_2_S]^35^ and bidentate diphosphine ligands (entries 4–7). The best results were observed for Cl_3_Re^V^O[Ph_2_P(CH_2_)_2_PPh_2_]^35^ (Re-**a**), which furnished AL-**a** in >98% yield (entry 4) together with negligible amounts of ES-**a** (~1%). This result stands in sharp contrast to many homogeneous and heterogeneous catalysts, which induce undesirable CA esterification and deoxygenation with the generated alcohols^36–38^. The exclusive formation of AL-**a** suggests that undesirable over-reductions (e.g. dearomatic hydrogenation and hydrogenolysis) of AL-**a**, and/or decarboxylation of CA-**a**, are prevented. This high FG tolerance surpasses that of Mo(CO)_6_-Rh/Al_2_O_3_ (*P*_H2_ = ~10 MPa, 150 °C), in which benzene rings of the CAs are hydrogenated^39^. Moreover, the low-valent Re^0^ carbonyl cluster Re_2_(CO)_10_ is unable to catalyze the hydrogenation of *n*-C_14_H_29_CO_2_H in dimethoxyethane at 170 °C, even at *P*_H2_ = ~10 MPa^39^. Similarly, non-oxo complexes of Re^III^ exhibited low to negligible catalytic activity (entries 8 and 9).

**Table 1:** Re complexes for the catalytic hydrogenation of CA-**a**.


Entry	Re complex	Na[BPh_4_]	Yield % of AL-**a**	Yield % of ES-**a**
1	Cl_3_Re^V^O[P(C_6_H_5_)_3_]_2_	—	~1	~1
2	Cl_3_Re^V^O[P(C_6_H_5_)_3_]_2_	+	62	9
3	Re-**c**	+	72	5
4	Re-**a**	+	>98	~1
5	Re-**d**	+	46	6
6	Re-**e**	+	89	5
7	Re-**f**	+	34	5
8	Cl_3_Re^III^[CH_3_C(CH_2_P(C_6_H_5_)_2_)_3_]	+	9	4
9	Cl_3_Re^III^[P(C_6_H_5_)_3_]_2_(CH_3_CN)	+	37	5

Unless otherwise specified: CA-**a**:Re:Na[B(C_6_H_5_)_4_] = 100:2:10 (mol%); [Re]_0_ = 2.5 mM; *P*_H2_ = 8 MPa, 160 °C, 24 h. Ph = C_6_H_5_. Yields % are determined by ^1^H NMR using the internal standard anisole.

Although both low- and high-valent Re species in hetero-multimetallic catalysts such as Re_2_(CO)_10_-Ru_3_(CO)_12_^39^, Re_2_(CO)_10_-Rh/Al_2_O_3_^39^, Re_2_O_7_-OsO_4_^40^, ReO_*x*_-Pd/SiO_2_^41^, ReO_*x*_/TiO_2_^42^, and Re/TiO_2_^43^ have been investigated for CA hydrogenation^23, 33^, the specific role of Re in these catalysts remains unclear. Recent examples of ReO_*x*_/TiO_2_ and Re/TiO_2_ catalysis have shown high alcohol selectivity with a rather limited substrate scope, with diverse Re entities (Re^0^, Re^III^, Re^IV^, Re^VI^, and Re^VII^, calcined and reduced at 400–500 °C with H_2_) on TiO_2_ determined by XPS analysis^42, 43^. Even though heterogeneous ReO_3_ promotes CA hydrogenation under high H_2_ pressure (*P*_H2_ = ca. 20.5 MPa, ~165 °C), the *in situ* esterification is non-negligible and benzoic acid is partially hydrogenated^44^.

**Figure 2:**
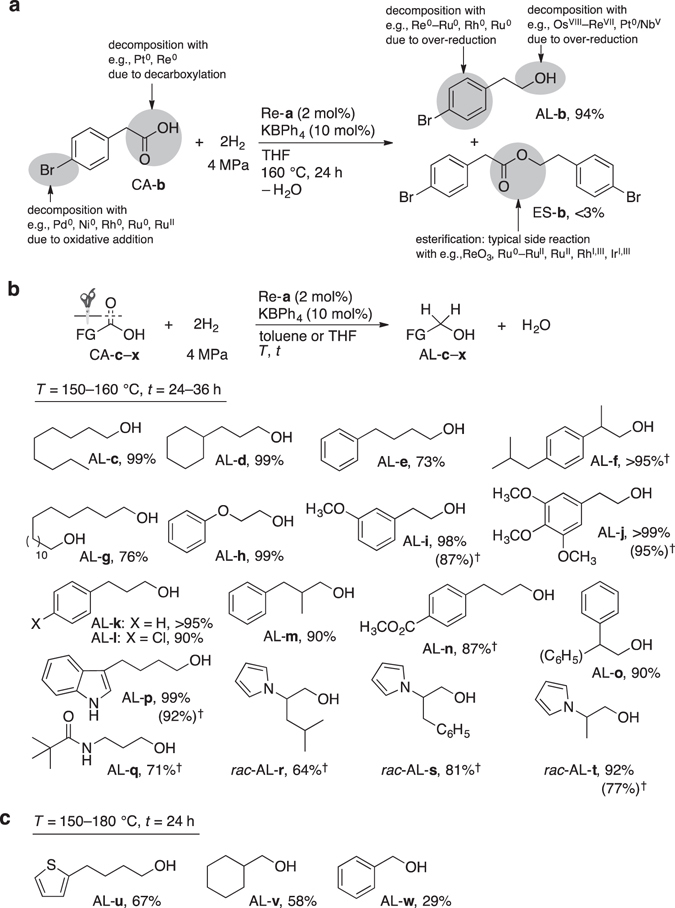
Catalytic hydrogenation of various CAs using Re-a. See Supplementary Tables 1–5 for experimental details. (**a**) In contrast to the low selectivity potentially obtainable with previously reported methods, hydrogenation of CA-**b** with Re-**a** afforded AL-**b** selectively. (**b**) CAs hydrogenated in relatively high yields. CA-**g** is a monocarboxylic acid, while CA-**l**–**n** are 2-enyl-1-CAs (for the exact structures, see main text. ^†^Isolated yield. (**c**) CAs hydrogenated in lower yields.

Using K[B(C_6_H_5_)_4_] instead of Na[B(C_6_H_5_)_4_] with Re-**a** under milder conditions (*P*_H2_ = 4 MPa, 150 °C, 24 h) increased the yield of AL-**a** from 55% to 80% (Supplementary Table 2). After further optimization of the reaction conditions (Supplementary Table 3) ([Re-**a**]_0_ = 2.5 mM in toluene or THF, Re-**a**:K[B(C_6_H_5_)_4_] = 0.02:0.1, *P*_H2_ = 2–4 MPa, 140–160 °C), a variety of CAs were tested (Fig. 2a–c and Supplementary Table 4). The hydrogenation of brominated CA-**b** with Re-**a** afforded AL-**b** with an intact bromobenzene fragment (Fig. 2a). Low-valent metal species such as previously developed Ru^II^ complexes^18^ lose their catalytic activity almost immediately upon reaction with CA-**b**, affording only negligible amounts of AL-**b** (<1%), presumably due to the oxidative addition of the C–Br bond to the Ru center. The new hydrogenation reaction is applicable to a wide range of functionalized and simple CAs (Fig. 2b). The hydrogenation of CA-**a** with Re-**a** proceeded under even milder conditions (*P*_H2_ = 2 MPa, 160 °C, 72 h), affording AL-**a** in 85% yield. Simple aliphatic CAs (CA-**c**–**f**), 17-hydroxyheptadecanoic acid (CA-**g**), and alkoxy-substituted CAs (CA-**h**–**j**) were also hydrogenated under these conditions. The double bonds of the α,β-unsaturated CAs (*E*)-3-(4-chlorophenyl)acrylic acid (CA-**l**), (*E*)-2-methyl-3-phenylacrylic acid (CA-**m**), and (*E*)-3-(4-(methoxycarbonyl)phenyl)acrylic acid (CA-**n**) were hydrogenated uniformly, affording saturated alcohols AL-**l**–**n**. Nevertheless, numerous FGs, including chlorobenzenes, ethers, alcohols, esters (CA-**n**), indole (CA-**p**), amide (CA-**q**), thiophene (CA-**u**), and pyrroles (CA-**r**–**t**) were tolerated well and barely inhibited the hydrogenation. *N*-Protected natural α-amino acids (CA-**r**–**t**) were hydrogenated under racemization of the stereogenic carbon centers. Alcohols AL-**b**–**t** were produced uniformly and almost exclusively, while esterification was consistently negligible (<5%). A control experiment revealed that CAs are hydrogenated faster than the esters under the applied conditions. For example, hydrogenation of methyl nonanoate with a mixture of Re-**a** (2 mol%) and K[B(C_6_H_5_)_4_] (10 mol%) afforded 1-nonanol in ~10% yield under harsh conditions (*P*_H2_ = 4 MPa, 180 °C, 24 h; [Re]_0_ = 2.5 mM) (Supplementary Table 5), which suggests that ester and amide groups are tolerated under mild hydrogenation conditions. In contrast, LiAlH_4_ or LiBH_4_ reduce the CA moieties and bromoaryl, ester, or amide functionalities.

**Figure 3:**
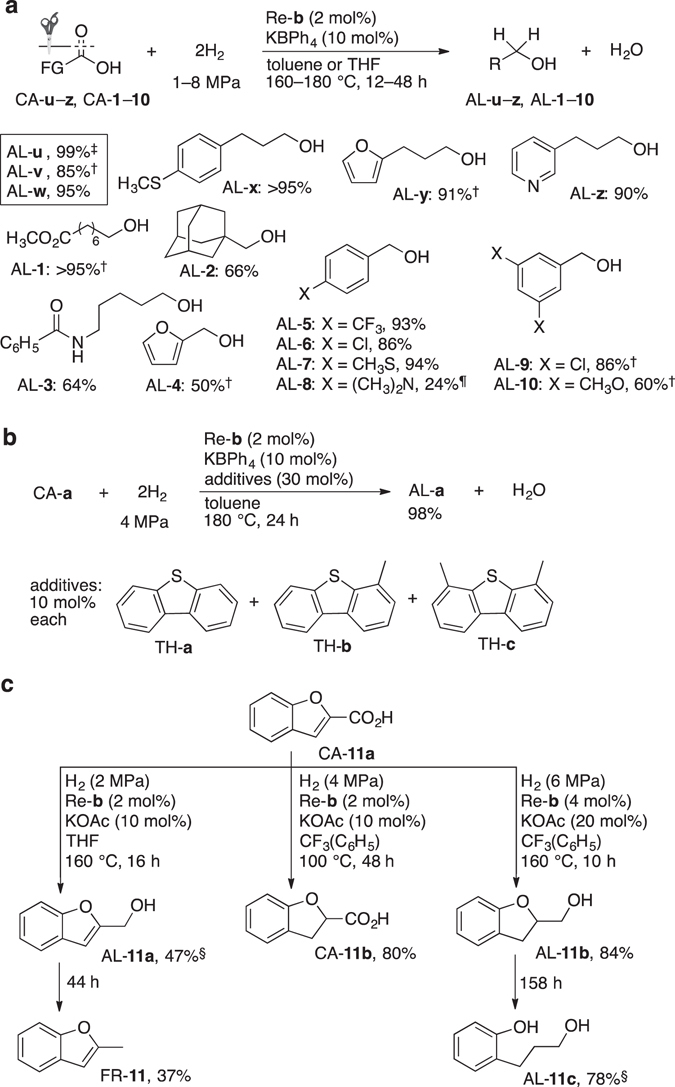
Improved chemoselective hydrogenation of CAs and hydrogenolysis of the resulting α-alkoxy alcohols using Re-b. See Supplementary Tables 6–10 for experimental details. (**a**) Substrate scope for the catalytic hydrogenation of CAs using Re-**b**. Unless otherwise noted, the corresponding esters were obtained in ≤5%. ^†^THF was used instead of toluene. ^‡^Isolated yield: 83%. ^¶^Ester ES-**8** (~11%) was also obtained. (**b**) Re^V^-catalyzed CA hydrogenation in the presence of typical sulfur-containing substances generated by hydrodesulfurization during the refinement process of mature oil. **c**. Selective hydrogenation of CA-**11a** and further hydrogenolysis. Yields (%) are based on CA-**11a**. ^§^AL-**11b** (16–20%) was also obtained.

While aliphatic CAs generally represent excellent substrates for the present hydrogenation strategy, the hydrogenation of benzoic acid (CA-**w**) has rarely been examined^18–21, 43^. Indeed, hydrogenation of CA-**w** with Re-**a** proceeded sluggishly even under slightly harsher conditions (*P*_H2_ = 4 MPa, 180 °C, 24 h), affording benzyl alcohol (AL-**w**) in 29% yield (Fig. 2c). To improve this result, a new Re^V^OCl_3_ complex (Re-**b**) with a more robust five-membered Re–ligand metallacycle (due to the Thorpe-Ingold effect) was synthesized. The hydrogenation of CA-**w** with Re-**b** (2 mol%) and K[B(C_6_H_5_)_4_] (10 mol%) proceeded more effectively (*P*_H2_ = 4 MPa, 160 °C, 40 h) with full conversion of CA-**w** to furnish AL-**w** in high yield (95%) (Fig. 3a). For comparison, AL-**w** is generated in 93% (95% selectivity) and 62% yield under harsher conditions using a Ru-triphos (2 mol%, *P*_H2_ = ca. 5 MPa, 220 °C, 24 h)^21^ and Co-triphos complex (2.5 mol%, *P*_H2_ = ca. 8 MPa, 100 °C, 22 h)^19^, respectively.

The hydrogenation of aliphatic CAs was reinvestigated using Re-**b** (2 mol%), furnishing a higher yield of AL-**a** (93%) under milder conditions (*P*_H2_ = 2 MPa, 160 °C, 24 h) compared to Re-**a** (35%) and Re-**g** (32%) (Supplementary Table 1). Even at lower *P*_H2_ (*P*_H2_ = 0.5 MPa, 180 °C, 48 h), CA-**a** was hydrogenated effectively with Re-**b** to afford AL-**a** (84%) and ES-**a** (7%) (Supplementary Tables 6 and 7). The furan (FR) and pyridine moieties of CA-**y**,**z**, and CA-**4** were well tolerated. Other CAs such as benzoic acid derivatives (CA-**x**–**z** and CA-**5**–**10**) were poorly hydrogenated by Re-**a**, but underwent smooth hydrogenation with Re-**b**, affording the corresponding alcohols in high yield and selectivity (Supplementary Table 8).

Molecular Re^VII^ and Re^V^ species are able to catalyze the hydrogenation of sulfoxides, which affords dialkyl sulfides as the major product^33^. Surprisingly, the hydrogenation of CA with Re-**b** was barely inhibited by sulfur-containing CAs or thiophene (TH) derivatives such as CA-**u**,**x** and CA-**7** (Supplementary Table 8). For example, 4-(thien-2-yl)-substituted CA-**u** was hydrogenated with Re-**b** to give AL-**u** in 99% yield; hydrogenation of CA-**x** proceeded almost quantitatively even at milder *P*_H2_ (*P*_H2_ = 1 MPa, 180 °C, 12 h). In many cases, sulfur-containing substances poison precious metal hydrogenation catalysts. However, Re-**b** was not deactivated by benzothiophene or dibutylsufide (30 mol%), affording AL-**a** in 99% in both cases (*P*_H2_ = 4 MPa, 160 °C, 24 h). Even in the presence of a mixture of dibenzothiophene derivatives (TH**-a–c**), which are detrimental to conventional hydro-desulfurization catalysts^28, 29^, no negative effects were observed at *P*_H2_ = 4 MPa (Fig. 3b; Supplementary Table 9). In contrast, TH slightly decreases the hydrogenation rate of the bimetallic catalyst OsO_4_-Re_2_O_7_, although over-reduction of the hydrocarbon was diminished^40^.

Compared to the selective formation of AL-**4** from furan-2-carboxylic acid (CA-**4**), the π-extended derivative CA-**11a** showed intriguing reactivity upon reaction with Re-**b** and H_2_ (Fig. 3c; Supplementary Table 10). CA-**11a** underwent either full hydrogenation of all non-aryl unsaturated bonds to afford AL-**11b**, or chemoselective reduction, i.e., a carbonyl hydrogenation to afford AL-**11a** or an α,β-ene hydrogenation to furnish CA-**11b**. Even hydrodeoxygenation (HDO), which is uncommon for Re complex catalysts, was achieved by varying the reaction parameters: hydrogenolysis of the different C–O bonds of the reaction intermediates AL-**11a** and AL-**11b** afforded FR-**11** and alcoholic phenol AL-**11c**, respectively. To the best of our knowledge, this represents the first example of a directed, catalytic CA hydrogenation and subsequent hydrogenolysis in one pot using molecular catalysts.

### Catalytic α-C–H bond functionalization of CAs

**Figure 4:**
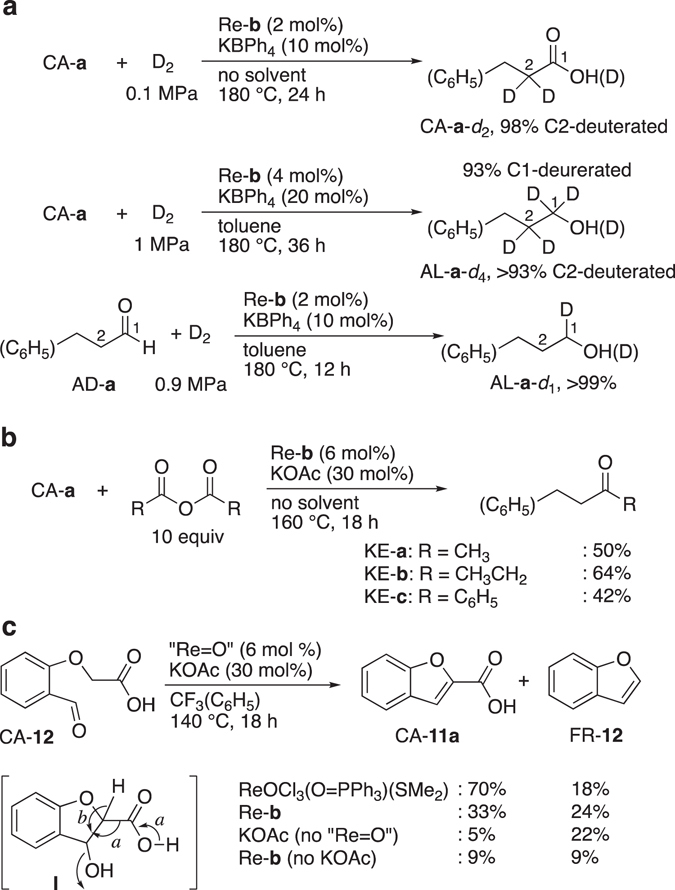
α-C–H functionalization of CAs. (**a**) C1- and/or C2-deuteration. (**b**) Intermolecular C–C bond formation in acid anhydrides to afford ketones. (**c**) Intramolecular aldol condensation. *a*: Decarboxylative dehydration; *b*: Second deprotonation followed by dehydration.

So far, Re-**a** and Re-**b** have provided the best catalytic results for the hydrogenation of a broad variety of CAs under relatively mild conditions. However, as previously discussed, Re-**a** induces the epimerization of *N*-protected amino acids such as CA-**r**–**t** (*P*_H2_ = 4 MPa, 160 °C). This result suggests that a catalyst derived from Re-**a** easily deprotonates the α-C–H moiety of these CAs, while the racemization may occur before or during the hydrogenation. Indeed, decreasing *P*_H2_ (1 → 0.1 MPa) under otherwise identical conditions resulted in the apparent full recovery of CA-**a**, i.e., a recovery most likely due to a very fast α-C–H deprotonation–protonation sequence. Likewise, replacing H_2_ with D_2_ [Re-**b** (2 mol%), K[B(C_6_H_5_)_4_] (10 mol%), *P*_D2_ = 0.1 MPa, 180 °C] did not introduce deuterium on the carbonyl carbons; instead, in the absence of a solvent, the α-CH_2_ group of CA-**a** underwent fast H/D exchange to generate CA-**a**-*d*_*n*_ (*n* = 1, 2), whereby 98% of the α-CH_2_ moieties of CA-**a** were deuterated (Fig. 4a, top). Using 0.4 mol% of Re-**b** and 2 mol% of K[B(C_6_H_5_)_4_] afforded a deuteration of 80%, while Re-**b** (2 mol%), K[B(C_6_H_5_)_4_] (10 mol%), or KOAc (10 mol%) on their own resulted in negligible deuteration (≤5%) of the C2 position of CA-**a** under otherwise identical conditions (Supplementary Table 11). Increasing the catalyst loading and D_2_ pressure (*P*_D2_ = 1 MPa, 180 °C, 36 h, [Re-**b**]_0_ = 5.0 mM in toluene) furnished AL-**a**-*d*_*n*_ (*n* = 3 and 4) in 99% yield (deuteration: C1 = 93%, C2 >93%) (Fig. 4a, middle). In comparison, a combination of Ru^II^_2_Cl_2_(μ-Cl)_2_(μ-OH_2_)(Ph_2_P(CH_2_)_4_PPh_2_)_2_ (2 mol%) and Na(acetylacetonate) (10 mol%), which is an effective catalyst system for CA hydrogenation (*P*_H2_ = 2–6 MPa, 160 °C)^18^, resulted in 0% deuteration of CA-**a** under milder conditions (*P*_D2_ = 0.1 MPa, 180 °C). The hydrogenation of aldehyde AD-**a** (*P*_D2_ = 0.9 MPa) afforded AL-**a**-*d*_1_ in quantitative yield, while C2 was not deuterated (Fig. 4a, bottom). Even with the much higher acidity of the α-C–H of aldehydes (p*K*_a_ = ~16 in H_2_O) than that of carboxylates (p*K*_a_ 34–40 in DMSO), enolization of the aldehyde was prevented. Methyl 3-phenylpropionate underwent neither C2 deuteration nor hydrogenation (α-C–H of esters: p*K*_a_ = ~30 in DMSO), and full recovery of the ester was achieved under similar reaction conditions (*P*_D2_ = 0.9 MPa, 180 °C, 12 h).

Treating CA-**a** with acid anhydrides (AAs) in the presence of catalytic Re-**b** and KOAc, and in the absence of H_2_ and a solvent, afforded unsymmetrical ketones (KEs) KE-**a**–**c** in acceptable yields (Fig. 4b; Supplementary Table 12). In the absence of Re-**b** under otherwise identical conditions, KE-**a** was obtained in substoichiometric amounts (24%) relative to KOAc (30 mol%). This result highlights an important advantage of the method presented herein over previously reported strategies to synthesize methyl or phenyl ketones based on MeLi or PhLi, followed by aqueous workup^45^, or on ArB(OH)_2_/AAs and Pd catalysts^26^, which produce stoichiometric amounts of salts. The Dakin–West conditions (stoichiometric base in AA)^46^ are useful mainly for the synthesis of methyl ketones from amino acids and α-arylacetic acids, but catalytic base only promote the catalytic formation of KE-**a** from CA-**a** ^47^. In contrast, the present method opens a new route to the catalytic synthesis of KEs from two different CAs without generating any salt waste, albeit that one of the CAs must be converted to the AAs via dehydration before the reaction. Most likely, α-C–H deprotonation of CA-**a** and subsequent addition of the resulting enolate to the AA generates an intermediate β-ketoacid, which could undergo rapid decarboxylation to afford KE-**a**–**c**.

The deprotonation of the α-C–H moiety of CA-**a** with Re-**b** proceeds hence even in the absence of H_2_. However, decarboxylation frequently occurs before the deprotonative functionalization of the α-C–H groups of CAs when using low-valent transition metal catalysts^24–27^ or slightly basic reagents in buffered solutions^48^. For example, the intramolecular aldol cyclization of CA-**12** in AcOH using an excess of NaOAc (4.4 equiv relative to CA-**12**) exclusively promoted the decarboxylative dehydration (path *a*, Fig. 4c)^48^. The latter involves β-hydroxycarboxylic acid intermediate **I**, which generates FR-**12** upon elimination of the CO_2_H group. In contrast, Re complexes induce a double α-C–H deprotonation via the following fast reaction sequence: 1) α-C–H deprotonation of CA-**12**, 2) enolate addition, and 3) α-C–H deprotonation of **I** (path *b*). Using multifunctional Re^V^ complexes, the formation of CA-**11a** prevails over that of FR-**12** (Fig. 4c). When catalytic Re-**b** or Cl_3_Re^V^O(O = PPh_3_)[(CH_3_)_2_S] was used individually with a catalytic amount of KOAc, CA-**11a/**FR-**12** were obtained in 33%/24% and 70%/18% yield, respectively. In this particular case, CA-**11a** was obtained in higher selectivity in the absence of a bidentate phosphine. Cl_3_Re^V^O(O = PPh_3_)[(CH_3_)_2_S] alone afforded CA-**11a/**FR-**12** in 2%/4% yield (Supplementary Table 13).

## Discussion

**Figure 5:**
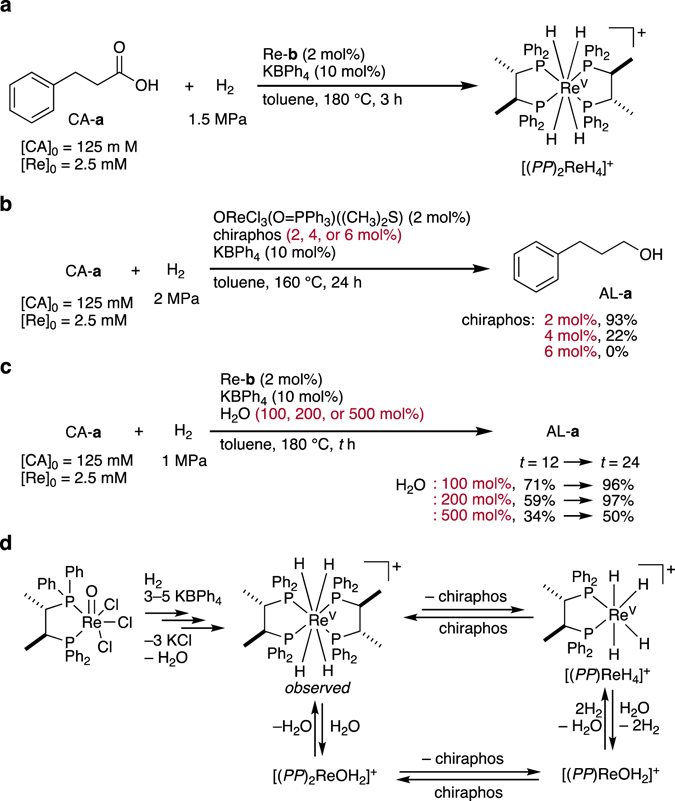
Control experiments to elucidate the structural change of Re-b in the presence of CA-a, H_2_, and K[BPh]_4_. (**a**) Predominant complex detected by ESI-MS: Re/(chiraphos)_2_. (**b**) Effect of the quantity of chiraphos on the reaction rate. (**c**) Effect of the quantity of water on the reaction rate. (**d**) Proposed structural changes in the equilibria involving the catalytically most important species, [(*PP*)ReH_4_]^+^, upon reaction with H_2_O, chiraphos, and H_2_.

To verify the potential involvement of high-valent Re species in the catalytic cycle, several control experiments were carried out using CA-**a** and CA-**11a**. Initially, solutions of the resting state of the catalysts, generated by the treatment of Re-**a** or Re-**b** with K[BPh_4_] during the hydrogenation of CA-**a** (*P*_H2_ = 1.5 MPa, 180 °C), were prepared separately, and the samples were analyzed directly by electrospray ionization-mass spectrometry (ESI-MS) (Supplementary Figs. 1–3). In both cases, [(*PP*)_2_Re^V^H_4_]^+^, which does not contain the oxygen atom of the original Re = O group anymore (Fig. 5a), was detected as the major species [*m*/*z* Calcd: 987.2571, Found: 987.2578 for *PP* = dppe^49^; *m*/*z* Calcd: 1043.3198, Found: 1043.3170 for *PP* = (2 *S*,3 *S*)-bis(diphenylphosphino)butane (chiraphos)]. The ^1^H NMR spectra in toluene-*d*_8_ revealed signals for the four hydrides of [(*PP*)_2_Re^V^H_4_]^+^ derived from Re-**b** as a quintet (*J*_P–H_ = 19.8 Hz) at –4.98 ppm^49^. This result suggests that these four hydrides are magnetically equivalent, and that they are coupled to four magnetically equivalent phosphorus atoms. This conclusion is supported by the presence of only one intensive, sharp ^31^P{^1^H} NMR singlet at 47.9 ppm. The structure should thus adopt *C*_2_-symmetry, wherein the four hydrides should be located in more apical than equatorial positions on the Re center, as the latter should be occupied by the four phosphorus atoms^49^. In contrast, the ESI-MS spectrum obtained from the solution of Re-**a** exhibited a set of unknown signals. One of these signals could represent [(*PP*)_2_Re^II^(OCO(CH_2_)_2_Ph)]^+^ (*m*/*z* Calcd: 1132.2872, Found: 1132.2894), which was not observed for the sample prepared from Re-**b**. This clearly suggests that Re-**b** is structurally more robust than Re-**a**, and consequently affords [(*PP*)_2_Re^V^H_4_]^+^ more efficiently.

K[BF_4_] was ineffective in the hydrogenation of CA-**a** when combined with Re-**a** (Supplementary Table 2; entry 7: *P*_H2_ = 4 MPa, 150 °C, 24 h). Likewise, only a marginal amount of AL-**a** (2%) was obtained when Re-**b** and K[BF_4_] were subjected to similar conditions ([Re-**b**]_0_ = 2.5 mM; *P*_H2_ = 4 MPa, 150 °C, 24 h). Even at higher temperature (180 °C, 12 h, *P*_H2_ = 1.5 MPa), AL-**a** was produced in only 10% yield, which is substantially less effective than using K[BPh_4_] (AL-**a**: 92%; ES-**a**: 3%; under otherwise identical conditions). The reaction mixture obtained at 180 °C showed non-negligible intensities of MS signals that are consistent with [(*PP*)_2_Re^IV^H_2_Cl]^+^ and [(*PP*)_2_Re^V^H_3_Cl]^+^ (*PP* = chiraphos; Supplementary Fig. 4), in addition to signals that were tentatively assigned to the resting state of the catalyst [(*PP*)_2_Re^V^H_4_]^+^. The former two structures should be of little relevance to the catalyst, considering that they are only produced in the presence of K[BF_4_]. Similarly, [(*PP*)_2_Re^V^H_4_]^+^ should not be considered as a catalytically active species, but rather as a resting state of the catalyst or precatalyst. When the hydrogenation of CA-**a** (*P*_H2_ = 2 MPa, 160 °C, 24 h) was carried out using ORe^V^Cl_3_(O = PPh_3_)((CH_3_)_2_S) (2 mol%: [Re]_0_ = 2.5 mM), chiraphos (4 or 6 mol%), and K[BPh_4_] (10 mol%), the yield of AL-**a** significantly decreased (22% and 0%, respectively) compared to that obtained from using 2 mol% of chiraphos (AL-**a**: 93%) (Fig. 5b; *cf*. Supplementary Tables 14 and 15). These results suggest that the most likely catalytically active species retains a 1:1 Re–chiraphos complexation, which could be readily generated by detachment of one chiraphos ligand from [(*PP*)_2_Re^V^H_4_]^+^.

Accordingly, Re-**b** should decompose slightly during the hydrogenation to form [(*PP*)_2_Re^V^H_4_]^+^ under concomitant release of chiraphos and the generation of phosphine-free Re species. Re black and/or Re nanoparticle are easily obtained from dehydrative reduction/decomposition of heterogeneous, high-valent Re-oxo species in the hydrogenation of CAs at 150–250 °C (*P*_H2_ = ~20 MPa)^50^. Therefore, a mercury test^51, 52^ was carried out, in which Hg(0) (338 mol%) was added during the hydrogenation of CA-**11a** with Re-**b** and KOAc (20 mol%) ([Re-**b**]_0_ = 5.0 mM in PhCF_3_, *P*_H2_ = 6 MPa, 160 °C, 168 h) to examine a potential catalysis by Re nanoparticles. However, the addition of Hg did not affect the catalytic activity of the hydrogenation or the hydrogenolysis (AL-**11b**: 33%; AL-**11c**: 63%; *cf*. Fig. 3c).

**Figure 6:**
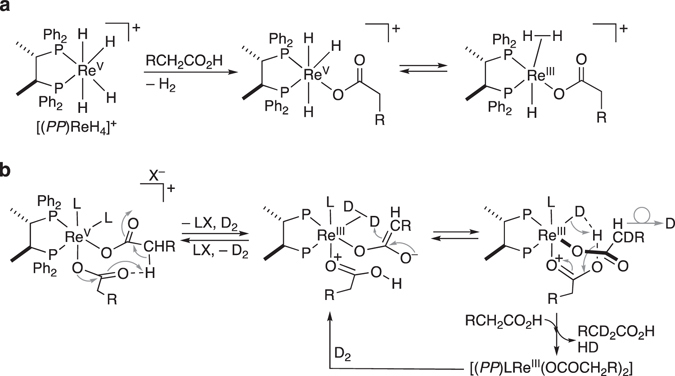
Proposed mechanism for the catalytic hydrogenation, and the deprotonation of α-C–H (R = PhCH_2_ or alkyl). (**a**) Formation of [(*PP*)ReH_3_(OCOCH_2_R)]^+^ for the “CA-self-induced hydrogenation of CA”. (**b**) Initial deprotonation and subsequent iterative H–D exchange reactions of CA, which form the catalytic cycle (L and X = D, Cl, or RCH_2_CO_2_). The CA (R′CO_2_H) and carboxylate (R′CO_2_^–^) (irrespective of how many deuterium atoms are incorporated at the α-position, i.e., α-*C*-*d*_0_, α-*C*-*d*_1_, or α-*C*-*d*_2_) attached to a Re center could mutually interchange by intramolecular proton transfer [(R′CO_2_H)Re(OCOR′) $\leftrightarrows$ (R′CO_2_)Re(HOCOR′)] or intermolecularly exchange with free CA [e.g. Re(OCOR′) + RCH_2_CO_2_H $\leftrightarrows$ Re(OCOCH_2_R) + R′CO_2_H].

When H_2_O (100, 200, or 500 mol% with respect to CA-**a**) was added prior to starting the hydrogenation of CA-**a** with Re-**b**, the reaction rate for the formation of AL-**a** (99% after 12 h) observed in the absence of such a pre-addition of H_2_O was considerably retarded (AL-**a**: 71%, 59%, and 34%, respectively). However, the integrity of the catalysis was sustained, and extending the reaction time to 24 h increased the yield of AL-**a** significantly (96%, 97%, and 50%, respectively) (Fig. 5c). This result implies that Re = O species should only play a peripheral role in the catalysis, considering that H_2_O should shift the reaction equilibrium from a ReH_2_ species to a Re = O structure (Fig. 5d)^50, 53^. All control experiments suggest that the mononuclear Re species [(*PP*)Re^V^(*η* ^1^-H)_4_]^+^ represents an important precursor (albeit presumably outside the catalytic cycle) that subsequently affords the cationic mononuclear Re-carboxylate [(*PP*)Re^V^(*η* ^1^-H)_3_(OCO(CH_2_)_2_Ph)]^+^ upon reaction with CA-**a** (Fig. 6a) in an initial critical point of the catalytic cycle. This interpretation is consistent with our previous observations, which identified the related cationic metal carboxylate [(*PP*)Ru^II^(OCO(CH_2_)_2_Ph)]^+^ as the key intermediate in a catalytic cycle involving the “CA-self-induced hydrogenation of CA”^18^. However, at this point, a catalytic involvement of [(*PP*)Re^III^(*η* ^1^-H)(*η* ^2^-H_2_)(OCO(CH_2_)_2_Ph)]^+^ (Fig. 6a), which could also be derived from [(*PP*)Re^III^(*η* ^1^-H)_2_]^+^ in the presence or absence of *η* ^2^-H_2_ coordination, cannot be ruled out with certainty.

In contrast, a treatment of catalytic Re-**b** (6 mol%) with KOAc (30 mol%) and CA-**a** in the absence of H_2_ in excess Ac_2_O (160 °C, 4 h), which promotes the Dakin–West reaction, afforded a sample solution that showed ESI-MS signals consistent with [(*PP*)Re^V^Cl(OCO(CH_2_)_2_Ph)_3_]^+^ (*m*/*z* Calcd: 1095.2714, Found: 1095.2756), i.e., a dehydrated form of the corresponding Re = O species (Supplementary Fig. 5). A similar set of MS signals was observed by sampling a reaction mixture obtained from treating Re-**b** (0.4 mol%) with K[BPh_4_] (2 mol%), CA-**a**, and D_2_ (0.1 MPa) at 180 °C for 18 h in the absence of solvent (Supplementary Fig. 6). Therein, the detected [(*PP*)Re^V^Cl(OCO(CH_2_)_2_Ph)_3_]^+^ should not be a catalytically active species for the H–D exchange, considering that deuterium was not incorporated in the three carboxylates. In sharp contrast, when Re-**a** was used instead of Re-**b** under otherwise identical conditions (*P*_D2_ = 0.1 MPa), [(*PP*)Re^V^Cl(OCO(CH_2_)_2_Ph)_3_]^+^ was not detected (*PP* = dppe), and the typical separation pattern of the MS signals corresponding to Re complexes was barely observed, which suggests ready decomposition of the Re complexes at 180 °C to Re black or nanoparticles (Supplementary Fig. 7). In a similar fashion, the use of Re-**a** under comparable conditions (in the absence of H_2_) in the Dakin–West reaction resulted only in a significant detachment of dppe from the Re center (Supplementary Fig. 8). Indeed, deuteration at the α-position of CA-**a** hardly proceeded (deuteration: ~3%) using Re-**a** at *P*_D2_ = 0.1 MPa, whereas at *P*_D2_ = 1.0 MPa ([Re-**a**]_0_ = 5 mM in toluene, 180 °C, 36 h), AL-**a**-*d*_3–4_ (>95%) was obtained as the major product (deuteration: C1 = 85%, C2 = ~92%). To summarize the behavior of Re-**a**: in the absence of H_2_ or at low H_2_ pressure (*P*_H2_ ~0.1 MPa), a 1:1 Re–dppe complexation in Re-**a** is rather unstable at 160–180 °C, while a good yield of [(dppe)_2_Re^V^H_4_]^+^ is obtained at higher H_2_ pressure (e.g. *P*_H2_ = 1.0 MPa). These experiments lead to the provisional conclusion that an H–D exchange reaction of CA-**a** should – at least partially – involve a “CA-self-induced deprotonation of CA” promoted by an intramolecular hydrogen transfer in [(*PP*)Re^III^L(OCO(CH_2_)_2_Ph)_2_] (Fig. 6b). However, a definite conclusion, excluding e.g. hitherto unobserved catalytically active species, should require further detailed experiments and analyses.

In conclusion, the use of Re^V^(=O)-diphosphine complexes provides a novel operationally simple concept for the selective activation and functionalization of CH_*n*_CO_2_H groups (*n* = 1–3). This method combines an unprecedented substrate scope with outstanding functional group compatibility. Furthermore, sulfur components, i.e., poisons for conventional low-valent transition metal hydrogenation catalysts, do not affect the catalytic performance. High-valent transition metal complexes have traditionally been underestimated in the context of the hydrogenation of CAs, hydrogenolysis of alcohols, C–H bond functionalization, and hydrogenation of compounds with more reactive unsaturated bonds (e.g. C=C and C=O)^33^. Our results represent an important step toward the development of new catalyst systems for carbon^54^ and hydrogen management^55^, that allow the formation of asymmetric carbon–carbon bonds and hydrogenations using biomass-derived renewable feedstocks. Rhenium is one of the rarest elements in the Earth’s crust, and a commercial price of Re source we used to synthesize different Re complexes is more expensive than, or comparable to, that of a Ru source, from which our previous Ru complexes were prepared^18^, and is ca. one forth that of rhodium- and iridium sources used for CA hydrogenation^36^. Since Co(BF_4_)_2_ used by Elsevier/Bruin^19^ is much economical, an effort to lower a Re load in hydrogenation is ongoing research in our laboratory.

## Methods

### Representative hydrogenation of CA-a with Re-b

3-Phenylpropanoic acid (CA-**a**) (0.5 mmol, 75.0 mg), KBPh_4_ (0.05 mmol, 17.9 mg), ReOCl_3_[(*S*,*S*)-Chiraphos] (Re-**b**) (0.01 mmol, 7.3 mg) and a magnetic stirring bar, were placed in a dried glass tube that was inserted in an autoclave, which was purged with Ar gas several times. Anhydrous THF (4.0 mL) was added under a continuous flow of Ar, and the autoclave was purged five times with H_2_ (1 MPa). The autoclave was pressurized with H_2_ (*P*_H2_ = 2 MPa) at 25 °C and heated to 160 °C, where the mixture was stirred (500 rpm) for 24 h. Then, the autoclave was cooled to 0 °C, before the reaction mixture was transferred to a 100 mL round bottom flask containing CHCl_3_. The mixture was concentrated (~30 mmHg, 40 °C), and the residue was dissolved in CDCl_3_ and analyzed by ^1^H NMR spectroscopy. Yields of 3-phenyl-1-propanol (AL-**a**) (93%) and 1-(3-phenylpropyl)-3-phenylpropanoate (ES-**a**) (~1%) were calculated based on the integration ratio of their signals relative to the internal standard mesitylene and by GC-MS analysis, respectively.

## Electronic supplementary material

Supplementary Information

## References

[1] Wang W-H, Himeda Y, Muckerman JT, Manbeck GF, Fujita E (2015). CO_2_ hydrogenation to formate and methanol as an alternative to photo- and electrochemical CO_2_ reduction. Chem. Rev..

[2] Liu Q, Wu L, Jackstell R, Beller M (2015). Using carbon dioxide as a building block in organic synthesis. Nat. Commun..

[3] Otto A, Grube T, Schiebahn S, Stolten D (2015). Closing the loop: captured CO_2_ as a feedstock in the chemical industry. Energy Environ. Sci..

[4] Ostapowicz TG, Schmitz M, Krystof M, Klankermayer J, Leitner W (2013). Carbon dioxide as a C1 building block for the formation of carboxylic acids by formal catalytic hydrocarboxylation. Angew. Chem. Int. Ed..

[5] Masuda Y, Ishida N, Murakami M (2015). Light-driven carboxylation of *o*‑alkylphenyl ketones with CO_2_. J. Am. Chem. Soc..

[6] Arai T, Sato S, Morikawa T (2015). A monolithic device for CO_2_ photoreduction to generate liquid organic substances in a single-compartment reactor. Energy Environ. Sci..

[7] Sato S (2011). Selective CO_2_ conversion to formate conjugated with H_2_O oxidation utilizing semiconductor/complex hybrid photocatalysts. J. Am. Chem. Soc..

[8] Ruppert AM, Weinberg K, Palkovits R (2012). Hydrogenolysis goes bio: from carbohydrates and sugar alcohols to platform chemicals. Angew. Chem. Int. Ed..

[9] Corma A, Iborra S, Velty A (2007). Chemical routes for the transformation of biomass into chemicals. Chem. Rev..

[10] Bozell JJ, Petersen GR (2010). Technology development for the production of biobased products from biorefinery carbohydrates—the US Department of Energy’s “Top 10” revisited. Green Chem..

[11] Olah GA, Prakash GKS, Goeppert A (2011). Anthropogenic chemical carbon cycle for a sustainable future. J. Am. Chem. Soc..

[12] Teichmann D, Arlt W, Wasserscheid P, Freymann R (2011). A future energy supply on liquid organic hydrogen carriers (LOHC). Energy Environ. Sci..

[13] Stöcker M (2008). Biofuels and biomass-to-liquid fuels in the biorefinery: catalytic conversion of lignocellulosic biomass using porous materials. Angew. Chem. Int. Ed..

[14] Watanabe R, Yamauchi M, Sadakiyo M, Abe R, Takeguchi T (2015). CO_2_-free electric power circulation via direct charge and discharge using the glycolic acid/oxalic acid redox couple. Energy Environ. Sci..

[15] Morita Y, Yamamoto T, Nagai H, Shimizu Y, Kanai M (2015). Chemoselective boron-catalyzed nucleophilic activation of carboxylic acids for Mannich-type reactions. J. Am. Chem. Soc..

[16] Wang D-H, Engle KM, Shi B-F, Yu J-Q (2010). Ligand-enabled reactivity and selectivity in a synthetically versatile aryl C–H olefination. Science.

[17] Giri R (2007). Palladium-catalyzed methylation and arylation of sp^2^ and sp^3^ C-H bonds in simple carboxylic acids. J. Am. Chem. Soc..

[18] Naruto M, Saito S (2015). Cationic mononuclear ruthenium carboxylates as catalyst prototypes for self-induced hydrogenation of carboxylic acids. Nat. Commun..

[19] Korstanje TJ, Vlugt JIVD, Elsevier CJ, Bruin BD (2015). Hydrogenation of carboxylic acids with a homogeneous cobalt catalyst. Science.

[20] Cui X, Li Y, Topf C, Junge K, Beller M (2015). Direct Ruthenium-catalyzed hydrogenation of carboxylic acids to alcohols. Angew. Chem. Int. Ed..

[21] Stein Tv (2014). Highly versatile catalytic hydrogenation of carboxylic and carbonic acid derivatives using a Ru-triphos complex: molecular control over selectivity and substrate scope. J. Am. Chem. Soc..

[22] Geilen FMA, Engendahl B, Hölscher M, Klankermayer J, Leitner W (2011). Selective homogeneous hydrogenation of biogenic carboxylic acids with [Ru(TriPhos)H]^+^: A mechanistic study. J. Am. Chem. Soc..

[23] Pritchard J, Filonenko GA, Putten RV, Hensen EJM, Pidko EA (2015). Heterogeneous and homogeneous catalysis for the hydrogenation of carboxylic acid derivatives: history, advances and future directions. Chem. Soc. Rev..

[24] Noble A, McCarver SJ, MacMillan DWC (2015). Merging photoredox and nickel catalysis: decarboxylative cross-coupling of carboxylic acids with vinyl halides. J. Am. Chem. Soc..

[25] Chu L, Ohta C, Zuo Z, MacMillan DWC (2014). Carboxylic acids as a traceless activation group for conjugate additions: a three-step synthesis of (±)-Pregabalin. J. Am. Chem. Soc..

[26] Gooßen LJ, Rodríguez N, Gooßen K (2008). Carboxylic acids as substrates in homogeneous catalysis. Angew. Chem. Int. Ed..

[27] Gooßen LJ, Deng G, Levy LM (2006). Synthesis of biaryls via catalytic decarboxylative coupling. Science.

[28] Babich IV, Moulijn JA (2003). Science and technology of novel processes for deep desulfurization of oil refinery streams: a review. Fuel.

[29] Kulkarni PS, Afonso CAM (2010). Deep desulfurization of diesel fuel using ionic liquids: current status and future challenges. Green Chem..

[30] Bursten BE, Cayton RH (1987). Electronic connections between exceptional low-valent and high-valent organometallic compounds: the case of CpM(L)R, (M = W, Re; L = NO, O; R = Alkyl). Organometallics.

[31] Owens GS, Arias J, Abu-Omar MM (2000). Rhenium oxo complexes in catalytic oxidations. Catal. Today.

[32] Korstanje TJ, Gebbink RJMK (2012). Catalytic oxidation and deoxygenation of renewables with rhenium complexes. Top. Organomet. Chem..

[33] Sousa SCA, Cabrita I, Fernandes AC (2012). High-valent oxo-molybdenum and oxo-rhenium complexes as efficient catalysts for X–H (X = Si, B, P and H) bond activation and for organic reductions. Chem. Soc. Rev..

[34] Preliminary results of this report (including Re complexes Re-**b**–**f**) were used in a patent application, see: Saito, S. Noyori, R., Agrawal, S. & Naruto, M. *JP patent* Appl. #2013-268047, Filed: Dec 25, 2013.

[35] Sigouin O, Beauchamp AL (2005). Oxo-rhenium(V) complexes with analogs of bis(diphenylphosphino)ethane. Inorg. Chim. Acta.

[36] Brewster TP, Miller AJM, Heinekey DM, Goldberg KI (2013). Hydrogenation of carboxylic acids catalyzed by half-sandwich complexes of iridium and rhodium. J. Am. Chem. Soc..

[37] Touchy AS, Kon K, Onodera W, Shimizu K (2015). Unprecedented reductive esterification of carboxylic acids under hydrogen by reusable heterogeneous platinum catalysts. Adv. Synth. Catal..

[38] Kon K, Onodera W, Takakusagi S, Shimizu K (2014). Hydrodeoxygenation of fatty acids and triglycerides by Pt-loaded Nb_2_O_5_ catalysts. Catal. Sci. Technol..

[39] He D-H, Wakasa N, Fuchikami T (1995). Hydrogenation of carboxylic acids using bimetallic catalysts consisting of group 8 to 10, and group 6 or 7 metals. Tetrahedron Lett..

[40] Yoshino K, Kajiwara Y, Takaishi N, Inamoto Y, Tsuji J (1990). Hydrogenation of carboxylic acids by rhenium-osmium bimetallic catalyst. J. Am. Oil. Chem. Soc..

[41] Takeda Y, Nakagawa Y, Tomishige K (2012). Selective hydrogenation of higher saturated carboxylic acids to alcohols using a ReO_x_–Pd/SiO_2_ catalyst. Catal. Sci. Technol..

[42] Rozmysłowicz B (2015). Selective hydrogenation of fatty acids to alcohols over highly dispersed ReO_*x*_/TiO_2_ catalyst. J. Catal..

[43] Toyao T (2017). TiO_2_-Supported Re as a general and chemoselective heterogeneous catalyst for hydrogenation of carboxylic acids to alcohols. Chem. Eur. J..

[44] Broadbent HS, Bartley WJ (1963). Rhenium catalysts. VII. rhenium(VI) oxide. J. Org. Chem..

[45] Tegnér C (1952). On the reaction between methyllithium and carboxylic acids. Acta Chim. Scand..

[46] Buchanan GL (1988). The Dakin–West reaction. Chem. Soc. Rev..

[47] Tran K-V, Bickar D (2006). Dakin–West Synthesis of β-Aryl Ketones. J. Org. Chem..

[48] Burgstahler AW, Worden LR (1966). COUMARONE [benzofuran]. Org. Synth..

[49] Freni M, Demichelis R, Giusto D (1967). Hydrido and halogenohydrido complexes of rhenium(III). J. Inorg. Nucl. Chem..

[50] Broadbent HS, Campbell GC, Bartley WJ, Johnson JH (1959). Rhenium and its compounds as hydrogenation catalysts. III. rhenium heptoxide. J. Org. Chem..

[51] Miura T, Held IE, Oishi S, Naruto M, Saito S (2013). Catalytic hydrogenation of unactivated amides enabled by hydrogenation of catalyst precursor. Tetrahedron Lett..

[52] Miura, T., Naruto, M., Toda, K., Shimomura, T. & Saito, S. Multifaceted catalytic hydrogenation of amides via diverse activation of a sterically confined bipyridine–ruthenium framework. *Sci. Rep*. **7**, 1586, doi:10.1038/s41598-017-01645-z (2017). 10.1038/s41598-017-01645-z PMC5434022 28512286

[53] Belousov VM, Palchevskaya TA, Bogutskaya LV, Zyuzya LA (1990). Properties of homogeneous and heterogeneous rhenium catalysts in the hydrogenation of nitro compounds. J. Mol. Catal..

[54] Marks TJ (2001). Catalysis research of relevance to carbon management: progress, challenges, and opportunities. Chem. Rev..

[55] Takada Y, Iida M, Iida K, Miura T, Saito S (2016). Versatile ruthenium complex “RuPCY” for directed catalytic hydrogen management in organic synthesis. J. Org. Syn. Chem. Jpn..

