# Supplementary material for: Catalytic transformation of functionalized carboxylic acids using multifunctional rhenium complexes
Source: Sci Rep. 2017 Jun 13;7:3425. doi: 10.1038/s41598-017-03436-y (PMC5469866; doi:10.1038/s41598-017-03436-y)
Supplement: Supplementary file 1 — Supplementary Information [file 41598_2017_3436_MOESM1_ESM.pdf]

Supplementary Information

Catalytic transformation of functionalized carboxylic acids using multifunctional rhenium complexes

Masayuki Naruto, Santosh Agrawal, Katsuaki Toda, and Susumu Saito*
Graduate School of Science, Nagoya University, Chikusa, Nagoya 464-8602, Japan

**Correspondence to*: saito.susumu@f.mbox.nagoya-u.ac.jp

Table of Contents

1. General methods and materials 2

2. Supplementary Tables 4

3. Supplementary Figures 24

4. Experimental procedures 32

4.1. Hydrogenation experiments 32

4.2. Deuteration experiments 34

4.3. Unsymmetrical ketones synthesis 35

4.4. Intramolecular aldol condensation 37

4.5. Investigation of Re species generated upon hydrogenation 38

4.6. Investigation of Re species generated upon deuteration reaction 39

4.7. Investigation of Re species generated upon unsymmetrical ketones synthesis 40

4.8. Precatalyst preparation 41

4.9. Substrate preparation for hydrogenation 47

4.10. New compounds obtained in hydrogenation and deuteration experiments 50

5. Spectral data 53

5.1. New Re complexes 53

5.2. NMR spectra of substrates synthesized 57

5.3. NMR spectra of new compounds obtained in hydrogenation and deuteration experiments 62

6. References 68

1. General methods and materials

General methods

All experiments were performed under an inert gas unless otherwise noted. ^{1}H NMR spectra were measured on JEOL ECA–600 (600 MHz), JEOL ECA–500 (500 MHz) at ambient temperature. Data were recorded as follows: chemical shift in ppm from internal tetramethylsilane (δ 0 ppm) or residual peak of DMSO-d_6 (δ 2.50 ppm) and methylene chloride-d_2 (δ 5.32 ppm), multiplicity (bs = broad singlet, s = singlet, d = doublet, t = triplet, q = quartet, m = multiplet), coupling constant (Hz), integration, and assignment. ^{13}C NMR spectra were measured on JEOL ECA–600 (150 MHz), JEOL ECA–500 (126 MHz) at ambient temperature. Chemical shifts were recorded in ppm from the solvent resonance employed as the internal standard (chloroform-*d* at 77.00 ppm or DMSO-d_6 at 39.50 ppm or methylene chloride-d_2 at 53.50 ppm). ^{31}P NMR spectra were measured on JEOL ECA–600 (243 MHz), JEOL ECA–500 (202 MHz) at ambient temperature. Chemical shifts were recorded in ppm from the solvent resonance employed as the external standard (phosphoric acid (85 wt% in H_2O) at 0.0 ppm). High–resolution mass spectra (HRMS) were obtained from JEOL JMS700 (FAB), BRUKER micrOTOF-QII (ESI). IR spectra were obtained from JASCO FT/IR6100. For thin–layer chromatography (TLC) analysis through this work, Merck precoated TLC plates (silica gel 60 GF254 0.25 mm) were used. The products were purified by preparative column chromatography on silica gel 60 N (spherical, neutral) (40–100 μm; Kanto).

Materials

Methyl trioxorhenium (VII) (Re-**h**), iododioxobis(triphenylphosphine)rhenium (V) (Re-**i**), oxotrichloro[(dimethylsulfide)triphenylphosphineoxide]rhenium (Re-**j**), trichlorooxobis(triphenylphosphine)rhenium (V) (Re-**k**), oxotrichloro[bis(diphenylphosphino)methane]rhenium (V) (Re-**c**), (acetonitrile)tricholorobis(triphenylphosphine)rhenium (III) (Re-**m**), sodium tetraphenylborate, potassium tetraphenylborate, cesium tetraphenylborate, lithium tetraphenylborate tris(1,2-dimethoxyethane), (2*S*,3*S*)-(–)-bis(diphenylphosphino)butane ((*S*,*S*)-Chiraphos), (1*S*,2*S*)-(+)-bis[(2-methoxyphenyl)phenylphosphino]ethane ((*S*,*S*)-DIPAMP), 4,5-bis(diphenylphosphino)-9,9-dimethylxanthene,

1,1,1-tris(diphenylphosphinomethyl)ethane (Triphos), 4-phenylbutyric acid (CA-**e**), Ibuprofen (CA-**f**), 16-hydroxyhexadecanoic acid (CA-**g**), 3-methoxyphenylacetic acid (CA-**i**), 4-(2-thienyl)butyric acid (CA-**u**), 3-(4-methylthiophenyl)propionic acid (CA-**x**), 3-(2-fryl)propionic acid (CA-**y**), monomethyl suberate (CA-**1**), 4,6-dimethyldibenzothiophene (TH-**c**), 4-methyldibenzothiophene (96 %) (TH-**b**), 2,5-dimethoxytetrahydrofuran, 1,2-dichloroethane, *L*-phenylalanine, methyl nonanoate, α, α, α-trifluorotoluene and diethylene glycol dimethyl ether (diglyme) (anhydrous) were purchased from Aldrich. Trifluorometanesulfonic acid, cyclohexanepropionic acid (CA-**d**), phenoxyacetic acid (CA-**h**), 3,4,5-trimethoxyphenylacetic acid (CA-**j**), 4-chlorocinnamic acid (CA-**l**), 4-bromophenylacetic acid (CA-**b**), 1-adamantanecarboxylic acid (CA-**2**), 5-(benzoylamino)valeric acid (CA-**3**), 3-(3-pyridyl)propionic acid (CA-**z**), 4-(trifluoromethyl)benzoic acid (CA-**5**), 4-chlorobenzoic acid (CA-**6**), 4-(methylthio)benzoic acid (CA-**7**), 4-dimethylaminobenzoic acid (CA-**8**), benzofuran-2-carboxylic acid (CA-**11**), benzofuran (FR-**12**), dibenzothiophene (TH-**a**), benzo[b]thiophene (TH-**e**), dibutyl sulfide (TH-**d**), acetic anhydride, benzoic anhydride, β -alanine, methyl terephthalaldehydate, 2-methyltetrahydrofuran, 1,1,2,2-tetrachloroethane, 3-phenylpropionaldehyde (AD-**a**), and 3-phenylpropionic acid methyl ester were purchased from TCI, Ltd. *n*-Nonanoic acid (CA-**c**), diphenylacetic acid (CA-**o**), *trans*-cinnanic acid (CA-**k**), cyclohexanecarboxylic acid (CA-**v**), *n*-nonyl alcohol (AL-**c**), pivaloyl chloride, sodium acetate, potassium acetate, THF (anhydrous), benzene (anhydrous), toluene (anhydrous), cyclohexane (anhydrous), dichloromethane, ethanol, chloroform, mesitylene, Na_2SO_4, NaOH, DMSO, diethyl ether, *N*,*N*-dimethylformamide dehydrated, acetonitrile dehydrated and methanol dehydrated were purchased from Kanto Chemicals, Ltd. 3-Phenylpropionic acid (CA-**a**), 3,5-dimethoxybenzoic acid (CA-**10**), 3,5-dichlorobenzoic acid (CA-**9**), 2-furancarboxylic acid (CA-**4**), propionic anhydride, *D*-α-alanine, *L*-leucine, potassium tetrafluoroborate, 1,4-dioxane (anhydrous), acetic acid, ethyl acetate and hexane and ethanol dehydrated were purchased from Wako Pure Chemical industries, Ltd. α-Methylcinnamic acid (CA-**m**) was purchased from Across Organics, Ltd. Benzoic acid (CA-**w**) was purchased from Nacalai. Na(acac)•H_2O was purchased from Alfa Aesar. Malonic acid was purchased from SAJ. $CDCl_3$, DMSO-d_6 and CD_2Cl_2 were purchased from Cambridge Isotope Laboratories, Inc. Hydrogen gas and deuterium gas were purchased from Alpha System. These chemicals were used without further purification. $ReOCl_3(OPPh_3)(SMe_2)$ was

prepared according to the reported procedures.[1]

2. Supplementary Tables

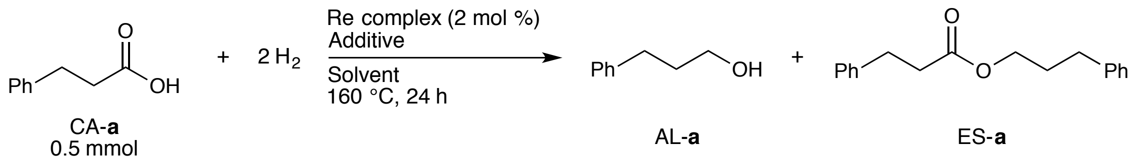

Entry	Re complex	Additive (mol %)	Solvent (mL)	H_2 (MPa)	Yield (%)	
					AL-**a**	ES-**a**
1	Re-**c**	$NaBPh_4$ (10 mol %)	toluene (2.0 mL)	8	54	11
2	Re-**h**	CF_3SO_3H (4 mol %)	toluene (2.0 mL)	8	trace	5
3	Re-**i**	$NaBPh_4$ (10 mo l%)	toluene (2.0 mL)	8	14	7
4	Re-**j**	$NaBPh_4$ (10 mol %)	toluene (4.0 mL)	8	12	trace
5	Re-**k**	$NaBPh_4$ (10 mol %)	toluene (4.0 mL)	8	62	9
6	Re-**k**	none	toluene (4.0 mL)	8	trace	trace
7	Re-**a**	$NaBPh_4$ (10 mol %)	toluene (4.0 mL)	8	>98	<1
8	Re-**c**	$NaBPh_4$ (10 mol %)	toluene (4.0 mL)	8	72	5
9	Re-**d**	$NaBPh_4$ (10 mol %)	toluene (4.0 mL)	8	46	6
10	Re-**e**	$NaBPh_4$ (10 mol %)	toluene (4.0 mL)	8	89	5
11	Re-**f**	$NaBPh_4$ (10 mol %)	toluene (4.0 mL)	8	34	5
12	Re-**l**	$NaBPh_4$ (10 mol %)	toluene (4.0 mL)	8	9	4
13	Re-**m**	$NaBPh_4$ (10 mol %)	toluene (4.0 mL)	8	37	5
14	Re-**a**	$KBPh_4$ (10 mol %)	THF (4.0 mL)	2	35	1
15	Re-**b**	$KBPh_4$ (10 mol %)	THF (4.0 mL)	2	93	trace
16	Re-**g**	$KBPh_4$ (10 mo l%)	THF (4.0 mL)	2	32	1

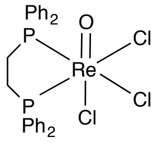

Re-**a**

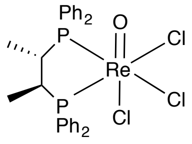

Re-**b**

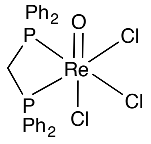

Re-**c**

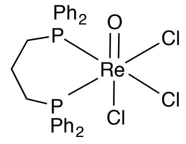

Re-**d**

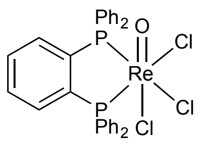

Re-**e**

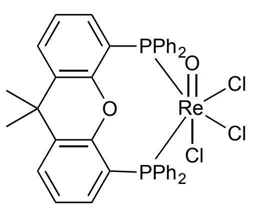

Re-**f**

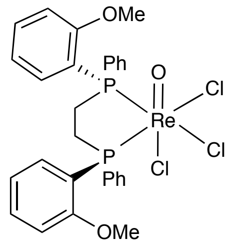

Re-**g**

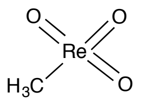

Re-**h**

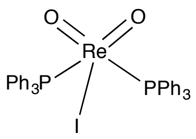

Re-**i**

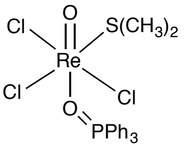

Re-**j**

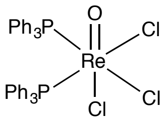

Re-**k**

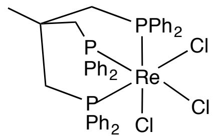

Re-**l**

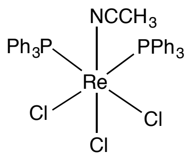

Re-**m**

Supplementary Table 1 | Re complexes tested in the initial screening. Unless otherwise specified, the reactions were carried out with Re complex:additive:CA-**a** (mol%) = 2:10:100, T = 160 ºC, and t = 24 h. ^{1}H NMR yields were determined based on the integral ratio of the signals of products and internal standard (mesitylene).

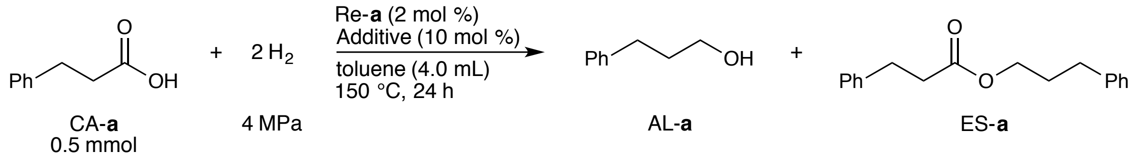

Entry	Additive	Yield (%)	
		AL-**a**	ES-**a**
1	Na(acac)	6	1
2	NaOAc	11	1
3	$NaBPh_4$	55	4
4	$KBPh_4$	80	2
5	$CsBPh_4$	60	trace
6[a]	$LiBPh_4$	14	4
7	KBF_4	0	0
8[b]	$KBPh_4$	97	<2

Supplementary Table 2 | Additives tested for optimization of hydrogenation conditions. Unless otherwise specified, the reactions were carried out with Re complex:additive:CA-**a** (mol%) = 2:10:100, P_{H2} = 4 MPa, T = 150 ºC, and t = 24 h. ^{1}H NMR yields were determined based on the integral ratio of the signals of products and internal standard (mesitylene). [a]$LiBPh_4$•3($CH_3OCH_2CH_2OCH_3$) was used. [b]Reaction time was 30 h.

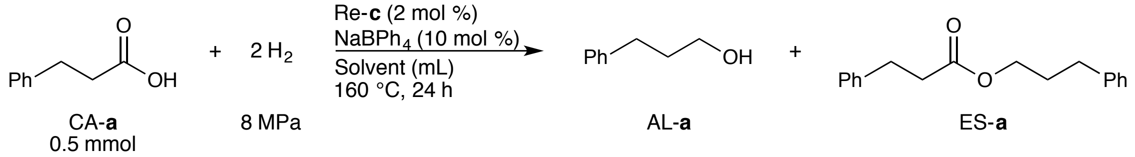

Entry	Solvent (mL)	Yield (%)	
		AL-**a**	ES-**a**
1	toluene (0.5 mL)	4	29
2	toluene (1.0 mL)	18	25
3	toluene (2.0 mL)	54	11
4	toluene (3.0 mL)	69	6
5	toluene (4.0 mL)	72	5
6[a]	toluene (4.0 mL)	>95	trace
7	$(C_6H_5)CF_3$ (4.0 mL)	35	4
8	1,4-dioxane (4.0 mL)	31	trace
9	toluene/cyclohexane (4.0 mL, 3:1 v/v)	64	6
10	THF (4.0 mL)	83	0
11	diglyme (4.0 mL)	0	0
12	2-methylTHF (4.0 mL)	45	0
13	toluene/H_2O (4.0 mL, 3:1 v/v)	25	4
14	THF/H_2O (4.0 mL, 3:1 v/v)	0	0
15	DMSO-d_6 (4.0 mL)	0	0

Supplementary Table 3 | Solvents tested for optimization of hydrogenation conditions. Unless otherwise specified, the reactions were carried out with Re complex:additive:CA-**a** (mol%) = 2:10:100, P_{H2} = 8 MPa, T = 160 ºC, and t = 24 h. ^{1}H NMR yields were determined based on the integral ratio of the signals of products and internal standard (mesitylene). [a]Reaction time was 36 h.

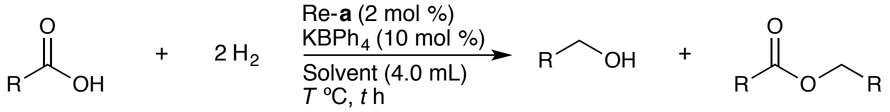

CA
0.5 mmol

4 MPa

AL

ES

Entry	Carboxylic acid (CA)	*T* °C, *t* h<br>Solvent	Product alcohol (AL)	Yield (%)<br>AL (ester (ES))
1	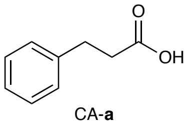 CA-**a**	150 °C, 30 h<br>toluene	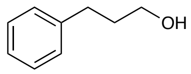 AL-**a**	97 (<2)
2	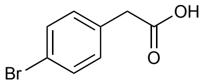 CA-**b**	160 °C, 24 h<br>THF	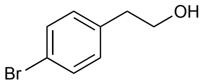 AL-**b**	94 (<3), 94[a]
3	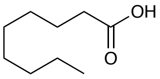 CA-**c**	150 °C, 30 h<br>toluene	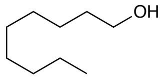 AL-**c**	99 (trace)
4	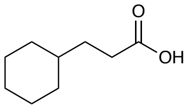 CA-**d**	150 °C, 24 h<br>toluene	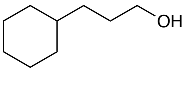 AL-**d**	99 (trace)
5	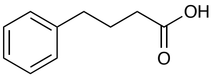 CA-**e**	150 °C, 30 h<br>toluene	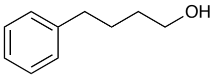 AL-**e**	73 (3)
6	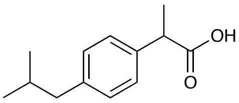 CA-**f**	160 °C, 24 h<br>toluene	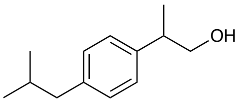 AL-**f**	>99 (0), >95[a]
7	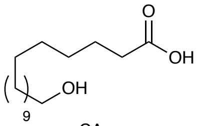 CA-**g**	150 °C, 24 h<br>toluene	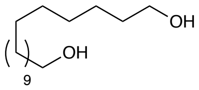 AL-**g**	76 (<10)
8	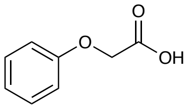 CA-**h**	160 °C, 24 h<br>THF	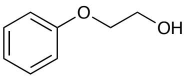 AL-**h**	99 (trace), 87[a]
9	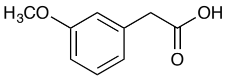 CA-**i**	160 °C, 24 h<br>THF	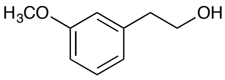 AL-**i**	98 (<4), 87[a]

Entry	Carboxylic acid (CA)	*T* °C, *t* h<br>Solvent	Product alcohol (AL)	Yield (%)<br>AL (ester (ES))
10	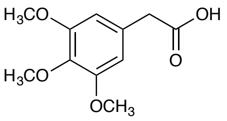<br>CA-**j**	160 °C, 24 h<br>THF	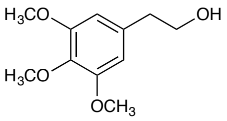<br>AL-**j**	>99 (<3), 95[a]
11	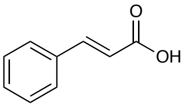<br>CA-**k**	160 °C, 24 h<br>toluene	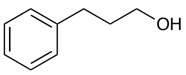<br>AL-**k**	>95 (<3)
12	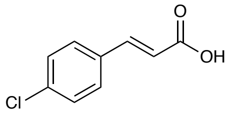<br>CA-**l**	160 °C, 24 h<br>toluene	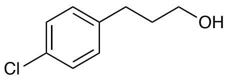<br>AL-**l**	90 (4), 76[a]
13	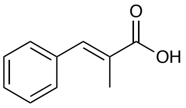<br>CA-**m**	160 °C, 24 h<br>THF	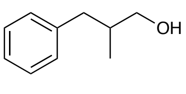<br>AL-**m**	90 (0)
14	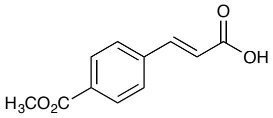<br>CA-**n**	160 °C, 36 h<br>THF	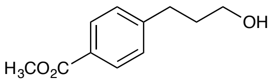<br>AL-**n**	>90 (0), 87[a]
15	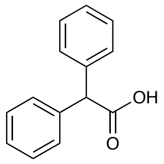<br>CA-**o**	160 °C, 24 h<br>toluene	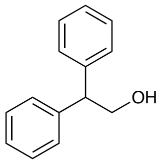<br>AL-**o**	90 (<3)
16	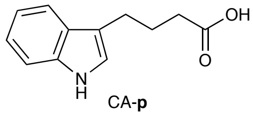<br>CA-**p**	160 °C, 36 h<br>THF (4 mL)	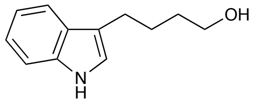<br>AL-**p**	99 (0), 92[a]
17	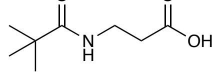<br>CA-**q**	160 °C, 36 h<br>THF	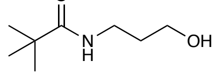<br>AL-**q**	71[a]
18[b]	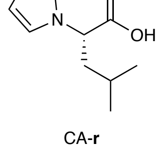<br>CA-**r**	160 °C, 36 h<br>THF	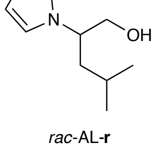<br>*rac*-AL-**r**	64[a]

Entry	Carboxylic acid (CA)	*T* °C, *t* h<br>Solvent	Product alcohol (AL)	Yield (%)<br>AL (ester (ES))
19[b]	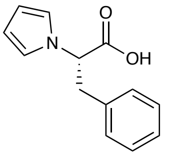<br>CA-**s**	160 °C, 36 h<br>THF	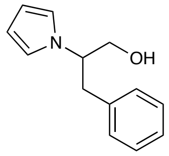<br>*rac*-AL-**s**	81[a]
20	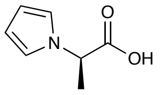<br>CA-**t**	160 °C, 24 h<br>THF	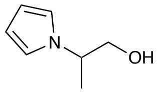<br>*rac*-AL-**t**	92 (0), 77[a]
21	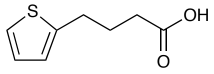<br>CA-**u**	160 °C, 24 h<br>toluene	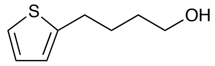<br>AL-**u**	67 (3)
22	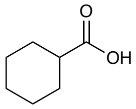<br>CA-**v**	150 °C, 24 h<br>toluene	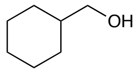<br>AL-**v**	58 (3)
23	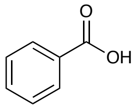<br>CA-**w**	180 °C, 24 h<br>toluene	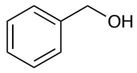<br>AL-**w**	29 (1)

Supplementary Table 4 | Substrate screening using Re-**a** as catalyst precursor. Unless otherwise specified, the reactions were carried out with Re complex:additive:CA (mol%) = 2:10:100, P_{H2} = 4 MPa. ^{1}H NMR yields were determined based on the integral ratio of the signals of products and internal standard (mesitylene). [a]Isolated yield of alcohols. [b]THF (6.0 mL) was used.

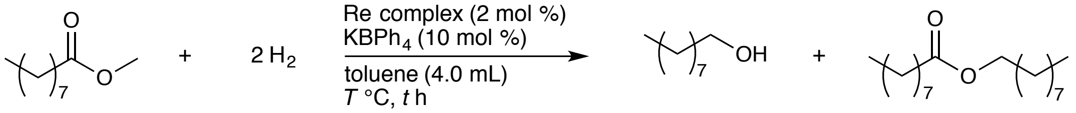

0.5 mmol AL-**c** ES-**c**

Entry	Re complex	H_2 (MPa)	*T* (°C)	*t* (h)	Yield (%)	
					AL-**c**	ES-**c**
1	Re-**a**	4	160	24	8	0
2	Re-**b**	4	160	24	9	0
3	Re-**b**	4	180	12	9	0
4	Re-**b**	2	180	12	9	0

Supplementary Table 5 | Hydrogenation of methyl nonanate using Re-**a** and Re-**b**. Unless otherwise specified, the reactions were carried out with Re complex:additive:substrate (mol%) = 2:10:100. ^{1}H NMR yields were determined based on the integral ratio of the signals of products and internal standard (mesitylene).

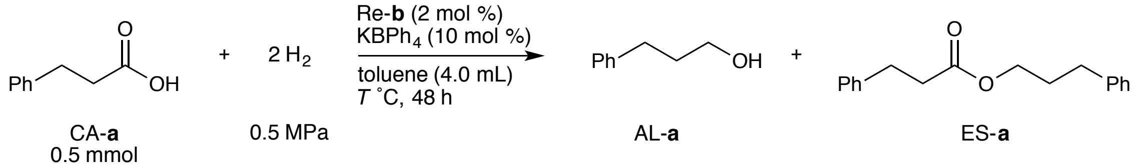

Entry	T (°C)	Yield (%) AL-a	Yield (%) ES-a
1	180	84	7
2	160	61	7
3	150	25	8
4	140	trace	0

Entry	T (°C)	Yield (%) AL-a	Yield (%) ES-a
5	120	0	0
6	100	0	0
7[a]	140	22	6
8[b]	150	79	6

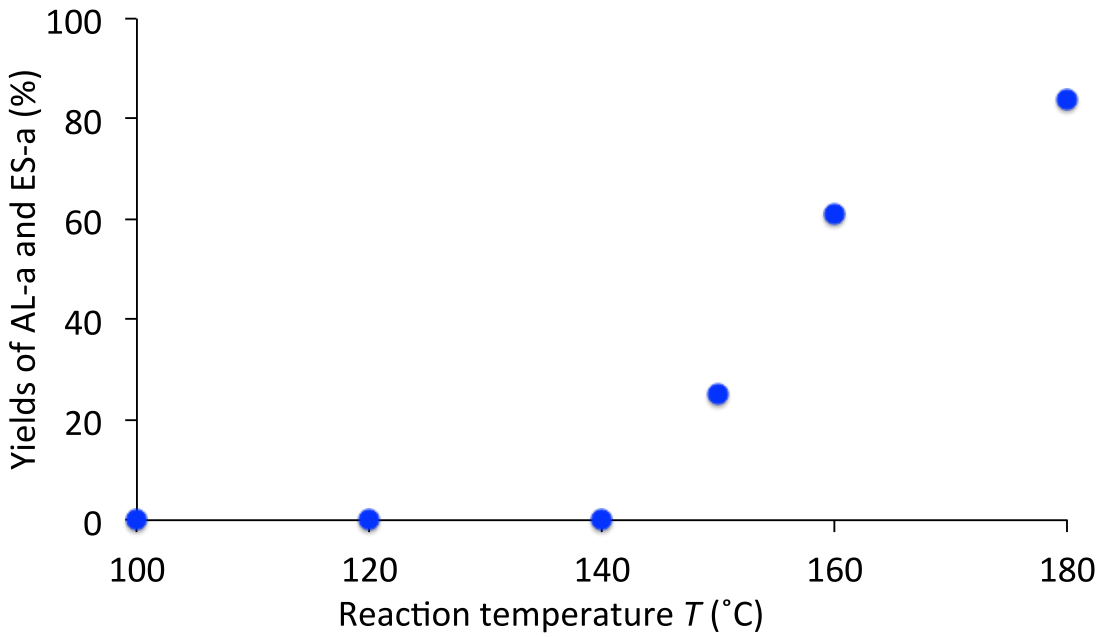

Supplementary Table 6 | Reaction temperature (T) dependency on hydrogenation rate using Re-**b** (P_{H2} = 0.5 MPa; t = 48 h). Unless otherwise specified, the reactions were carried out with Re-**b**:$KBPh_4$:CA-**a** (mol%) = 2:10:100. Blue circle represents the yield of AL-**a** (●). The yield of side product (ES-**a)** was less than 8%. The yields of AL-**a** and ES-**a** were determined by ^{1}H NMR analysis based on internal standard (mesitylene). [a]Reaction time was 72 h. [b]Reaction time was 96 h.

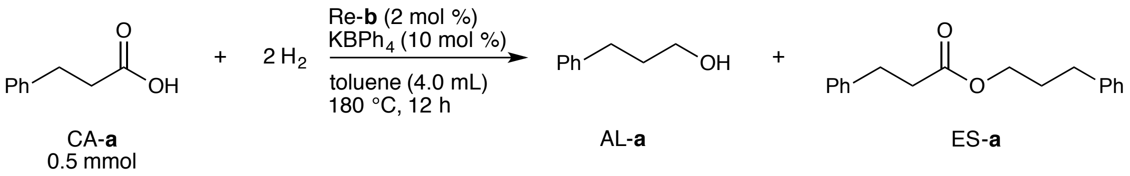

Entry	P_{H2} (MPa)	Yield (%) AL-**a**	Yield (%) ES-**a**
1	4	>99	<1
2	2	>99	0
3	1.5	95	<5
4	1	90	<6
5	0.5	28	4
6[a]	0.1	0	0
7[a]	5	84	7

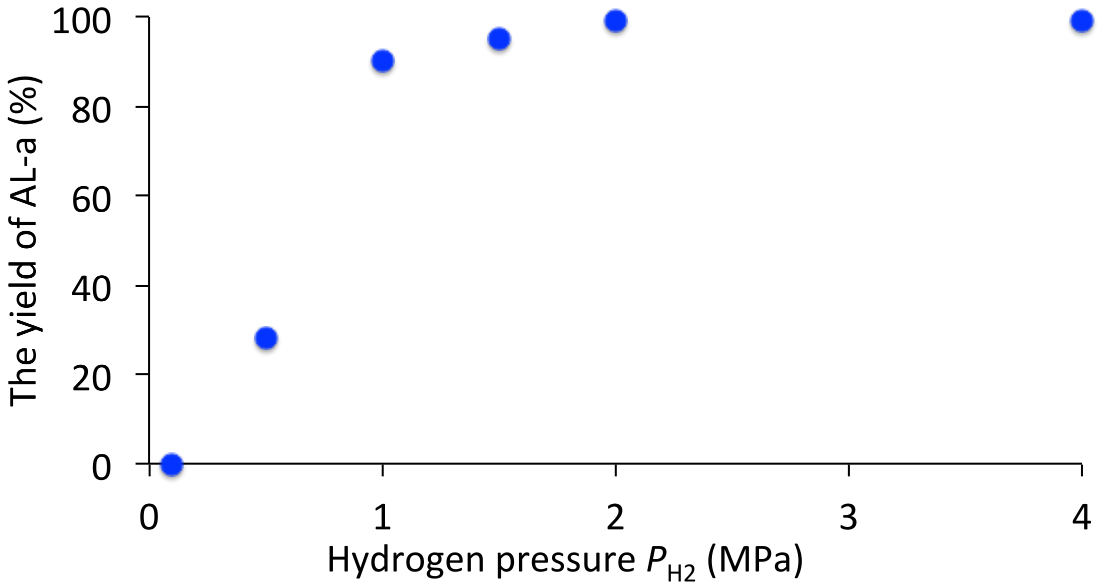

Supplementary Table 7 | Hydrogen pressure (P_{H2}) dependency on hydrogenation rate using Re-**b** (T = 180 ˚C; t = 12 h). Unless otherwise specified, the reactions were carried out with Re-**b**:$KBPh_4$:CA-**a** (mol%) = 2:10:100. Blue circle represents the yield of AL-**a** (●). The yield of side product (ES-**a)** was less than 7%. The yields of AL-**a** and ES-**a** were determined by ^{1}H NMR analysis based on internal standard (mesitylene). [a]Reaction time was 48 h.

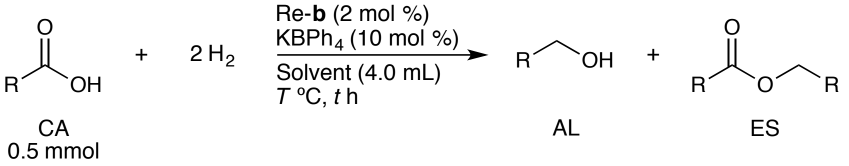

Entry	Carboxylic acid (CA)	P_{H2} MPa, T °C, t h Solvent	Product alcohol (AL)	Yield (%) AL (ester (ES))
1	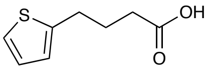 CA-**u**	H_2 4 MPa 160 °C, 36 h toluene	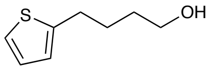 AL-**u**	99 (trace), 83[a]
2	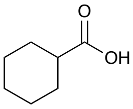 CA-**v**	H_2 4 MPa 160 °C, 36 h THF	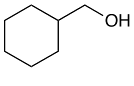 AL-**v**	85 (0)
3	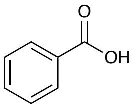 CA-**w**	H_2 4 MPa 160 °C, 40 h toluene	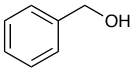 AL-**w**	95 (0)
4	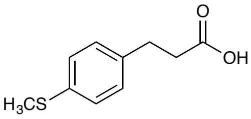 CA-**x**	H_2 1 MPa 180 °C, 12 h toluene	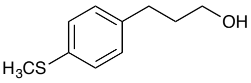 AL-**x**	>95 (3)
5	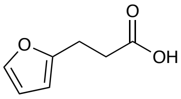 CA-**y**	H_2 6 MPa 160 °C, 48 h THF	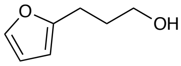 AL-**y**	91 (0)
6	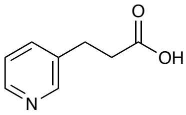 CA-**z**	H_2 4 MPa 180 °C, 48 h toluene	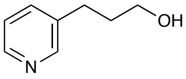 AL-**z**	90 (5)
7	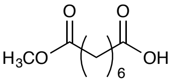 CA-**1**	H_2 4 MPa 160 °C, 24 h THF	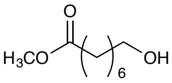 AL-**1**	>95 (trace)
8	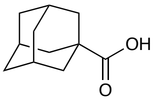 CA-**2**	H_2 4 MPa 160 °C, 24 h toluene	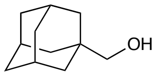 AL-**2**	66 (0)
9	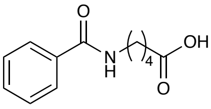 CA-**3**	H_2 4 MPa 160 °C, 36 h toluene	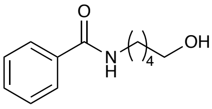 AL-**3**	64 (7)

Entry	Carboxylic acid (CA)	P_{H2} MPa, T °C, t h Solvent	Product alcohol (AL)	Yield (%) AL (ester (ES))
10	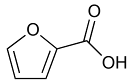 CA-**4**	H_2 8 MPa 160 °C, 48 h THF	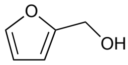 AL-**4**	50 (1)
11	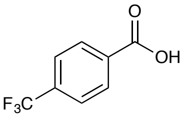 CA-**5**	H_2 4 MPa 180 °C, 12 h toluene	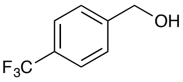 AL-**5**	93 (0)
12	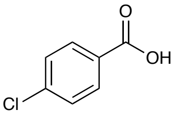 CA-**6**	H_2 4 MPa 180 °C, 12 h toluene	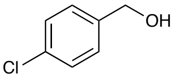 AL-**6**	86 (0)
13	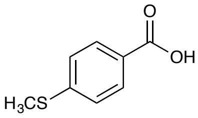 CA-**7**	H_2 6 MPa 180 °C, 48 h toluene	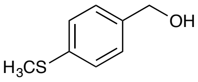 AL-**7**	94 (2)
14	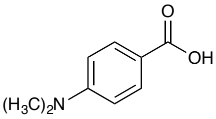 CA-**8**	H_2 4 MPa 180 °C, 12 h toluene	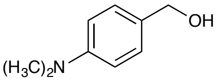 AL-**8**	24 (11)
15	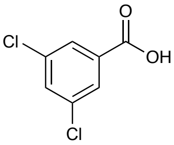 CA-**9**	H_2 4 MPa 160 °C, 36 h THF	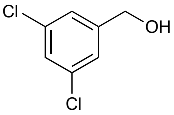 AL-**9**	86 (0)
16	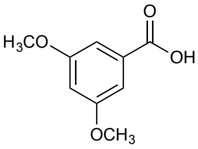 CA-**10**	H_2 4 MPa 180 °C, 12 h THF	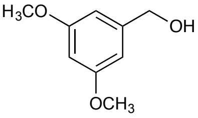 AL-**10**	60 (1)

Supplementary Table 8 | Substrate screening using Re-**b** as catalyst precursor. Unless otherwise specified, the reactions were carried out with Re complex:additive:CA (mol%) = 2:10:100. ^{1}H NMR yields were determined based on the integral ratio of the signals of products and internal standard (mesitylene). ^{a}Isolated yield of alcohol.

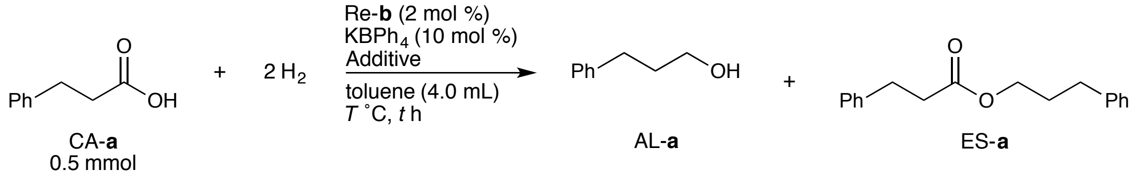

Entry	Additive (mol %)	P_{H2} (MPa) *T* (ºC), *t* (h)	Yield (%)	
			AL-**a**	ES-**a**
1	TH-**a** (60 mol %)	H_2 0.5 MPa, 180 °C, 48 h	88	<7
2	TH-**b** (60 mol %)	H_2 4 MPa, 160 °C, 24 h	74	5
3	TH-**b** (30 mol %)	H_2 4 MPa, 160 °C, 24 h	>99	trace
4	TH-**c** (10 mol %)	H_2 0.5 MPa, 180 °C, 48 h	87	6
5	TH-**c** (60 mol %)	H_2 4 MPa, 160 °C, 24 h	94	1
6	TH-**d** (30 mol %)	H_2 4 MPa, 160 °C, 24 h	>99	<2
7	TH-**e** (30 mol %)	H_2 4 MPa, 160 °C, 24 h	>99	<1
8	TH-**e** (30 mol %)	H_2 0.5 MPa, 180 °C, 48 h	67	12
9	TH-**a** (10 mol %) TH-**b** (10 mol %) TH-**c** (10 mol %)	H_2 4 MPa, 160 °C, 24 h	98	<2

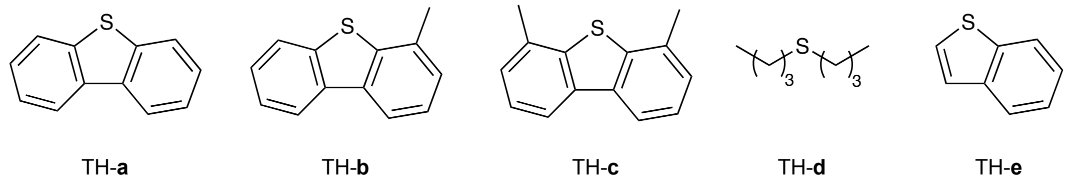

Supplementary Table 9 | Hydrogenation of CA-**a** using Re-**b** in the presence of sulfur-containing substances. Unless otherwise specified, the reactions were carried out with Re complex:$KBPh_4$:CA-**a** (mol%) = 2:10:100. ^{1}H NMR yields were determined based on the integral ratio of the signals of products and internal standard (mesitylene).

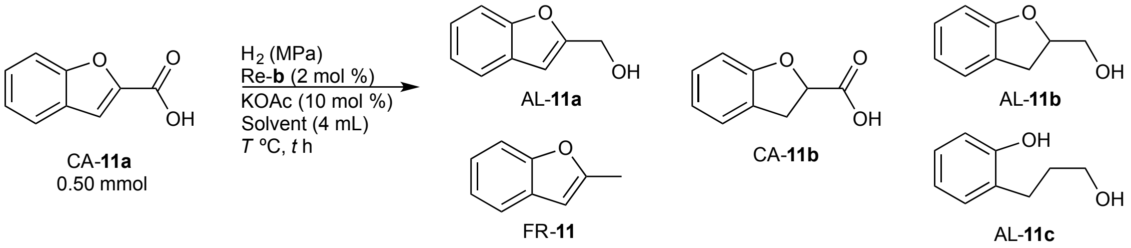

Entry	Solvent	P_{H2} (MPa)	T (°C)	t (h)	Yield (%) AL-**11a**	FR-**11**	CA-**11b**	AL-**11b**	AL-**11c**
1	THF	4	160	16	42	–	–	20	–
2	$PhCF_3$	4	160	16	trace	–	–	74	6
3	toluene	4	160	16	7	–	–	64	–
4	THF	4	140	16	33	4	–	20	–
5	1,4-dioxane	4	140	16	21	6	–	9	–
6	CPME	4	140	16	8	20	–	30	–
7	3-methyl-3-pentanol	4	140	16	20	–	–	12	–
8	THF	2	160	16	47	5	–	16	–
9	THF	2	160	60	<5	37	–	<5	–
10	$PhCF_3$	4	100	48	–	–	80	6	-
11[a]	$PhCF_3$	6	160	10	–	–	–	84	10
12[a]	$PhCF_3$	6	160	168	–	–	–	20	78

Supplementary Table 10 | Hydrogenation of CA-**11a** using Re-**b**. Unless otherwise specified, the reactions were carried out with Re complex:additive:CA-**11a** (mol%) = 2:10:100. ^{1}H NMR yields were determined based on the integral ratio of the signals of products and internal standard (mesitylene). [a]4 mol% Re-**b** and 20 mol% KOAc were used.

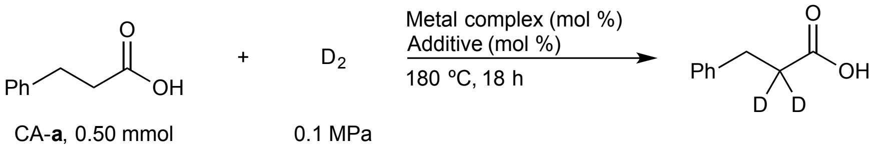

Entry	Metal complex (mol %)	Additive (mol %)	D content (%)
1	–	–	0
2	Re-**b** (2 mol %)	–	0
3	–	$KBPh_4$ (10 mol %)	5
4	–	KOAc (10 mol %)	0
5[a]	Re-**b** (2 mol %)	$KBPh_4$ (10 mol %)	98
6[a, b]	Re-**b** (0.4 mol %)	$KBPh_4$ (2 mol %)	80
7	Ru-dppb complex (2 mol %)	Na(acac) (10 mol %)	0

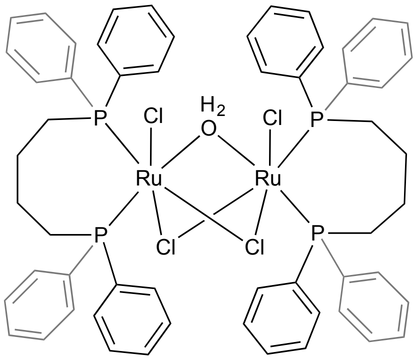

Ru-dppb-complex

Supplementary Table 11 | Deuteration of α-C–H in CA-**a**. Unless otherwise specified, the reactions were carried out under the conditions (180 ºC, 18 h) shown in the above scheme. D content was determined by ^{1}H NMR based on the integral ratio of the signals of products and internal standard (mesitylene). [a]Reaction time was 24 h. [b]2.5 mmol of CA-**a** was used as substrate.

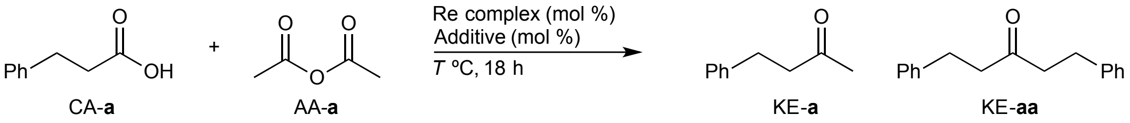

Entry	CA-**a** (mmol)	AA-**a** (mmol)	Re complex (mol %)	Additive (mol %)	*T* (°C)	Yield (%) KE-**a**	KE-**aa**
1	1.0	1.0	–	–	180	0	0
2	1.0	1.0	Re-**b** (2 mol %)	$KBPh_4$ (10 mol %)	180	0	trace
3	1.0	1.0	Re-**b** (2 mol %)	KOAc (10 mol %)	180	24	21
4	0.5	1.0	Re-**b** (2 mol %)	KOAc (10 mol %)	180	36	20
5	1.0	0.5	Re-**b** (2 mol %)	KOAc (10 mol %)	180	13	20
6	0.5	2.0	Re-**b** (2 mol %)	KOAc (10 mol %)	180	58	12
7	0.5	5.0	Re-**b** (2 mol %)	KOAc (10 mol %)	180	36[a]	trace
8	0.5	5.0	Re-**b** (2 mol %)	KOAc (10 mol %)	160	20	trace
9	0.5	5.0	Re-**b** (2 mol %)	KOAc (10 mol %)	140	3	trace
10	0.5	5.0	Re-**b** (4 mol %)	KOAc (20 mol %)	160	36	trace
11	0.5	5.0	Re-**b** (6 mol %)	KOAc (30 mol %)	160	50	trace
12	0.5	5.0	–	KOAc (30 mol %)	160	24	trace
13	0.5	5.0	Re-**j** (2 mol %)	KOAc (10 mol %)	180	28	trace

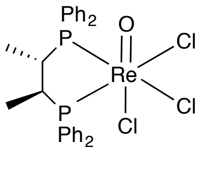

Re-**b**

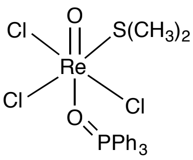

Re-**j**

Supplementary Table 12 | Optimization of reaction conditions in unsymmetrical ketones (KEs) synthesis using CA-**a** and AA-**a**. Unless otherwise specified, the reactions were carried out under the conditions shown in the above scheme. The yields of products were determined by ^{1}H NMR based on the integral ratio of the signals of products and internal standard (1,1,2,2-tetrachloroethane). [a] The average of two trials.

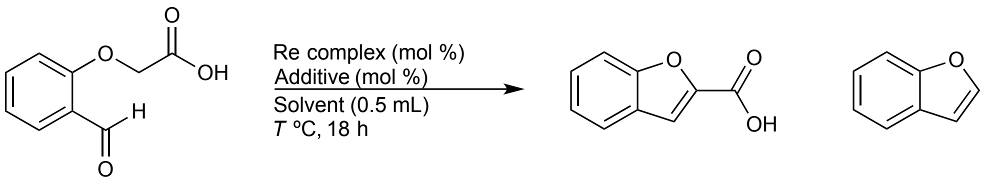

CA-**12**, 0.50 mmol   CA-**11a**   FR-**12**

Entry	Re complex (mol %)	Additive (mol %)	Solvent	*T* (ºC)	Yield CA-**11a**	Yield FR-**12**
1	Re-**a** (6 mol %)	KOAc (30 mol %)	mesitylene	180	50	28
2	Re-**b** (6 mol %)	KOAc (30 mol %)	mesitylene	180	48	30
3	Re-**h** (6 mol %)	KOAc (30 mol %)	mesitylene	180	0	58
4	Re-**j** (6 mol %)	KOAc (30 mol %)	mesitylene	180	62	22
5	Re-**k** (6 mol %)	KOAc (30 mol %)	mesitylene	180	32	44
6	Re-**m** (6 mol %)	KOAc (30 mol %)	mesitylene	180	44	30
7	Re-**j** (6 mol %)	KOAc (30 mol %)	mesitylene	160	60	20
8	Re-**j** (6 mol %)	KOAc (30 mol %)	mesitylene	140	52	19
9	Re-**j** (6 mol %)	KOAc (30 mol %)	mesitylene	120	16	6
10	Re-**j** (6 mol %)	KOAc (30 mol %)	mesitylene	100	0	0
11	Re-**j** (6 mol %)	KOAc (30 mol %)	neat	140	52	11
12	Re-**j** (6 mol %)	KOAc (30 mol %)	toluene	140	50	20
13	Re-**j** (6 mol %)	KOAc (30 mol %)	4-methyl anisole	140	48	28
14	Re-**j** (6 mol %)	KOAc (30 mol %)	AcOBu	140	48	24
15	Re-**j** (6 mol %)	KOAc (30 mol %)	tetraglyme	140	40	14
16	Re-**j** (6 mol %)	KOAc (30 mol %)	CPME	140	50	30
17	Re-**j** (6 mol %)	KOAc (30 mol %)	3-methyl-3-pentanol	140	48	20

Entry	Re complex (mol %)	Additive (mol %)	Solvent	*T* (ºC)	Yield CA-**11a**	FR-**12**
18	Re-**j** (6 mol %)	KOAc (30 mol %)	$PhCF_3$	140	70	18
19	Re-**j** (6 mol %)	KOAc (30 mol %)	C_6F_6	140	52	15
20	Re-**j** (6 mol %)	KOAc (30 mol %)	$C_6F_5(CF_3)$	140	56	22
21	Re-**j** (6 mol %)	KOAc (30 mol %)	*m*-$(CF_3)_2C_6H_4$	140	56	22
22	Re-**j** (6 mol %)	KOAc (30 mol %)	*p*-$(CF_3)_2C_6H_4$	140	58	20
23	Re-**j** (6 mol %)	CF_3CO_2K (30 mol %)	$PhCF_3$	140	22	13
24	Re-**j** (6 mol %)	$PhCO_2K$ (30 mol %)	$PhCF_3$	140	<70	26
25	Re-**j** (6 mol %)	$(CH_3)_3CCO_2K$ (30 mol %)	$PhCF_3$	140	60	22
26	Re-**j** (6 mol %)	KOAc (18 mol %)	$PhCF_3$	140	58	18
27	Re-**j** (6 mol %)	KOAc (12 mol %)	$PhCF_3$	140	26	15
28	Re-**b** (6 mol %)	KOAc (30 mol %)	$PhCF_3$	140	33	24
29	–	KOAc (30 mol %)	$PhCF_3$	140	5	22
30	Re-**b** (6 mol %)	–	$PhCF_3$	140	9	9
31	Re-**j** (6 mol %)	–	$PhCF_3$	140	4	2

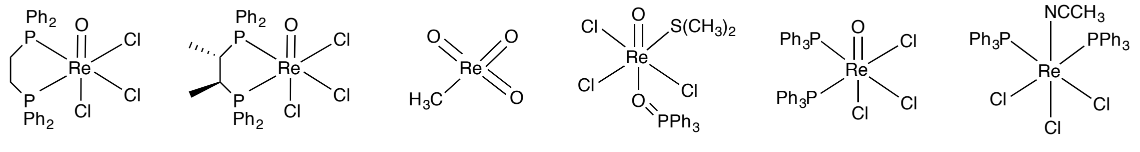

Supplementary Table 13 | Optimization of reaction conditions in intramolecular aldol cyclization of CA-**12**. Unless otherwise specified, the reactions were carried out under the conditions shown in the above scheme. The yields of products were determined by ^{1}H NMR based on the integral ratio of the signals of products and internal standard (1,1,2,2-tetrachloroethane).

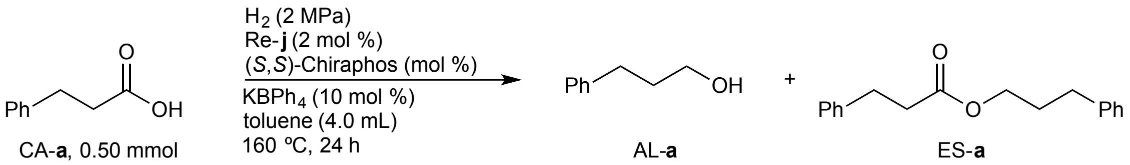

Entry	(*S*,*S*)-Chiraphos (mol %)	Color of reaction mixture	^{1}H NMR yield (%)[a] AL-**a**	ES-**a**
1	2 mol %	Dark brown	93%	3%
2	4 mol %	yellow	22%	7%
3	6 mol %	yellow	0%	0%

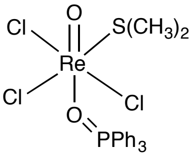

Re-**j**

Supplementary Table 14 | Investigation of the effect of Re to chiraphos ratio on the yields of products. Unless otherwise specified, the reactions were carried out under the conditions shown in the above scheme. The yields of products were determined by ^{1}H NMR based on the integral ratio of the signals of products and internal standard (1,1,2,2-tetrachloroethane).

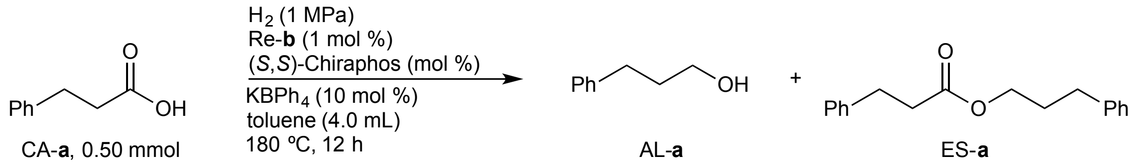

Entry	(*S*,*S*)-Chiraphos (mol %)	Color of reaction mixture	^{1}H NMR yield (%) AL-**a**	ES-**a**
1	0 mol %	Dark brown	49%	7%
2	1 mol %	yellow	10%	4%

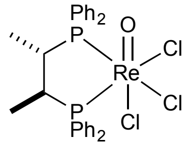

Re-**b**

Supplementary Table 15 | Investigation of the effects of additional chiraphos on the yields of products. Unless otherwise specified, the reactions were carried out under the conditions shown in the above scheme. The yields of products were determined by ^{1}H NMR based on the integral ratio of the signals of products and internal standard (1,1,2,2-tetrachloroethane).

3. Supplementary Figures

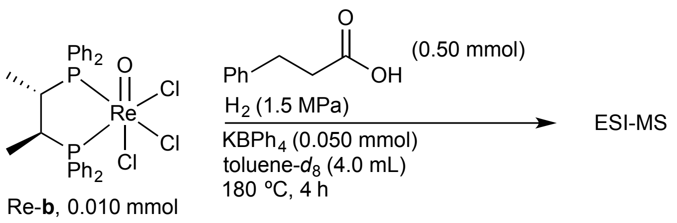

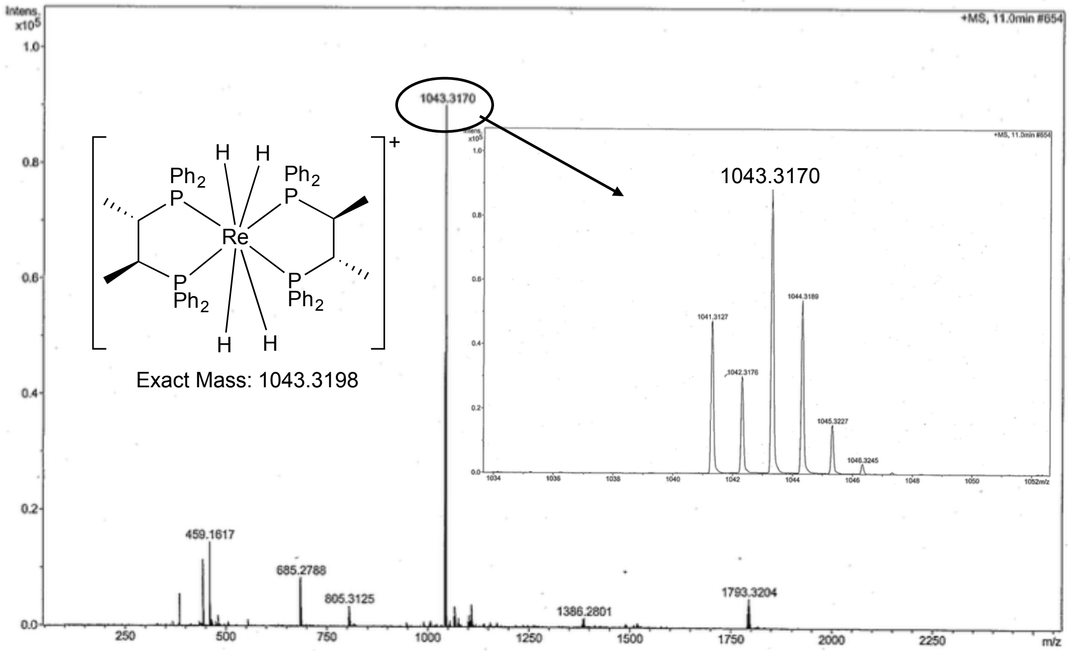

Supplementary Figure 1 | ESI-MS spectrum of a mixture of Re species generated upon hydrogenation of CA-**a** using Re-**b** and $KBPh_4$ ($[Re]_0$ = 2.5 mM, $[CA\text{-}\mathbf{a}]_0$ = 125 mM, $[KBPh_4]_0$ = 12.5 mM, P_{H2} = 1.5 MPa, T = 180 °C, and t = 4 h). The separation pattern of the signals involving the top peak (*m/z* 1043.3170) was magnified (inset).

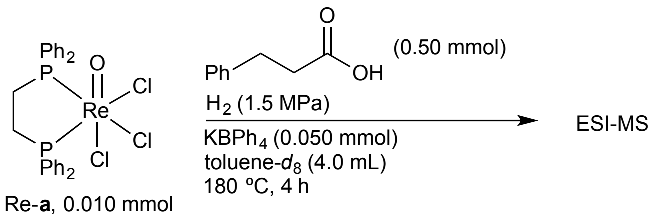

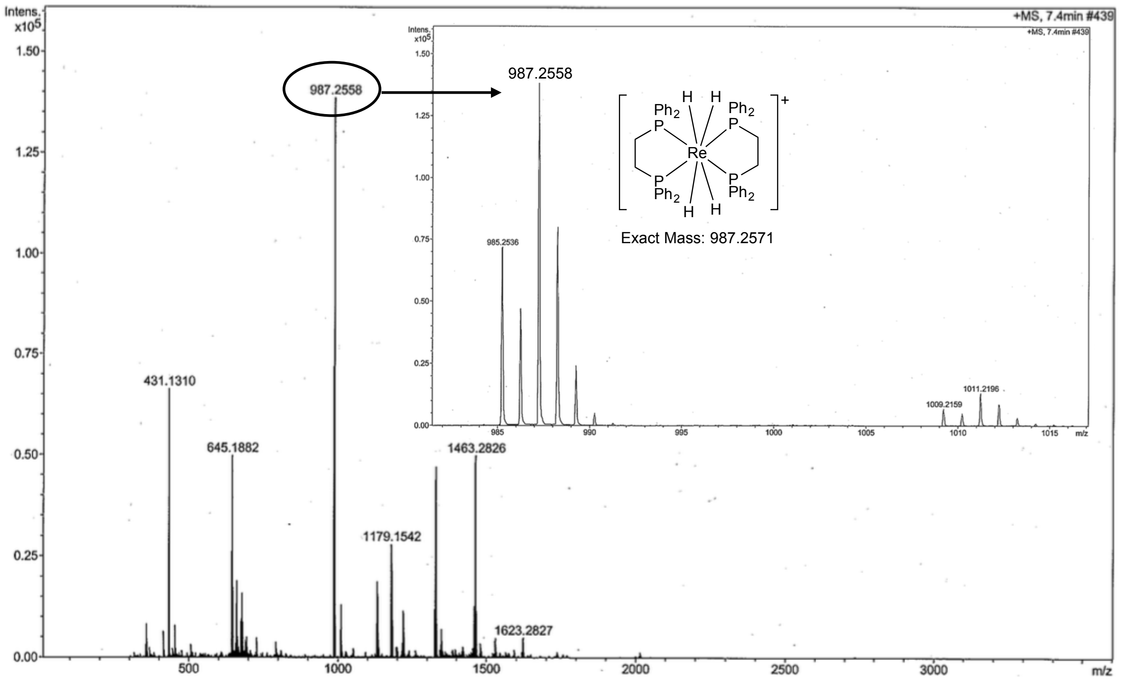

Supplementary Figure 2 | ESI-MS spectrum of a mixture of Re species generated upon hydrogenation of CA-**a** using Re-**a** and $KBPh_4$ at P_{H2} = 1.5 MPa ($[Re]_0$ = 2.5 mM, $[CA\text{-}\mathbf{a}]_0$ = 125 mM, $[KBPh_4]_0$ = 12.5 mM, T = 180 °C, and t = 4 h). The separation patterns of the signals involving the top peak (m/z 987.2558) were magnified (inset).

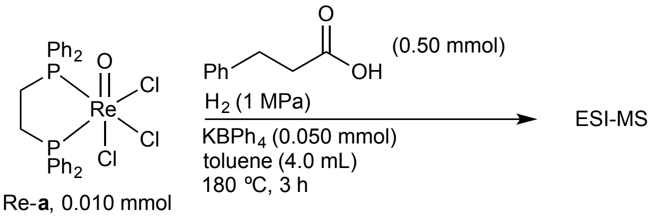

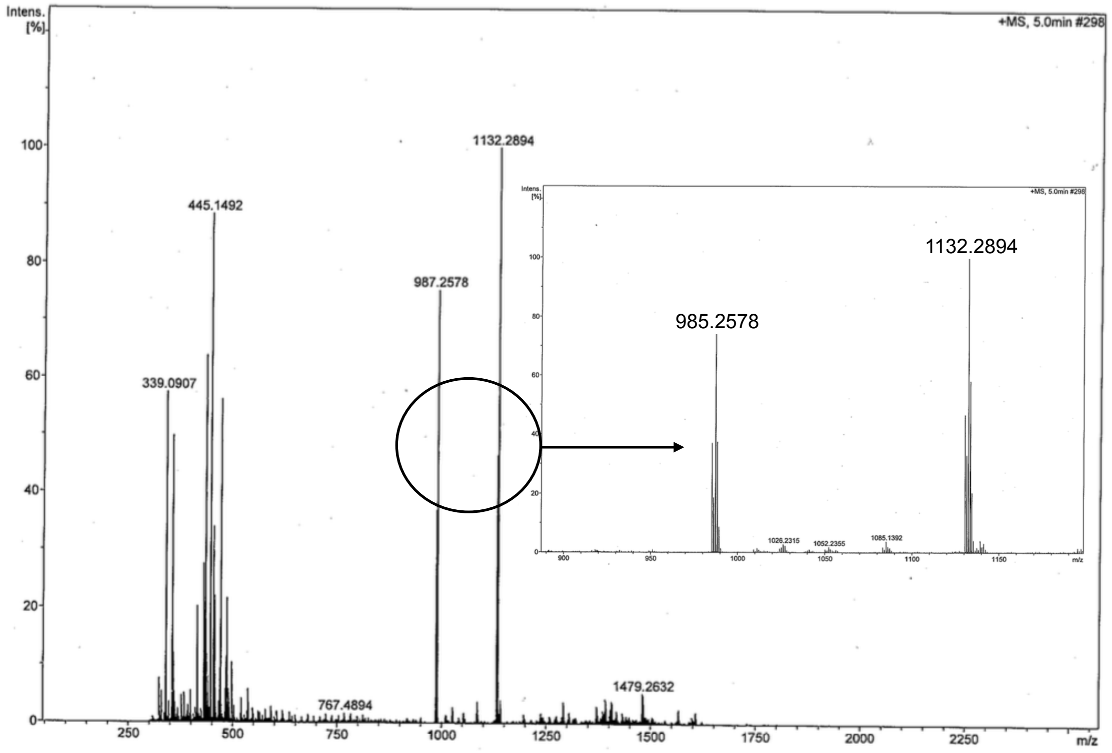

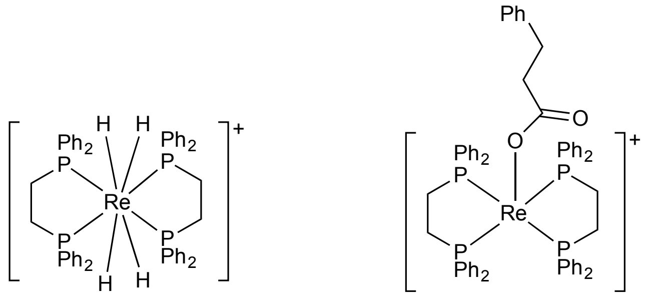

Exact Mass: 987.2571

Exact Mass: 1132.2861

Supplementary Figure 3 | ESI-MS spectrum of a mixture of Re species generated upon hydrogenation of CA-**a** using Re-**a** and $KBPh_4$ at P_{H2} = 1 MPa ($[Re]_0$ = 2.5 mM, [CA-**a**]$_0$ = 125 mM, $[KBPh_4]_0$ = 12.5 mM, T = 180 °C, and t = 3 h). The separation patterns of the signals involving the top peak (*m*/*z* 985.2578 and 1132.2894) were magnified (inset).

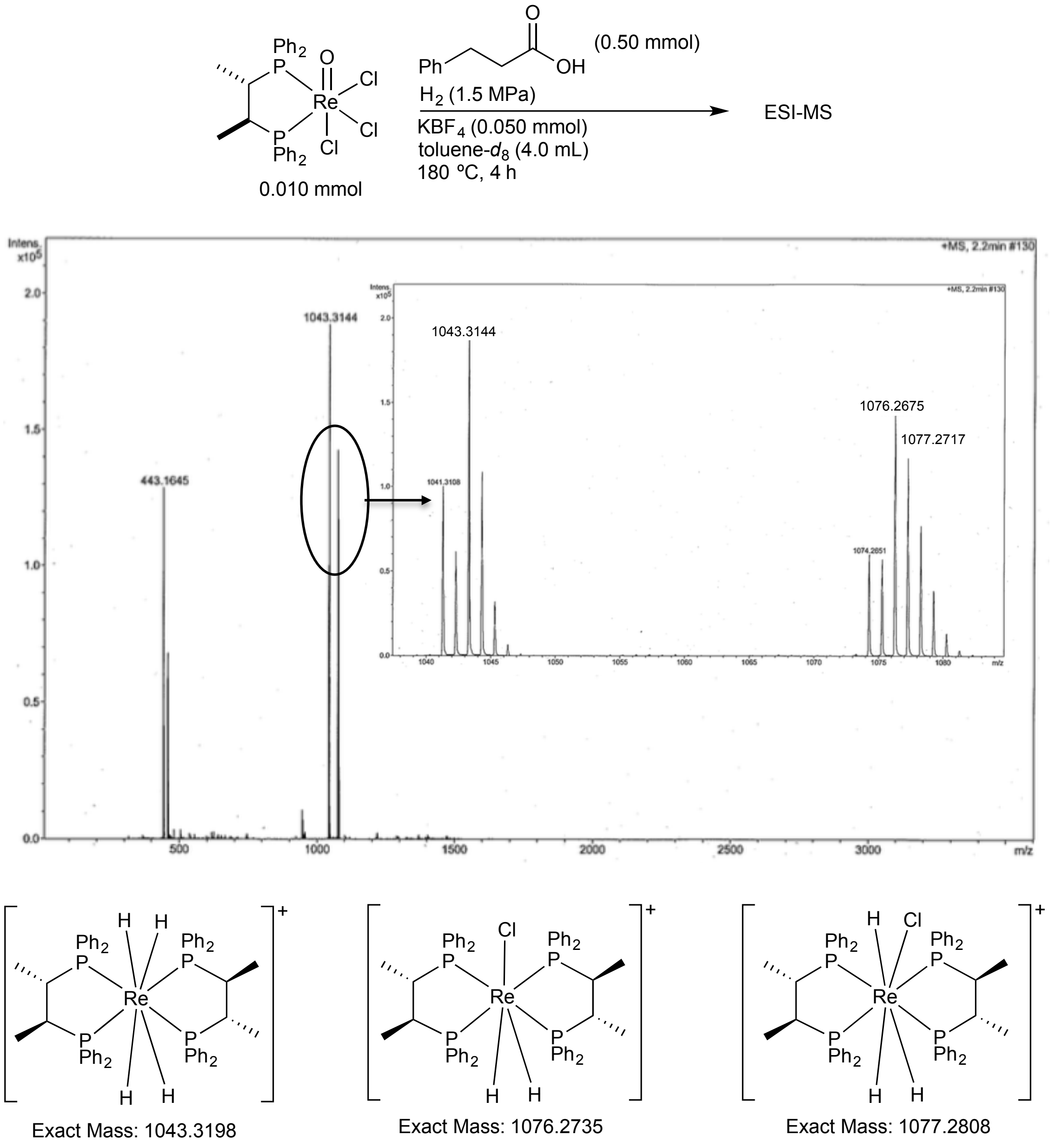

Supplementary Figure 4 | ESI-MS spectrum of a mixture of Re species generated upon hydrogenation of CA-**a** using Re-**b** and KBF_4 ($[Re]_0$ = 2.5 mM, $[CA\text{-}\mathbf{a}]_0$ = 125 mM, $[KBF_4]_0$ = 12.5 mM, P_{H2} = 1.5 MPa, T = 180 °C, and t = 4 h). The separation patterns of the signals involving the top peak (*m*/*z* 1043.3144 and 1076.2675) were magnified (inset).

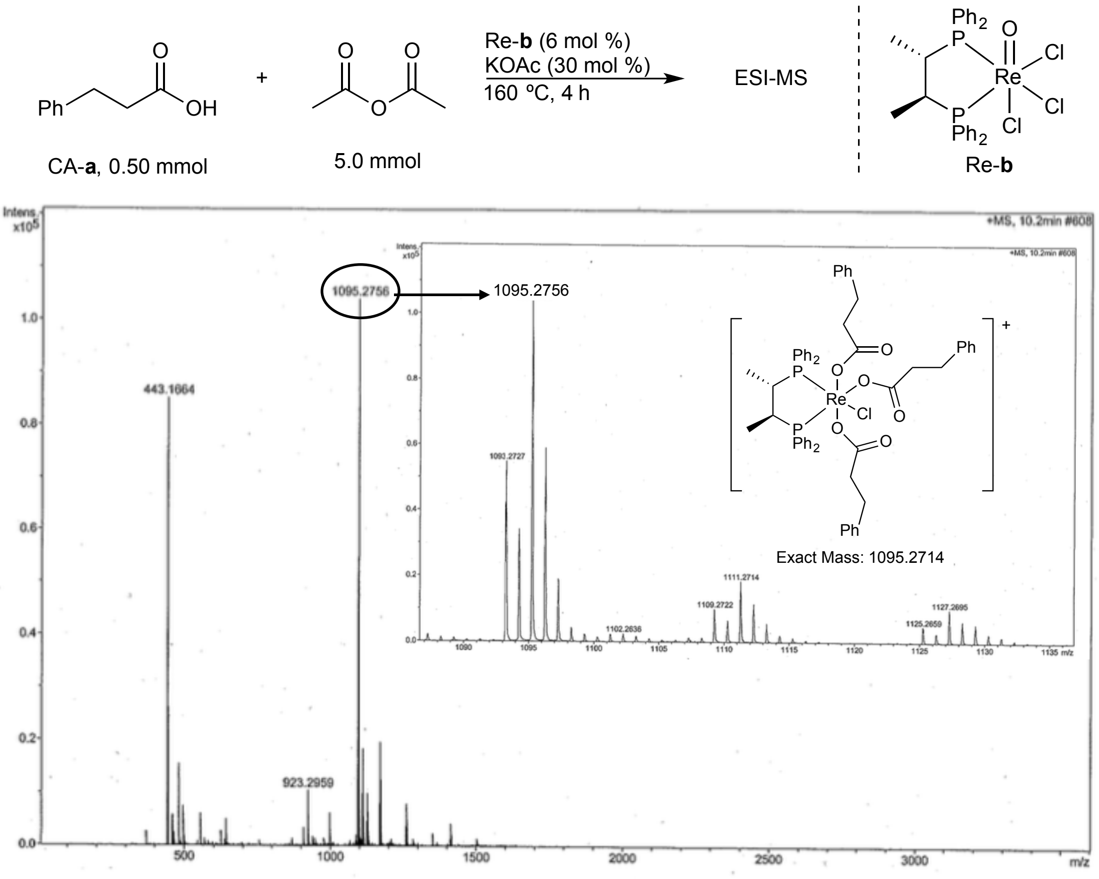

Supplementary Figure 5 | ESI-MS spectrum of a mixture of Re species generated upon synthesis of unsymmetrical ketone(s) from CA-**a** using Re-**b** in excess Ac_2O (160 °C, 4 h). Unless otherwise specified, the reactions were carried out in above conditions. The separation patterns of the signals involving the top peak (*m*/*z* 1095.2756) were magnified (inset).

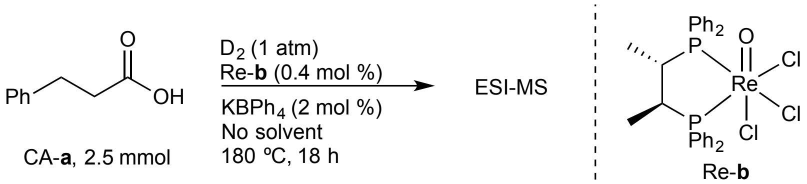

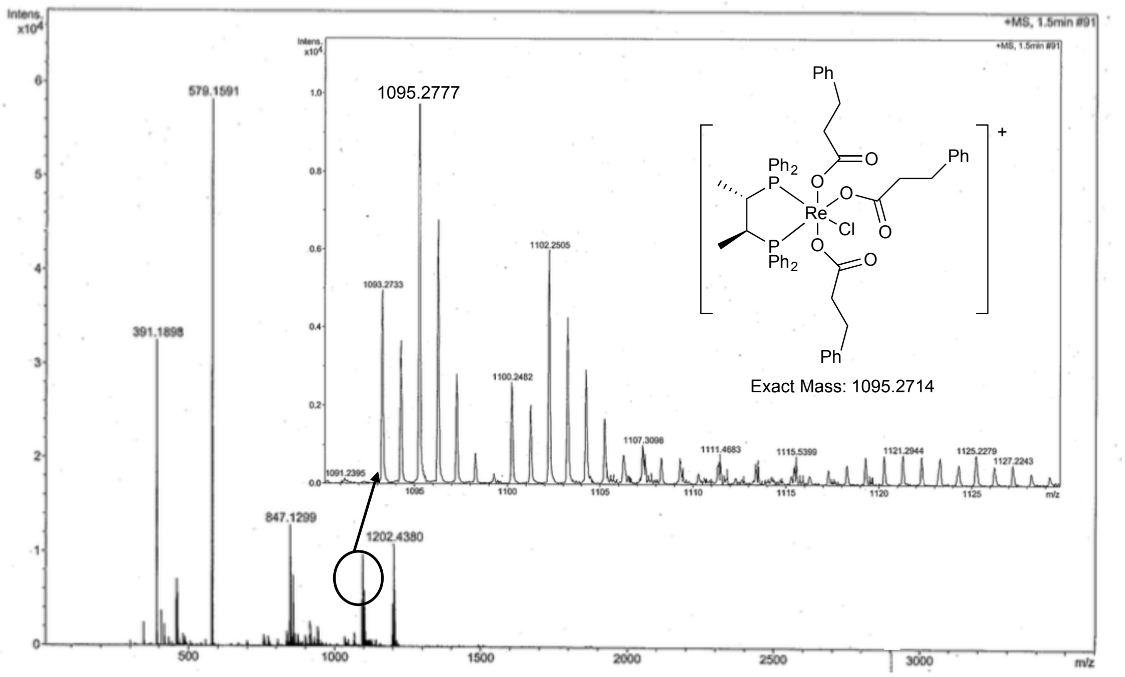

Supplementary Figure 6 | ESI-MS spectrum of a mixture of Re species generated upon deuteration reaction using Re-**b** without solvent (P_{D2} = 1 atm (0.1 MPa), 180 °C, 18 h). The separation patterns of the signals involving the top peak (*m*/*z* 1095.2777) were magnified (inset).

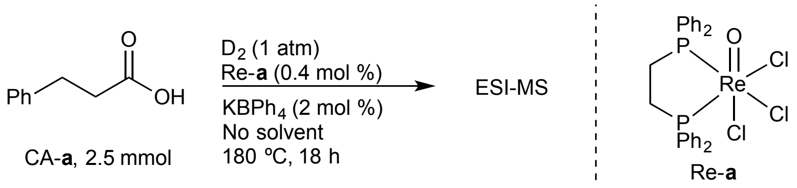

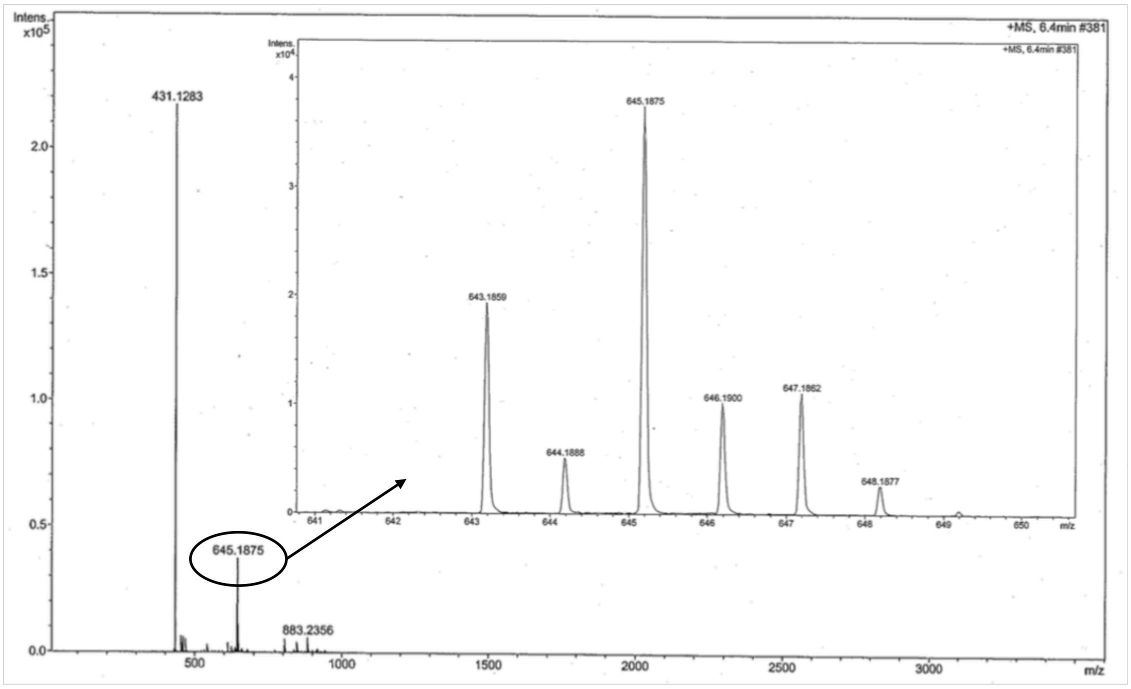

Supplementary Figure 7 | ESI-MS spectrum of a mixture of Re species generated upon deuteration using Re-**a** without solvent (P_{D2} = 1 atm (0.1 MPa), 180 °C, 18 h). The separation pattern of the signals involving the top peak (*m*/*z* 645.1875) was magnified (inset), which suggest some decomposed Re species that cannot be identified.

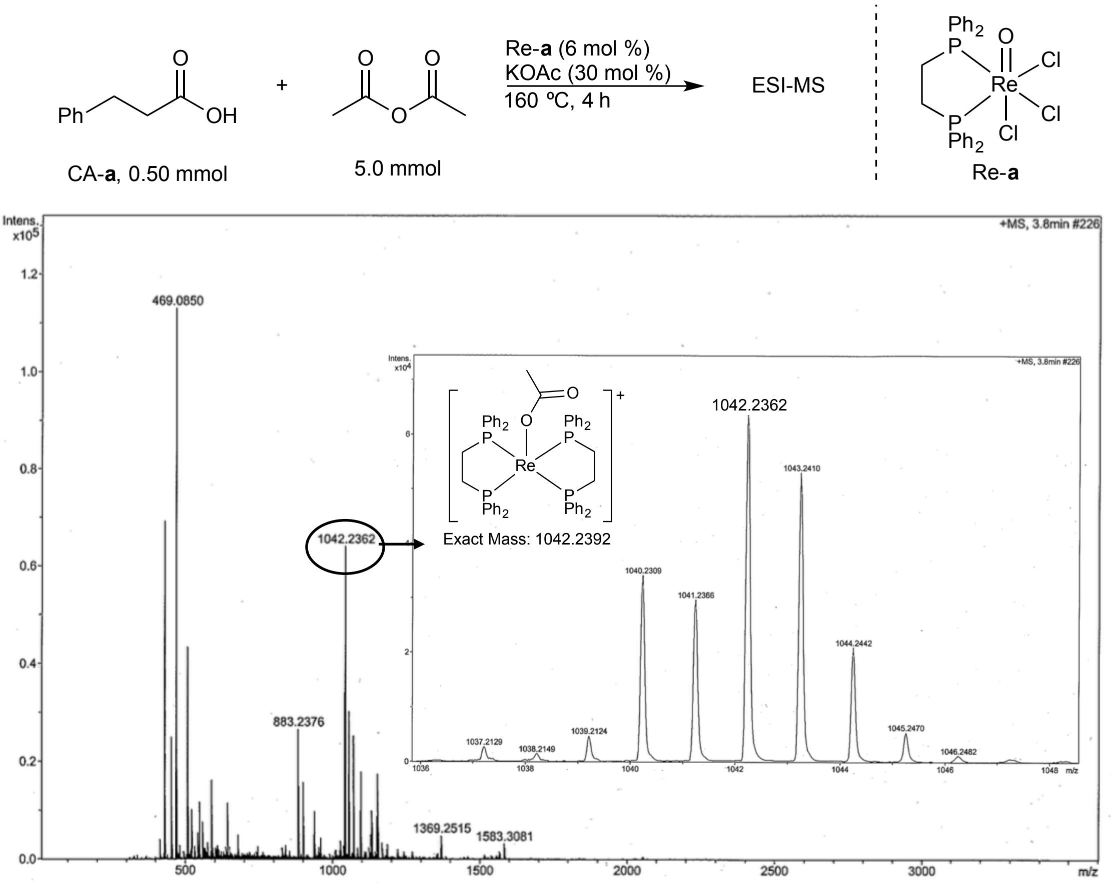

Supplementary Figure 8 | ESI-MS spectrum of a mixture of Re species generated upon synthesis of unsymmetrical ketone(s) from CA-**a** using Re-**a** in excess Ac_2O. The separation pattern of the signals involving the top peak (*m*/*z* 1042.2362) was magnified (inset).

4. Experimental procedures

4.1. Hydrogenation experiments

Representative procedure for hydrogenation of carboxylic acid using rhenium complexes: Re complex = $ReOCl_3$[(*S*,*S*)-Chiraphos] (Re-b) : (Supplementary Table 1, entry 15)

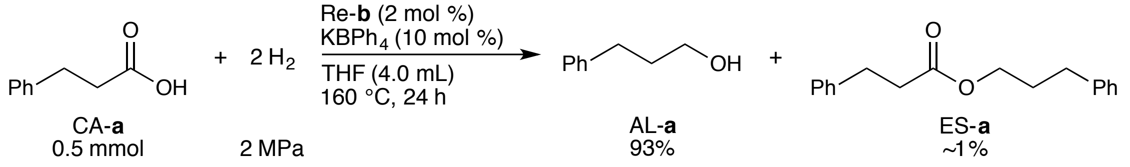

3-Phenylpropionic acid (CA-**a**) (0.5 mmol, 75.0 mg), $KBPh_4$ (0.05 mmol, 17.9 mg), $ReOCl_3$[(*S*,*S*)-Chiraphos] (Re-**b**) (0.01 mmol, 7.3 mg) and a magnetic stirrer bar were placed in a dried up glass tube that was inserted in an autoclave, which was purged Ar gas several times. Anhydrous THF (4.0 mL) was added under a continuous flow of Ar, and the autoclave was purged five times with H_2 (1 MPa). The autoclave was pressurized with H_2 (P_{H2} = 2 MPa) at 25 °C and heated at 160 °C, where the mixture was stirred (500 rpm) for 24 h. Then, the autoclave was cooled to 0 °C, before the reaction mixture was transferred to a 100 mL round bottom flask containing $CHCl_3$. The mixture was concentrated (~ 30 mmHg, 40 °C), and the residue was dissolved in $CDCl_3$ and analyzed by ^{1}H NMR spectroscopy. Yields of 3-phenyl-1-propanol (AL-**a**) (93%) and 1-(3-phenylpropyl) 3-phenylpropanoate (ES-**a**) (~1%) were calculated based on the integration ratio of their signals relative to the internal standard mesitylene and by GC-MS analysis, respectively.

3-Phenyl-1-propanol (AL-**a, k**),[2] 2-(4-bromophenyl)ethaol (AL-**b**),[3] *n*-nonyl alcohol (AL-**c**) (authentic sample purchased from a chemical company), 3-cyclohexyl-1-propanol (AL-**d**),[4] 4-phenyl-1-butanol (AL-**e**),[5] 2-(4-isobutylphenyl)propan-1-ol (AL-**f**),[6] 1,16-hexadecanediol (AL-**g**),[7] 2-phenoxyethanol (AL-**h**),[8] 2-(3-methoxyphenyl)ethanol (AL-**i**),[9] 2-(3,4,5-trimethoxyphenyl)ethanol (AL-**j**),[10] 3-(4-chlorophenyl)propan-1-ol (AL-**l**),[11] 2-methyl-3-phenylpropan-1-ol (AL-**m**),[4] methyl 4-(3-hydroxypropyl)benzoate

(AL-**n**),[12] 2,2-diphenylethanol (AL-**o)**,[9] 4-(1H-indol-3-yl)butan-1-ol (AL-**p**),[13] 2-(4-hydroxybutyl)thiophene (AL-**u**),[14] cyclohexyl-methanol (AL-**v**),[15] benzyl alcohol (AL-**w**),[16] 3-[4-(methylthio)phenyl]propan-1-ol (AL-**x**),[17] 3-(furan-2-yl)propan-1-ol (AL-**y**),[4] 3-pyridinepropanol (AL-**z**),[18] methyl 8-hydroxyoctanate (AL-**1**),[19] 1-adamantanemethanol (AL-**2**),[6] *N*-(5-hydroxypentyl)benzamide (AL-**3**),[20] furan-2-methanol (AL-**4**),[21] [4-(trifluoromethyl)phenyl]methanol (AL-**5**),[16] 4-chlorophenyl-methanol (AL-**6**),[15] 4-methylsulphanylphenyl-methanol (AL-**7**),[15] (4-dimethylamino)phenyl-methanol (AL-**8**),[15] 3,5-dichlorobenzyl alcohol (AL-**9**),[22] (3,5-dimethoxyphenyl)methanol (AL-**10**),[23] benzofuran-2-ylmethanol (AL-**11**),[24] (2,3-dihydrobenzofuran-2-yl)methanol (AL-**11b**),[25] 2-(3-hydroxypropyl)phenol (AL-**11c**),[26] 2,3-dihydrobenzofuran-2-carboxylic acid (CA-**11b**),[27] 2-methylbenzofuran (FR-**11**)[28] and 3-phenylpropan-1-*d*-1-ol (AL-**a**-d_1)[29] are all known compounds.

4.2. Deuteration experiments

Representative procedure for deuteration of *α*-C–H of 3-phenylpropionic acid (CA-a) using rhenium complex : Re complex = $ReOCl_3$[(*S*,*S*)-Chiraphos] (Re-b) : (Fig. 4, a)

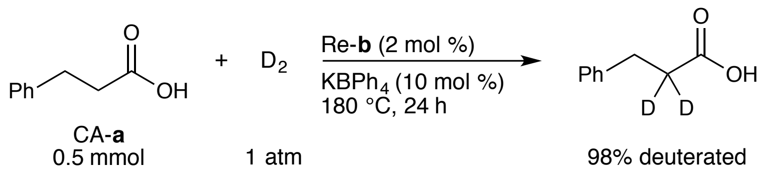

3-Phenylpropionic acid (CA-**a**) (0.5 mmol, 75.0 mg), $KBPh_4$ (0.05 mmol, 17.9 mg), $ReOCl_3$[(*S*,*S*)-Chiraphos] (Re-**b**) (0.01 mmol, 7.3 mg) and a magnetic stirrer bar were placed in a dried up 75 mL J. Young tube equipped with a three ways cock and a rubber balloon, which was purged Ar gas several times and filled with D_2 (P_{D2} = 1 atm). The J. Young tube was heated at 180 °C, where the mixture was stirred (500 rpm) for 24 h and cooled to 0 °C. The residue was dissolved in $CDCl_3$ and analyzed by ^{1}H NMR spectroscopy. The deuteration content at α-C–H of 3-phenylpropionic acid (CA-**a**) (98%) was calculated based on the integration ratio of their signals relative to the internal standard 1,1,2,2-tetrachloroethane.

4.3. Unsymmetrical ketones synthesis

Representative procedure for unsymmetrical ketones synthesis using rhenium complex : Re complex = $ReOCl_3$[(*S*,*S*)-Chiraphos] (Re-b) : (Fig. 4, b)

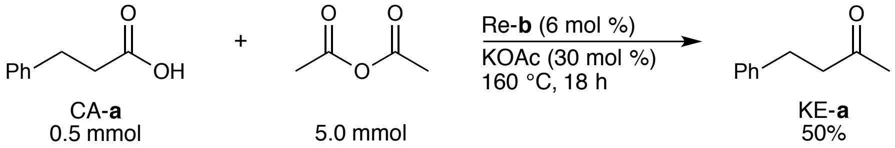

3-Phenylpropionic acid (CA-**a**) (0.5 mmol, 75.0 mg), KOAc (0.15 mmol, 14.7 mg), $ReOCl_3$[(*S*,*S*)-Chiraphos] (Re-**b**) (0.03 mmol, 22.0 mg) and a magnetic stirrer bar were placed in a dried up 75 mL J. Young tube, which was purged Ar gas several times. Acetic anhydride (5.0 mmol, 470 μL) was added under a continuous flow of Ar. The J. Young tube was heated at 160 °C, where the mixture was stirred (500 rpm) for 18 h and cooled to 0 °C. The residue was dissolved in $CDCl_3$ and analyzed by ^{1}H NMR spectroscopy. The yield of 4-phenylbutan-2-one (KE-**a**) (50%) was calculated based on the integration ratio of their signals relative to the internal standard 1,1,2,2-tetrachloroethane.

The structures of obtained ketones (KE-**a**–**c**) were double confirmed after isolation, and the spectral data were almost consistent with the values reported.[30–32]

KE-**a** and -**b** were isolated by the extraction method reported[33] and silica gel chromatography (eluent; hexane/AcOEt = 25/1 for KE-**a**, and 50/1 for KE-**b**, respectively). KE-**c** was isolated by silica gel chromatography (eluent; hexane/AcOEt = 50:1).

4-Phenylbutan-2-one (KE-**a**),[30] colorless liquid: ^{1}H NMR (600 MHz, $CDCl_3$) δ 2.14 (s, 3H), 2.76 (t, J = 7.6 Hz, 2H), 2.90 (t, J = 7.6 Hz, 2H), 7.16–7.21 (m, 3H), 7.25–7.30 (m, 2H) ppm.; $^{13}C\{^1H\}$ NMR (150 MHz, $CDCl_3$) δ 29.7, 30.0, 45.1, 126.1, 128.3, 128.5, 141.0, 207.9 ppm.

1-Phenylpentan-3-one (KE-**b**),[31] colorless liquid: ^{1}H NMR (600 MHz, $CDCl_3$) δ 1.04 (t, J = 7.6 Hz, 3H), 2.40 (q, J = 7.6 Hz, 2H), 2.73 (t, J = 8.3 Hz, 2H), 2.90 (t, J = 8.2 Hz, 2H), 7.16–7.21 (m, 3H), 7.24–7.30 (m, 2H) ppm.; $^{13}C\{^1H\}$ NMR (150 MHz, $CDCl_3$) δ 7.7, 29.8, 36.1, 43.9, 126.0, 128.3, 128.4, 141.2, 210.6 ppm.

1,3-Diphenylpropan-1-one (KE-**c**)[32] white solid: ^{1}H NMR (600 MHz, $CDCl_3$) δ 3.07 (t, *J* = 7.6 Hz, 2H), 3.30 (t, *J* = 7.6 Hz, 2H), 7.20 (t, *J* = 7.6 Hz, 1H), 7.25 (d, *J* = 6.9 Hz, 2H), 7.30 (t, *J* = 7.6 Hz, 2H), 7.45 (t, *J* = 7.5 Hz, 2H), 7.55 (t, *J* = 7.6 Hz, 1H), 7.96 (d, *J* = 6.9 Hz, 2H) ppm.; ^{13}C{^{1}H} NMR (150 MHz, $CDCl_3$) δ 30.1, 40.4, 126.1, 128.0, 128.4, 128.5, 128.6, 133.0, 136.8, 141.3, 199.2 ppm.

4.4. Intramolecular aldol condensation

Representative procedure for intramolecular aldol condensation using rhenium complex : Re complex = $ReOCl_3(O{=}PPh_3)(SMe_2)$: (Fig. 4, c)

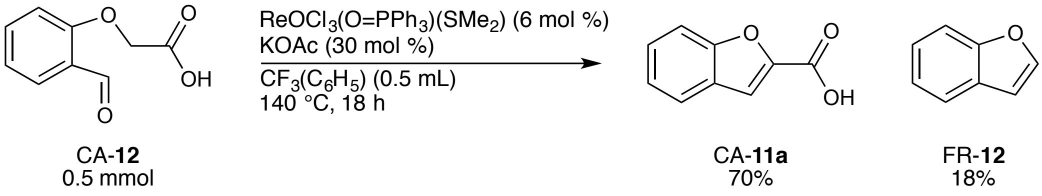

2-Formylphenoxyacetic acid (CA-**12**) (0.5 mmol, 90.1 mg), KOAc (0.15 mmol, 14.7 mg), $ReOCl_3(O{=}PPh_3)(SMe_2)$ (0.03 mmol, 19.5 mg) and a magnetic stirrer bar were placed in a dried up 75 mL J. Young tube, which was purged Ar gas several times. Anhydrous trifluorotoluene (0.5 mL) was added under a continuous flow of Ar. The J. Young tube was heated at 140 °C, where the mixture was stirred (500 rpm) for 18 h and cooled to 0 °C. The residue was dissolved in DMSO-d_6 and analyzed by ^1H NMR spectroscopy. The benzofuran-2-carboxylic acid (CA-**11a**) (70%) and benzofuran (FR-**12**) (18%) were calculated based on the integration ratio of their signals relative to the internal standard 1,1,2,2-tetrachloroethane.

The structure of desired carboxylic acid (CA-**11a**) was double confirmed after isolation, and the spectral data were consistent with that of the authentic sample purchased from a chemical company.

CA-**11a** was isolated by the following extraction method: a NMR sample solution diluted with saturated $NaHCO_3$ aqueous solution (30 mL) was extracted with EtOAc (15 mL×3). The water layer was acidified to pH 2–3 with citric acid, and extracted with EtOAc (15 mL×3). The combined organic layer was washed with brine and dried over anhydrous Na_2SO_4. After removing the solvent under a reduced pressure, the residue was suspended in $CHCl_3$. The suspension was passed through the Celite pad, and solvent was removed to give desired carboxylic acid (CA-**11a**) as a slightly yellow solid.

Benzofuran-2-carboxylic acid (CA-**11a**), slightly yellow solid: ^1H NMR (600 MHz, DMSO-d_6) δ 7.35 (t, J = 8.2 Hz, 1H), 7.50 (t, J = 8.2 Hz, 1H), 7.66 (s, 1H), 7.70 (d, J = 9.7 Hz, 1H), 7.79 (d, J = 8.3 Hz, 1H) ppm.; $^{13}C\{^1H\}$ NMR (150 MHz, DMSO-d_6) δ 112.0, 113.4, 123.1, 123.8, 126.8, 127.5, 146.2, 155.0, 160.1 ppm.

4.5. Investigation of Re species generated upon hydrogenation

Representative procedure for investigating the chemical species generated in hydrogenation of 3-phenylpropionic acid (CA-a) using Re-b (Additive: $KBPh_4$, Supplementary Figure 1)

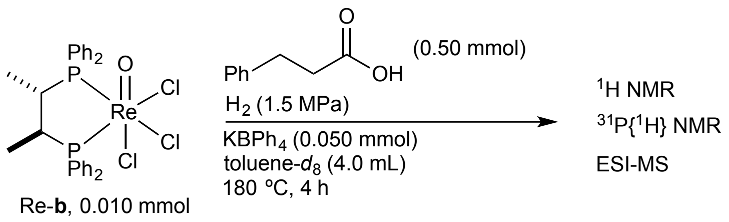

Re complex Re-**b** (7.4 mg, 0.010 mmol), $KBPh_4$ (17.9 mg, 0.050 mmol), 3-phenylpropinic acid (CA-**a**) (75.1 mg, 0.50 mmol) and magnetic stirring bar were placed in a glass tube. The glass tube was inserted into an autoclave, which was closed tightly, evaporated in vacuum, and refilled with Ar gas. To the mixture was added toluene-d_8 (3 mL) under a continuous flow of Ar, and inside the autoclave was purged several times with H_2 gas (P_{H2} = 1.0 MPa). The autoclave was pressurized by H_2 gas (P_{H2} = 1.5 MPa) at room temperature, and heated at 180 ºC for 4 h with stirring. The autoclave was cooled to 0 ºC in an ice–water bath. The reaction mixture was analyzed by ^1H and $^{31}P\{^1H\}$ NMR, and ESI-MS. The sample for ESI-MS analysis was prepared by further diluting the resulting reaction mixture with acetonitrile.

4.6. Investigation of Re species generated upon deuteration reaction

Representative procedure for investigating the chemical species generated in deuteration of α-C–H of 3-phenylpropionic acid (CA-a) using Re-b (Additive: KBPh$_4$, Supplementary Figure 6)

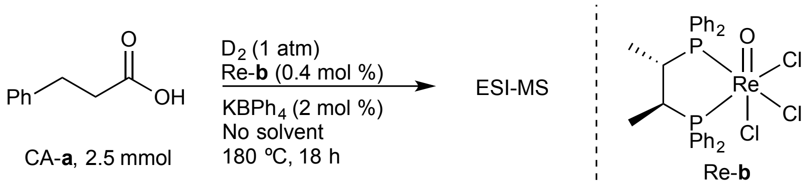

3-Phenylpropionic acid (CA-**a**) (2.5 mmol, 375.4 mg), KBPh$_4$ (0.05 mmol, 17.9 mg), ReOCl$_3$[(*S*,*S*)-Chiraphos] (Re-**b**) (0.01 mmol, 7.3 mg) and a magnetic stirrer bar were placed in a dried up 75 mL J. Young tube equipped with a three ways cock and a rubber balloon, which was purged Ar gas several times and filled with D$_2$ (P_{D2} = 1 atm). The J. Young tube was heated at 180 °C, where the mixture was stirred (500 rpm) for 4 h and cooled to 0 °C. The residue was dissolved in CDCl$_3$ and further diluted with acetonitrile, and analyzed ESI-MS.

4.7. Investigation of Re species generated upon unsymmetrical ketones synthesis

Representative procedure for investigating the chemical species generated in unsymmetrical ketones synthesis using Re-b (Additive: KOAc, Electrophile: acetic anhydride, Supplementary Figure 5)

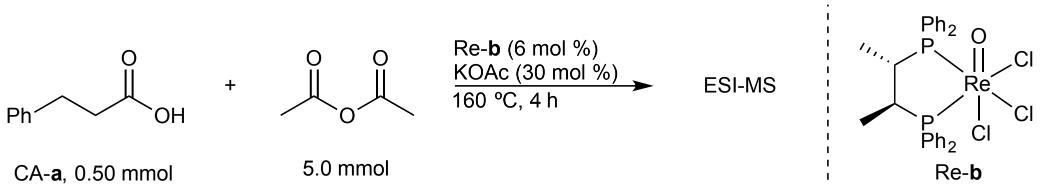

3-Phenylpropionic acid (CA-**a**) (0.5 mmol, 75.0 mg), KOAc (0.15 mmol, 14.7 mg), $ReOCl_3$[(*S*,*S*)-Chiraphos] (Re-**b**) (0.03 mmol, 22.0 mg) and a magnetic stirrer bar were placed in a dried up 75 mL J. Young tube, which was purged Ar gas several times. Acetic anhydride (5.0 mmol, 470 μL) was added under a continuous flow of Ar. The J. Young tube was heated at 160 °C, where the mixture was stirred (500 rpm) for 4 h and cooled to 0 °C. The reaction mixture was diluted with acetonitrile and analyzed by ESI-MS.

4.8. Precatalyst preparation

4.8.1. Oxotrichloro[1,2-bis(diphenylphosphino)ethane]rhenium (V) (Re-a)

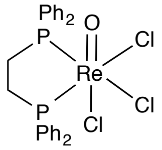

Title complex was synthesized following the known procedure.[34]

Oxotrichloro[(dimethylsulfide)triphenylphosphineoxide]rhenium (0.321 mmol, 201 mg), 1,2-bis(diphenylphosphio)ethane (dppe) (0.324 mmol, 129 mg), THF (5 mL) and magnetic stirring bar were placed in a vessel equipped with a Young's stopcock (30 mL) under Ar gas atmosphere. The mixture was stirred at 75 ºC for 2 h and cooled to room temperature. The resulting blue suspension was filtered under air, washed with ethanol (4 mL×2) and diethyl ether (4 mL×2), and dried under vacuum to obtain desired rhenium complex (Re-**a**) as sky-blue solid (184 mg, 83%). ^{1}H NMR (500 MHz, DMSO-d_6) δ: 3.20–3.35 (m, 2H), 3.76–3.95 (m, 2H), 7.52 (bs, 12H), 8.00–8.20 (m, 8H) ppm. ^{31}P{^{1}H} NMR (202 MHz, DMSO-d_6) δ: 5.0 ppm.; HRMS (FAB, $(M–Cl)^+$) Calcd for $C_{26}H_{24}O_1P_2Cl_2Re^+$: 671.0237; Found: *m/z* = 671.0230. The spectral data were almost consistent with the reported values.[34]

4.8.2. Oxotrichloro[(*S*,*S*)-Chiraphos]rhenium (V) (Re-b)

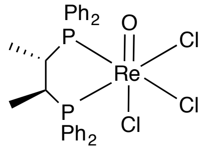

Oxotrichloro[(dimethylsulfide)triphenylphosphineoxide]rhenium (0.104 mmol, 67 mg), (2*S*, 3*S*)-(–)-bis(dipheylphosphino)butane ((*S*,*S*)-Chiraphos) (0.108 mmol, 46 mg), THF (3 mL) and magnetic stirring bar were placed in a vessel equipped with a Young's stopcock (30 mL) under Ar gas atmosphere. The mixture was stirred at 75 ºC for 3 h and cooled to room temperature. The resulting sky blue suspension was filtered under air, washed with ethanol (2 mL×2) and diethyl ether (2 mL×2), and dried under vacuum to obtain desired rhenium complex (Re-**b**) as sky-blue solid (57 mg, 75%). IR (KBr): 1448 (w), 1434 (s), 1096 (w), 973 (s, Re=O), 751 (w), 742 (w), 711 (w), 691 (s) cm^{-1}.; ^{1}H NMR (600 MHz, DMSO-d_6) δ: 1.22 (dd, J = 13.8, 6.9 Hz, 3H), 1.67 (dd, J = 13.8, 6.9 Hz, 3H), 3.15–3.25 (m, 1H), 4.12–4.22 (m 1H), 7.18 (t, J = 8.4 Hz, 2H), 7.40 (t, J = 7.8 Hz, 2H), 7.44–7.55 (m, 4H), 7.58–7.73 (m, 8H), 7.98 (t, J = 7.8 Hz, 2H), 8.19 (t, J = 7.8 Hz, 2H) ppm.; $^{13}C\{^{1}H\}$ NMR (DMSO-d_6, 150 MHz): δ 15.0 (d, $^{2}J_{P\text{-}C}$ = 12.2 Hz), 16.1 (d, $^{2}J_{P\text{-}C}$ = 13.6 Hz), 16.8 (dd, $^{1}J_{P\text{-}C}$ = 21.6 Hz, $^{2}J_{P\text{-}C}$ = 20.1 Hz), 17.0 (dd, $^{1}J_{P\text{-}C}$ = 21.5 Hz, $^{2}J_{P\text{-}C}$ = 21.6 Hz), 128.4 (2C) (d, J = 10.1 Hz), 128.6 (2C) (d, J = 10.1 Hz), 129.4 (2C) (d, J = 8.6 Hz), 129.8 (2C) (d, J = 10.1 Hz), 132.0 (2C) (d, J = 43.1 Hz), 132.8 (2C) (d, J = 11.5 Hz), 133.8–133.9 (4C) (m), 134.7 (2C) (d, J = 8.6 Hz), 135.1 (4C) (d, J = 7.2 Hz), 135.2 (2C) (d, J = 8.6 Hz).; $^{31}P\{^{1}H\}$ NMR (242 MHz, DMSO-d_6) δ: 16.7, 9.5 ppm.; HRMS (FAB, $(M–Cl)^{+}$) Calcd for $C_{28}H_{28}O_{1}P_{2}Cl_{2}Re^{+}$: 699.0550; Found: *m/z* = 699.0578.; elemental analysis calcd (%) for $C_{28}H_{28}O_{1}P_{2}Cl_{3}Re$: C 45.75, H 3.84; found: C 46.07, H 4.01; $[\alpha]_D^{20.4}$ = –58.6 (*c* 0.2, CH_3OH).

4.8.3. Oxotrichloro[1,3-bis(diphenylphosphino)propane]rhenium (V) (Re-d)

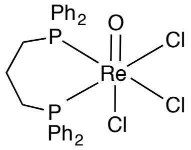

Oxotrichloro[(dimethylsulfide)triphenylphosphineoxide]rhenium (0.104 mmol, 67 mg), 1,3-bis(diphenylphosphino)propane (dppp) (0.108 mmol, 45 mg), THF (2 mL) and magnetic stirring bar were placed in a vessel equipped with a Young's stopcock (30 mL) under Ar gas atmosphere. The mixture was stirred at 75 ºC for 3 h and cooled to room temperature. Diethyl ether (10 mL) was added to give blue precipitate. The precipitate was collected by filtration under air, washed with ethanol (2 mL×2) and diethyl ether (2 mL×2) and dried under vacuum to obtain desired rhenium complex (Re-**d**) as blue solid (61 mg, 81%). ^{1}H NMR (600 MHz, CD_2Cl_2) δ: 2.21–2.36 (m, 1H), 2.68–2.88 (m, 1H), 3.06–3.20 (m, 2H), 3.38–3.52 (m, 2H), 7.35–7.41 (m, 4H), 7.42–7.51 (m, 8H), 7.63–7.70 (m, 8H) ppm.; ^{13}C{^{1}H} NMR (150 MHz, CD_2Cl_2) δ: 19.5, 26.8–27.3 (m, 2C), 128.0 (t, $J_{P\text{–}C}$ = 4.4 Hz , 4C), 128.9 (t, $J_{P\text{–}C}$ = 4.4 Hz , 4C), 129.8–130.3 (m, 2C), 131.2 (2C), 131.5–132.1 (m, 2C), 131.9 (2C), 133.4 (t, $J_{P\text{–}C}$ = 4.4 Hz , 4C), 133.7 (t, $J_{P\text{–}C}$ = 4.4 Hz , 4C).; ^{31}P{^{1}H} NMR (240 MHz, CD_2Cl_2) δ: –25.9 ppm.; HRMS (FAB, $(M–Cl)^+$) Calcd for $C_{27}H_{26}O_1P_2Cl_2Re^+$: 685.0393; Found: *m/z* = 685.0395. The spectral data were roughly consistent with the values reported.[35]

4.8.4. Oxotrichloro[1,2-bis(diphenylphosphino)benzene]rhenium (V) (Re-e)

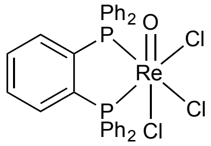

Oxotrichloro[(dimethylsulfide)triphenylphosphineoxide]rhenium (0.104 mmol, 67 mg), 1,2-bis(diphenylphosphino)benzene (dppbz) (0.108 mmol, 48 mg), THF (2 mL) and magnetic stirring bar were placed in a vessel equipped with a Young's stopcock (30 mL) under Ar gas atmosphere. The resulting suspension was stirred for 4 h at 75 °C. The solid was collected by filtration under air, washed with ethanol (2 mL×2) and diethyl ether (2 mL×2) and dried under vacuum. The complex was purified by column chromatography on silica gel (eluent; CH_2Cl_2/AcOEt= 7/3) to afford desired rhenium complex (Re-**e**) as blue-green solid (40 mg, 51%). ^{1}H NMR (600 MHz, CD_2Cl_2) δ:

7.25–7.31 (m, 4H), 7.34–7.40 (m, 2H), 7.43–7.63 (m, 11H), 7.77–7.84 (m, 3H), 7.87–7.92 (m, 2H), 8.05–8.10 (m, 2H) ppm.; $^{31}P\{^1H\}$ NMR (240 MHz, CD_2Cl_2) δ: 19.9 ppm.; HRMS (FAB, $(M–Cl)^+$) Calcd for $C_{30}H_{24}O_1P_2Cl_2Re^+$: 719.0237; Found: *m/z* = 719.0263. The spectral data were almost consistent with the values reported, other than $^{31}P\{^1H\}$ NMR ($^{31}P\{^1H\}$ NMR ($CDCl_3$) δ 18.5).[35]

4.8.5. Oxotrichloro[4,5-bis(diphenylphosphino)-9,9-dimethylxanthene]rhenium (V) (Re-f)

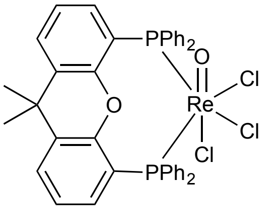

Oxotrichloro[(dimethylsulfide)triphenylphosphineoxide]rhenium (0.104 mmol, 67 mg), 4,5-bis(diphenylphosphino)-9,9-dimethylxanthene (Xantphos) (0.108 mmol, 62 mg), THF (2 mL) and magnetic stirring bar were placed in a vessel equipped with a Young's stopcock (30 mL) under Ar gas atmosphere. The resulting suspension was stirred for 2 h at 75 °C. The solid was collected by filtration under air, washed with ethanol (2 mL×2) and diethyl ether (2 mL×2) and dried under vacuum to obtain desired rhenium complex (Re-**f**) as blue-green solid (78 mg, 84%). ^1H NMR (600 MHz, CD_2Cl_2) δ: 1.46 (s, 3H), 1.88 (s, 3H), 6.90 (t, J = 7.5 Hz, 2H), 7.00–7.14 (m, 8H), 7.18 (t, J = 7.5 Hz, 2H), 7.27 (t, J = 7.5 Hz, 2H), 7.37–7.45 (m, 4H), 7.45–7.57 (m, 6H), 7.68 (d, J = 7.5 Hz, 2H) ppm.; $^{31}P\{^1H\}$ NMR (240 MHz, CD_2Cl_2) δ: –36.7 ppm.; The spectral data were almost consistent with the values reported.[36]

4.8.6. Oxotrichloro[(*S*,*S*)-DIPAMP]rhenium (V) (Re-g)

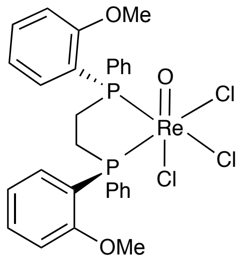

Oxotrichloro[(dimethylsulfide)triphenylphosphineoxide]rhenium (0.104 mmol, 67 mg), (1*S*,2*S*)-(+)-bis[(2-methoxyphenyl)phenylphosphino]ethane ((*S, S*)- DIPAMP) (0.108 mmol, 50 mg), THF (3 mL) and magnetic stirring bar were placed in a vessel equipped with a Young's stopcock (30 mL) under Ar gas atmosphere. The resulting blue color solution was stirred for 3 h at 75 °C after cooled at room temperature diethyl ether was added (10 mL) to give light blue precipitate. The precipitate was collected by filtration under air, washed with diethyl ether (2 mL×2) and dried under vacuum to obtain desired rhenium complex (Re-**g**) as light blue solid (54 mg, 68%). IR (KBr): 1588 (m), 1573 (w), 1478 (s), 1461 (m), 1433 (s), 1282 (m), 1253 (s), 1019 (m), 980 (m, Re=O), 799 (w), 750 (m), 692 (m) cm^{-1}.; ^{1}H NMR (600 MHz, DMSO-d_6) δ: 2.73–2.86 (m 1H), 3.20–3.30 (m, 1H), 3.61 (s, 3H), 3.70 (s, 3H), 3.80–3.98 (m, 1H), 3.98–4.13 (m, 1H), 7.00–7.08 (m, 4H), 7.37 (bs, 3H), 7.41 (bs, 3H), 7.46–7.54 (m, 2H), 7.85–7.94 (m, 4H), 7.96–8.03 (m, 1H), 8.41–8.47 (m, 1H) ppm.; ^{13}C{^{1}H} NMR (DMSO-d_6, 150 MHz): δ 31.4 (d, $^{1}J_{P\text{-}C}$ = 40.2 Hz), 31.5 (d, $^{1}J_{P\text{-}C}$ = 37.3 Hz), 55.6, 55.7, 112.1 (d, J = 4.3 Hz), 112.5 (d, J = 2.9 Hz), 119.2 (d, J = 57.5 Hz), 120.1 (d, J = 11.5 Hz), 120.1 (d, J = 48.8 Hz), 120.7 (d, J = 11.5 Hz), 127.9 (2C) (d, J = 10.1 Hz), 128.2 (2C) (d, J = 10.1 Hz), 130.6, 130.9, 131.8–131.9 (4.5C) (m), 132.3 (0.5C), 133.6 (d, J = 48.8 Hz), 133.8, 134.5, 135.9 (d, J = 12.9 Hz), 136.3 (d, J = 11.5 Hz), 160.7, 161.3 ppm.; ^{31}P{^{1}H} NMR (242 MHz, DMSO-d_6) δ: 4.2, 3.1 ppm.; HRMS (FAB, $(M–Cl)^{+}$) Calcd for $C_{28}H_{28}O_3P_2Cl_2Re^{+}$: 731.0488; Found: *m/z* = 731.0487.; elemental analysis calcd (%) for $C_{28}H_{28}O_3P_2Cl_3Re$: C 43.85, H 3.68; found: C 43.84, H 3.67; $[\alpha]_D^{20.6}$ = –40.8 (*c* 0.2, CH_3OH).

4.8.7 Trichloro (η^3-Triphos) rhenium (III) (Re-l)

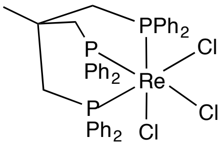

The title complex was synthesized following the known procedure.[37]

To a hot suspension of (acetonitrile)trichlorobis(triphenylphosphine)rhenium(III) (**1k**) (0.09 mmol, 76 mg) in 4 mL anhydrous toluene was added 1,1,1-tris(diphenylphosphinomethyl)ethane (Triphos) (0.16 mmol, 99.9 mg). The resulting reaction mixture was stirred for 2 h at 125 °C to give the yellow-green precipitate. After cooled at room temperature, the precipitate was collected by filtration under air and recrystallized by CH_2Cl_2/Et_2O to obtain desired complex Re-**l** as yellow-green powder (56 mg, 68%). ^1H NMR (500 MHz, CD_2Cl_2) δ: 5.86 (s, 3H), 6.94 (t, J = 7.4 Hz, 6H), 8.29 (s, 6H), 8.48 (t, J = 7.4 Hz, 12H), 10.12 (d, J = 6.9 Hz, 12H) ppm.

4.9. Substrate preparation for hydrogenation

4.9.1. (*E*)-3-(4-(methoxycarbonyl)phenyl)acrylic acid (CA-n)

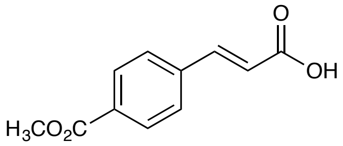

(*E*)-3-(4-(methoxycarbonyl)phenyl)acrylic acid (CA-**n**) was prepared following the known procedure.[38]

To the solution of malonic acid (22 mmol, 2.3 g) in dry pyridine (10 mL) were added methyl 4-formylbenzoate (18 mmol, 3 g) and piperidine (0.2 mL) at room temperature. The resulting solution was stirred for 3 h at 90 ºC, then the reaction temperature was raised up to 115 ºC. After stirring for 1.5 h at this temperature, the reaction mixture was cooled to room temperature, and then poured into 3 N HCl aqueous solution (125 mL). The formed white precipitate was filtered and washed with water (5×100 mL) and ethanol (100 mL), then dried under vacuum to obtained white solid powder (3.2 g, 86%). ^{1}H NMR (600 MHz, DMSO-d_6) δ: 3.86 (s, 3H), 6.63 (d, J = 15.8 Hz, 1H), 7.64 (d, J = 15.8 Hz, 1H), 7.82 (d, J = 8.2 Hz, 2H), 7.96 (d, J = 8.2 Hz, 2H) ppm.; ^{13}C{^{1}H} NMR (150 MHz, DMSO-d_6) δ: 52.2, 121.9, 128.4, 129.5, 130.5, 138.7, 142.4, 165.7, 167.2 ppm.; HRMS (FAB, (M+H)$^+$) Calcd for $C_{11}H_{11}O_4^+$: 207.0652; Found: m/z = 207.0696.

4.9.2. 3-Pivalamidopropanoic acid (CA-q)

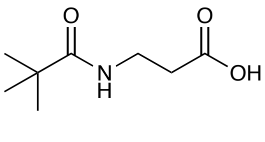

3-Pivalamidopropanoic acid (CA-**q**) was prepared following the known procedure.[39]

β-Alanine (2.2 g, 24.2 mmol) was dissolved in 1 N NaOH aqueous solution (25 mL) and diethyl ether (1 mL). The diethyl ether (5 mL) solution of pivaloyl chloride (2.5 mL, 20.3 mmol) was added over a period of 20 min. The resulting solution was stirred for 1.5 h at room temperature, then neutralized with 1 N HCl aqueous solutions. The mixture was extracted with ethyl acetate (3×25 mL), and combined organic layer was dried over anhydrous Na_2SO_4. Solvent was removed under reduced pressure and desired product was obtained as white solid (2 g, 47%). ^{1}H NMR (600 MHz, DMSO-d_6) δ: 1.05 (s, 9H), 2.35 (t, J = 6.8 Hz, 2H), 3.22 (q, J = 6.8 Hz, 2H), 7.49 (bs, 1H), 12.14 (bs,

1H) ppm.; $^{13}C\{^{1}H\}$ NMR (150 MHz, DMSO-d_6) δ: 27.3, 34.0, 35.1, 38.0, 172.9, 177.3 ppm.; HRMS (FAB, $(M+H)^+$) Calcd for $C_8H_{16}NO_3^+$: 174.1125; Found: *m/z* = 174.1126.

4.9.3. (*S*)-4-methyl-2-(1*H*-pyrrol-1-yl)pentanoic acid (CA-r)

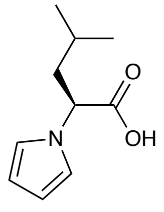

(*S*)-4-methyl-2-(1*H*-pyrrol-1-yl)pentanoic acid was synthesized following the known procedure.[40] *L*-leucine (1.31 g, 10 mmol) and 2,5-dimethoxytetrahydrofuran (1.3 mL, 10 mmol) were dissolved in water (10 mL), acetic acid (5 mL) and 1,2-dichloroethane (15 mL). The resulting solution was vigorously stirred for 45 min at 83 °C, and cooled to room temperature. The aqueous layer was separated and extracted several time with dichloromethane. The combined organic layers were dried over anhydrous Na_2SO_4. Desire product was purified by column chromatography on silica gel (Et_2O only) as light yellow solid (1.5 g, 83%). IR (KBr): 2963 (w), 1717 (s), 1490 (w), 1295 (w), 1281 (m), 1095 (w), 729 (s) cm^{-1}.; ^{1}H NMR (600 MHz, $CDCl_3$) δ: 0.91 (d, *J* = 6.8 Hz, 3H), 0.93 (d, *J* = 6.8 Hz, 3H), 1.40–1.50 (m, 1H), 1.88–1.97 (m, 1H), 1.98–2.07 (m, 1H), 4.65–4.72 (m, 1H), 6.20 (s, 2H), 6.73 (s, 2H) ppm.; $^{13}C\{^{1}H\}$ NMR ($CDCl_3$, 150 MHz) δ: 21.5, 22.7, 24.6, 41.1, 59.9, 108.7 (2C), 120.1 (2C), 177.4 ppm.; $[\alpha]_D^{19.9}$ = –1.90 (1.5, CH_3OH).; HRMS (FAB, $(M+H)^+$) Calcd for $C_{10}H_{16}N_1O_2^+$: 182.1176; Found: *m/z* = 182.1161.

4.9.4. (*S*)-3-phenyl-2-(1*H*-pyrrol-1-yl)propanoic acid (CA-s)

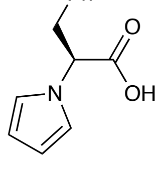

(*S*)-3-phenyl-2-(1*H*-pyrrol-1-yl)propanoic acid was synthesized following the known procedure.[40] *L*-phenylalanine (10 mmol, 1.65 g) and 2,5-dimethoxytetrahydrofuran (10 mmol, 1.3 mL) were dissolved in water (10 mL),

acetic acid (5 mL) and 1,2-dichloroethane (15 mL). The resulting solution was vigorously stirred for 45 min at 83 °C, and cooled to room temperature. The aqueous layer was separated and extracted several time with dichloromethane. The combined organic layers were dried over anhydrous Na_2SO_4. Desire product was purified by column chromatography on silica gel (Et_2O only) as light yellow solid (1.93 g, 89%). IR (KBr): 2384 (w), 1708 (s), 1488 (m), 1276 (m), 1098 (m), 744 (s), 730 (m), 698 (m) cm^{-1}.; ^{1}H NMR (600 MHz, $CDCl_3$) δ: 3.29 (dd, J = 13.7, 8.94 Hz, 1H), 3.44 (dd, J = 13.7, 8.90 Hz, 1H), 4.75–4.81 (m, 1H), 6.16 (t, J = 2.0 Hz, 2H), 6.70 (t, J = 2.0 Hz, 2H), 6.98–7.03 (m, 2H), 7.18–7.25 (m, 3H) ppm.; $^{13}C\{^{1}H\}$ NMR ($CDCl_3$, 150 MHz) δ: 39.0, 63.4, 109.0 (2C), 120.2 (2C), 127.2, 128.6 (2C), 128.8 (2C), 136.0, 179.1.; $[\alpha]_D^{19.3}$ = –72.81 (1.5, CH_3OH).; HRMS (FAB, $(M+H)^+$) Calcd for $C_{13}H_{14}N_1O_2^+$: 216.1019; Found: m/z = 216.1030.

4.9.5. (*R*)-2-(1*H*-pyrrol-1-yl)propanoic acid (CA-t)

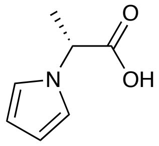

(*R*)-2-(1*H*-pyrrol-1-yl)propanoic acid was synthesized following the known procedure.[40] *D*-α-alanine (10 mmol, 0.89 g) and 2,5-dimethoxytetrahydrofuran (10 mmol, 1.3 mL) were dissolved in water (10 mL), acetic acid (5 mL) and 1,2-dichloroethane (15 mL). The resulting solution was vigorously stirred for 45 min at 83 °C, and cooled to room temperature. The aqueous layer was separated and extracted several time with dichloromethane. The combined organic layers were dried over anhydrous Na_2SO_4. Desire product was purified by column chromatography on silica gel (Et_2O only) as light yellow solid (0.92 g, 66%). IR (KBr): 3123 (w), 1731 (s), 1686 (w), 1203 (w), 1184 (m), 1099 (w), 742 (s), 726 (w), 657 (w) cm^{-1}.; ^{1}H NMR (600 MHz, $CDCl_3$) δ: 1.77 (d, J = 7.6 Hz, 3H), 4.80 (q, J = 7.6 Hz, 1H), 6.21 (t, J = 2.1 Hz, 2H), 6.74 (t, J = 2.1 Hz, 2H) ppm.; $^{13}C\{^{1}H\}$ NMR ($CDCl_3$, 150 MHz) δ: 17.9, 56.6, 108.9 (2C), 119.8 (2C), 177.5 ppm.; $[\alpha]_D^{20.3}$ = –16.21 (1.5, CH_3OH).; HRMS (FAB, $(M+H)^+$) Calcd for $C_7H_{10}N_1O_2^+$: 140.0706; Found: m/z = 140.0721.

4.10. New compounds obtained in hydrogenation and deuteration experiments

4.10.1. *N*-(3-hydroxypropyl)pivalamide (AL-q)

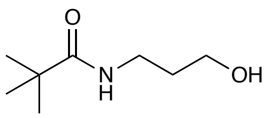

The reaction mixture obtained after the hydrogenation was purified by column chromatography on silica gel (eluent; hexane/ AcOEt / methanol= 7/2/1), giving AL-**q** as a colorless oil. IR (NaCl): 3345 (s), 2958 (s), 2872 (s), 1638 (m), 1536 (m), 1215 (w), 1069 (w) cm^{-1}.; ^{1}H NMR ($CDCl_3$, 600 MHz) δ: 1.20 (s, 9H), 1.65–1.70 (m, 2H), 3.41–3.45 (m, 2H), 3.60 (t, *J* = 5.4 Hz, 2H), 6.04 (bs, 1H) ppm.; $^{13}C\{^{1}H\}$ NMR ($CDCl_3$, 150 MHz) δ: 27.6, 32.3, 36.2, 38.7, 59.1, 180.0 ppm.; HRMS (FAB, $(M+H)^{+}$) Calcd for $C_8H_{18}N_1O_2^{+}$: 160.1332; Found: *m/z* = 160.1358.

4.10.2. 4-Methyl-2-(1*H*-pyrrol-1-yl)pentan-1-ol (*rac*-AL-r)

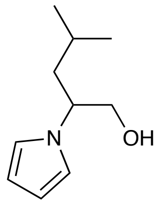

The reaction mixture obtained after hydrogenation was purified by column chromatography on silica gel (eluent; hexane/ AcOEt = 3/2), giving *rac*-AL-**r** as a colorless oil. IR (NaCl): 3409 (s), 2956 (s), 2869 (s), 1489 (s), 1467 (m), 1279 (w), 1090 (s), 1065 (s), 725 (w) cm^{-1}.; ^{1}H NMR ($CDCl_3$, 600 MHz) δ: 0.87 (d, *J* = 6.6 Hz, 3H), 0.92 (d, *J* = 6.6 Hz, 3H), 1.36–1.43 (m, 2H), 1.45–1.50 (m, 1H), 1.71–1.77 (m, 1H), 3.70–3.76 (m, 2H), 4.05–4.11 (m, 1H), 6.19 (t, *J* = 1.8 Hz, 2H), 6.72 (t, *J* = 1.8 Hz, 2H), ppm.; $^{13}C\{^{1}H\}$ NMR ($CDCl_3$, 150 MHz) δ: 21.8, 23.1, 24.5, 40.5, 60.3, 66.8, 108.3 (2C), 119.1 (2C) ppm.; HRMS (FAB, M^{+}) Calcd for $C_{10}H_{17}N_1O_1^{+}$: 167.1305; Found: *m/z* = 167.1276.

4.10.3. 3-Phenyl-2-(1*H*-pyrrol-1-yl)propan-1-ol (*rac*-AL-s)

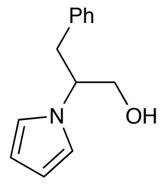

The reaction mixture obtained after hydrogenation was purified by column chromatography on silica gel (eluent; hexane/ AcOEt = 3/2), giving *rac*-AL-**s** as a colorless oil.

IR (NaCl): 3346 (s), 2958 (s), 2872 (s), 1638 (s), 1536 (s), 1214 (w) cm^{-1}. ^{1}H NMR ($CDCl_3$, 600 MHz) δ: 1.51 (t, J = 6.6 Hz, 1H), 3.02–3.11 (m, 2H), 3.82 (t, J = 6.6 Hz, 2H), 4.15–4.23 (m, 1H), 6.17 (t, J = 1.8 Hz, 2H), 6.70 (t, J = 1.8 Hz, 2H), 7.03 (t, J = 6.6 Hz, 2H), 7.17–7.29 (m, 3H) ppm.; $^{13}C\{^{1}H\}$ NMR ($CDCl_3$, 150 MHz) δ: 38.6, 63.5, 65.3, 108.5 (2C), 119.2 (2C), 126.7, 128.5 (2C), 128.8 (2C), 137.5 ppm.; HRMS (FAB, $(M+H)^{+}$) Calcd for $C_{13}H_{16}N_{1}O_{1}^{+}$: 202.1226; Found: m/z = 202.1227.

4.10.4. 2-(1*H*-pyrrol-1-yl)propan-1-ol (*rac*-AL-t)

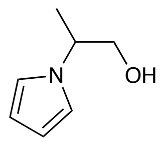

The reaction mixture obtained after hydrogenation was purified by column chromatography on silica gel (eluent; hexane/ AcOEt = 4/1), giving *rac*-AL-**t** as a colorless oil.

IR (NaCl): 3393 (s), 2975 (s), 2938 (s), 2878 (s), 1673 (s), 1491 (m), 1276 (m), 1052 (m), 727 (w), cm^{-1}.; ^{1}H NMR ($CDCl_3$, 600 MHz) δ: 1.46 (d, J = 6.8 Hz, 3H), 1.53 (bs, 1H), 3.66–3.77 (m, 2H), 4.15–4.22 (m, 1H), 6.19 (t, J = 1.8 Hz, 2H), 6.75 (t, J = 1.8 Hz, 2H) ppm.; $^{13}C\{^{1}H\}$ NMR ($CDCl_3$, 150 MHz) δ: 17.4, 57.1, 67.6, 108.4 (2C), 118.8 (2C) ppm.; HRMS (FAB, M^{+}) Calcd for $C_{7}H_{11}N_{1}O_{1}^{+}$: 125.0835; Found: m/z = 125.0836.

4.10.5. 3-Phenylpropanoic-2,2-d_2 acid (CA-a-d_2) 98% C2-deuterated

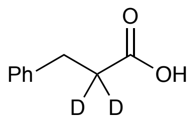

The reaction mixture obtained after deuteration was purified by column chromatography on silica gel (eluent; hexane/ AcOEt = 4/1), giving CA-**a**-d_2 as a white solid.

^{1}H NMR (600 MHz, $CDCl_3$) δ 2.94 (s, 2H), 7.18–7.23 (m, 3H), 7.26–7.31 (m, 2H) ppm, Small peak derived from residual α-C–H of CA-**a** was detected at 2.64–2.70 ppm.; $^{13}C\{^1H\}$ NMR (150 MHz, $CDCl_3$) δ 30.4, 34.6–35.7 (m), 126.3, 128.2, 128.5, 140.1, 179.4.; HRMS (FAB, $(M+H)^+$) Calcd for $C_9H_9D_2O_2^+$: 153.0879; Found: *m/z* = 153.0909.

4.10.6. 3-phenylpropan-1,1,2,2-d_4-1-ol (AL-a-d_4) 93% C1 and C2-deuterated

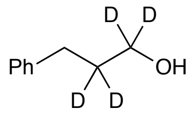

The reaction mixture obtained after deuteration was purified by column chromatography on silica gel (eluent; hexane/ AcOEt = 4/1), giving AL-**a**-d_4 as a colorless oil.

^{1}H NMR (600 MHz, $CDCl_3$) δ 1.50 (bs, 1H), 2.68 (s, 2H), 7.16–7.21 (m, 3H), 7.26–7.30 (m, 2H), Small peaks derived from α- and β-C–H of AL-**a** were detected at 1.84 and 3.61 ppm.; $^{13}C\{^1H\}$ NMR (150 MHz, $CDCl_3$) δ 31.7, 32.8–33.7 (m), 60.9–61.9 (m), 125.8, 128.30, 128.33, 141.8.; HRMS (FAB, $(M+H)^+$) Calcd for $C_9H_9D_4O_1^+$: 141.1212; Found: *m/z* = 141.1231.

5. Spectral data

5.1. New Re complexes

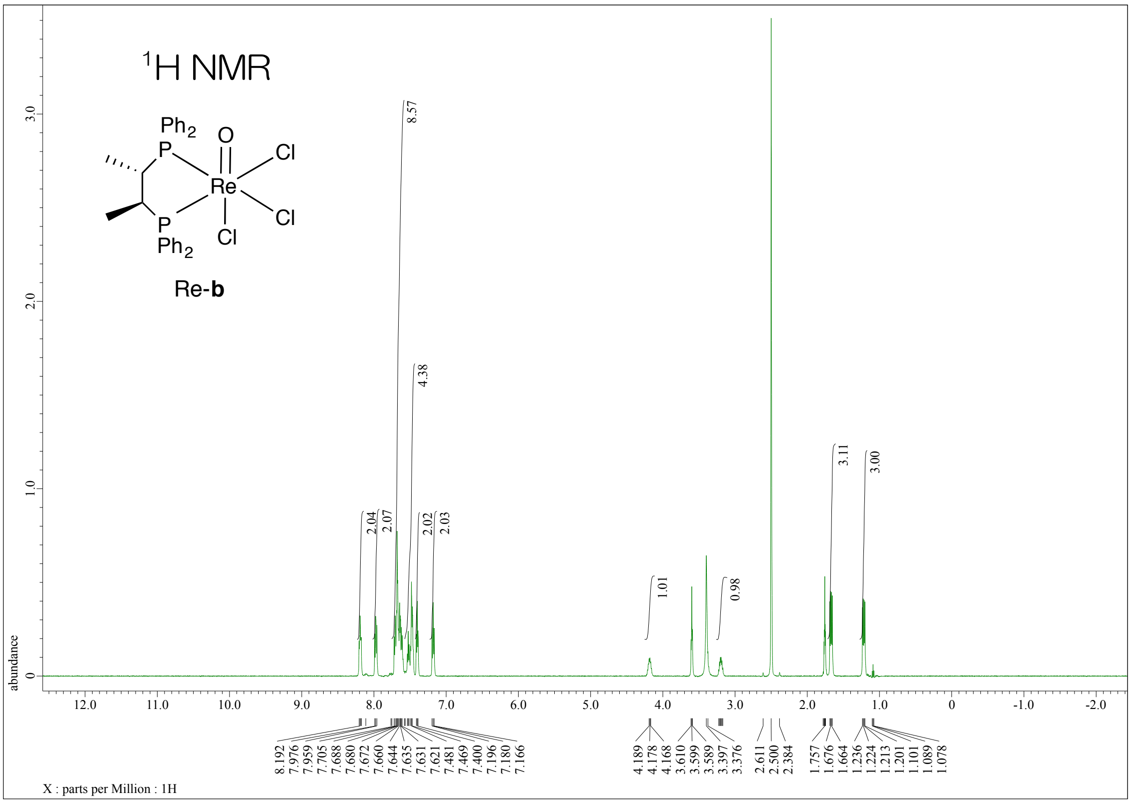

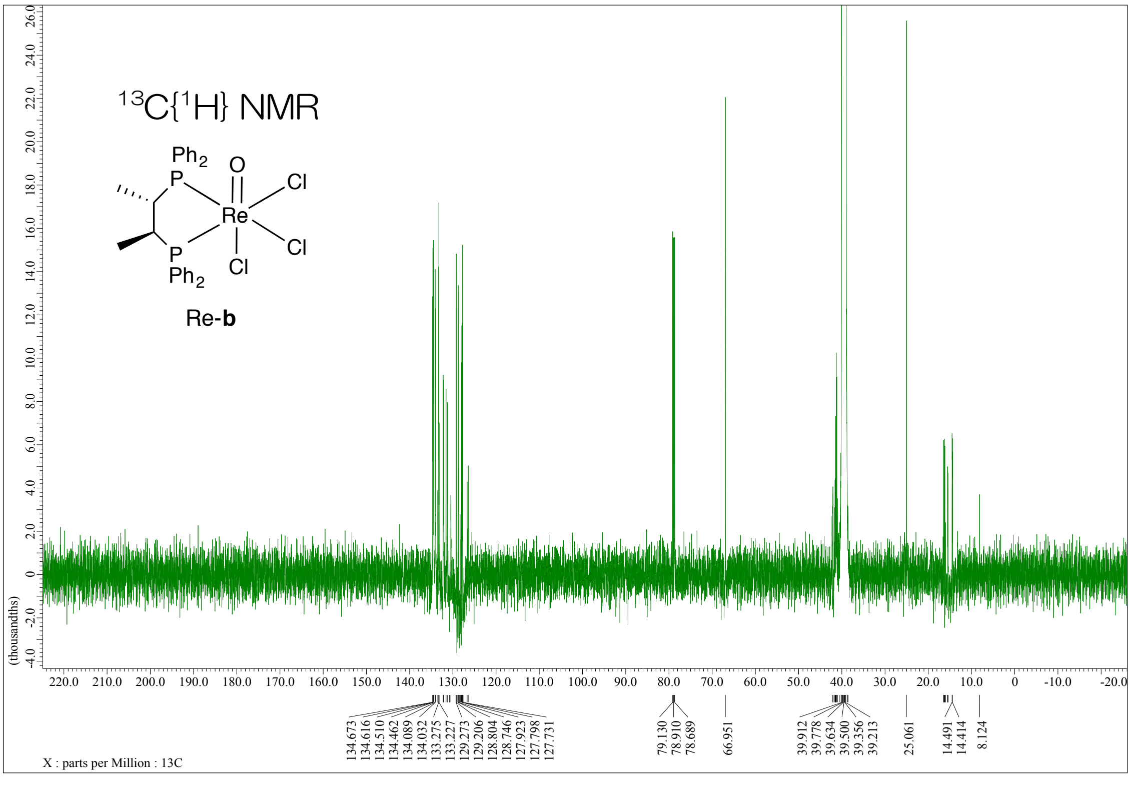

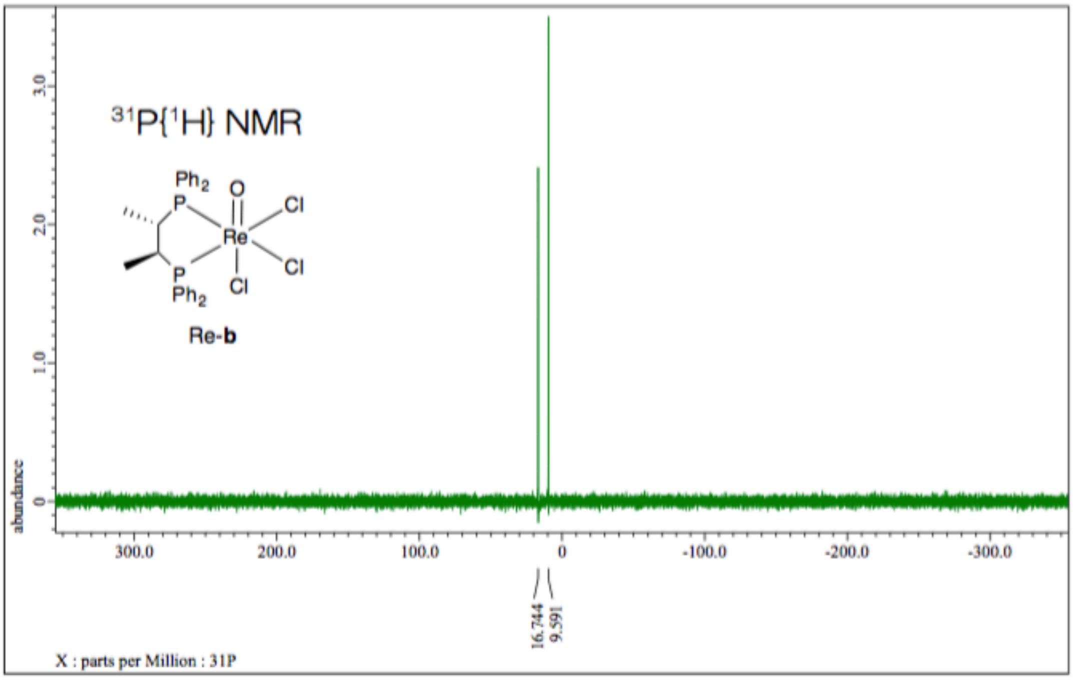

$^{31}P\{^1H\}$ NMR
Re-b
abundance
X : parts per Million : 31P
16.744
9.591

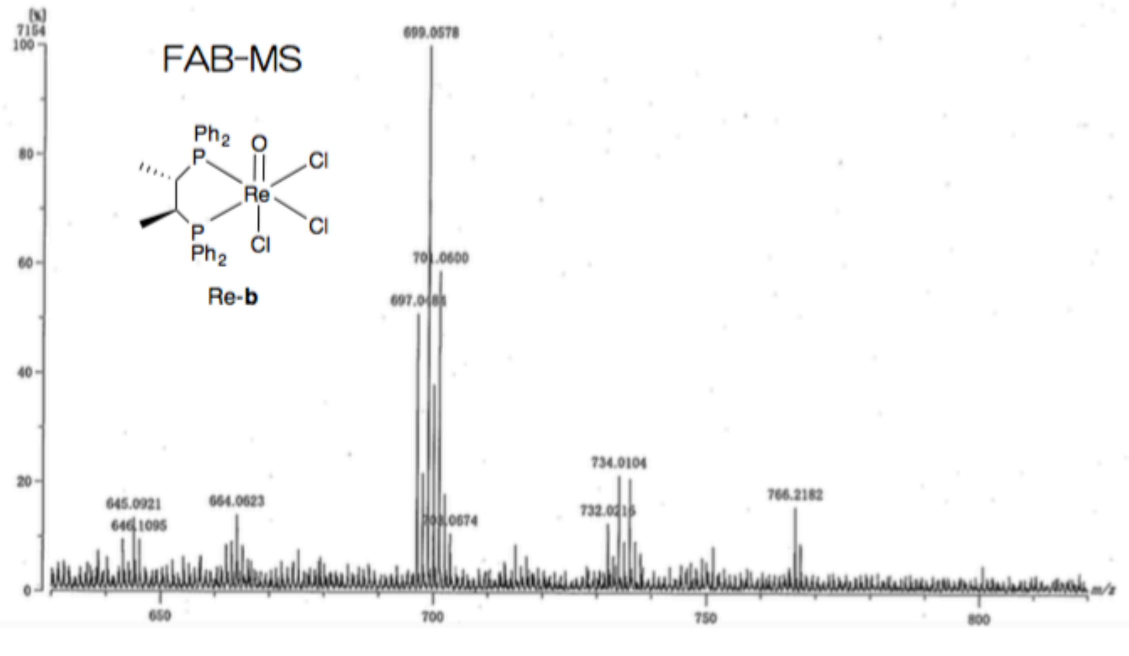

FAB-MS
Re-b
699.0578
645.0921
664.0623
734.0104
732.0215
766.2182
m/z

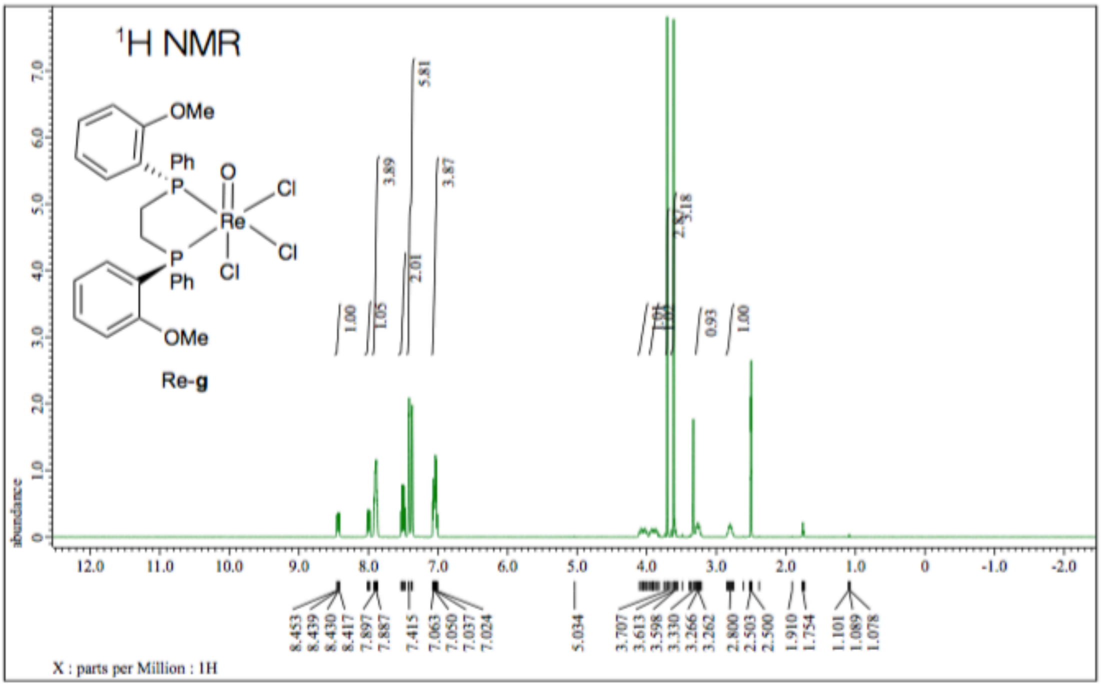

1H NMR
OMe
Ph
P
O
Re
Cl
Cl
P
Ph
Cl
OMe
Re-g
abundance
X : parts per Million : 1H

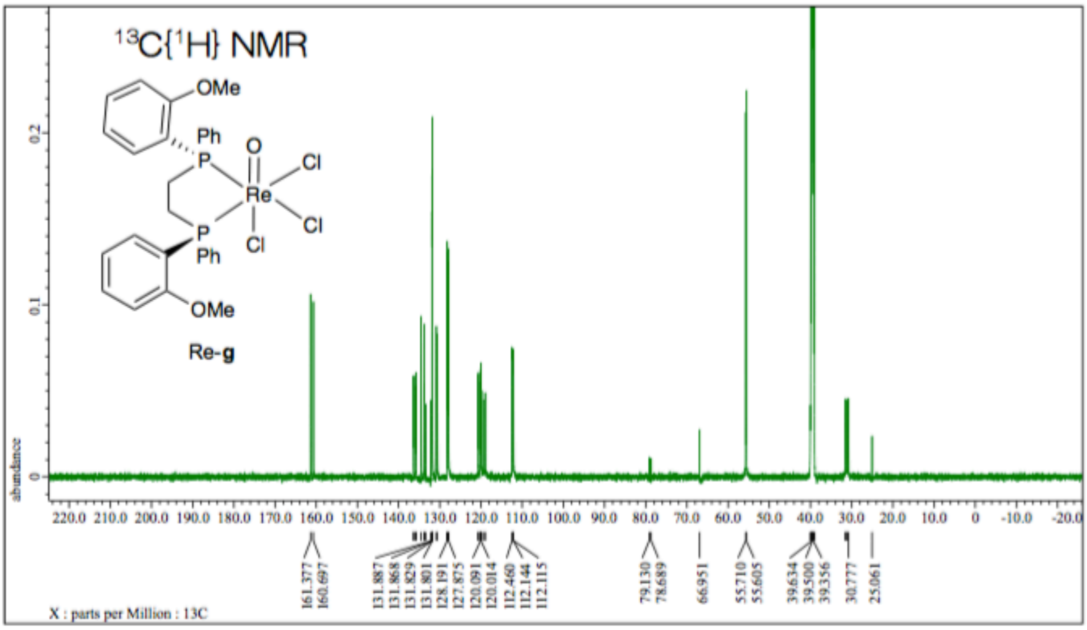

13C{1H} NMR
OMe
Ph
P
O
Re
Cl
Cl
P
Ph
Cl
OMe
Re-g
abundance
X : parts per Million : 13C

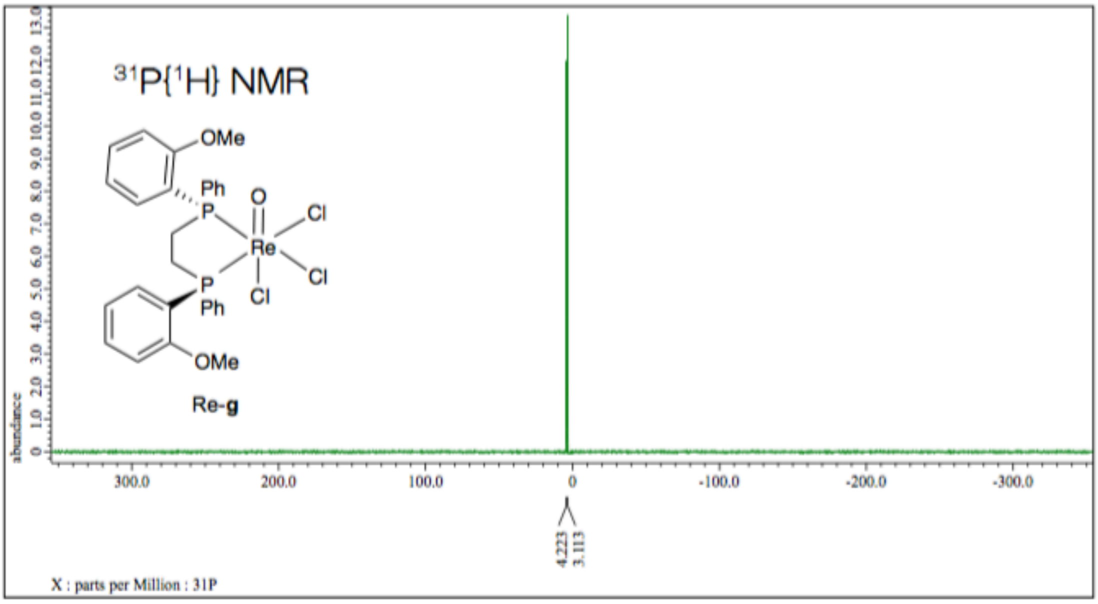

$^{31}P\{^1H\}$ NMR
OMe
Ph
P
O
Cl
Re
Cl
P
Ph
Cl
OMe
Re-**g**
abundance
300.0
200.0
100.0
0
-100.0
-200.0
-300.0
4.223
3.113
X : parts per Million : 31P

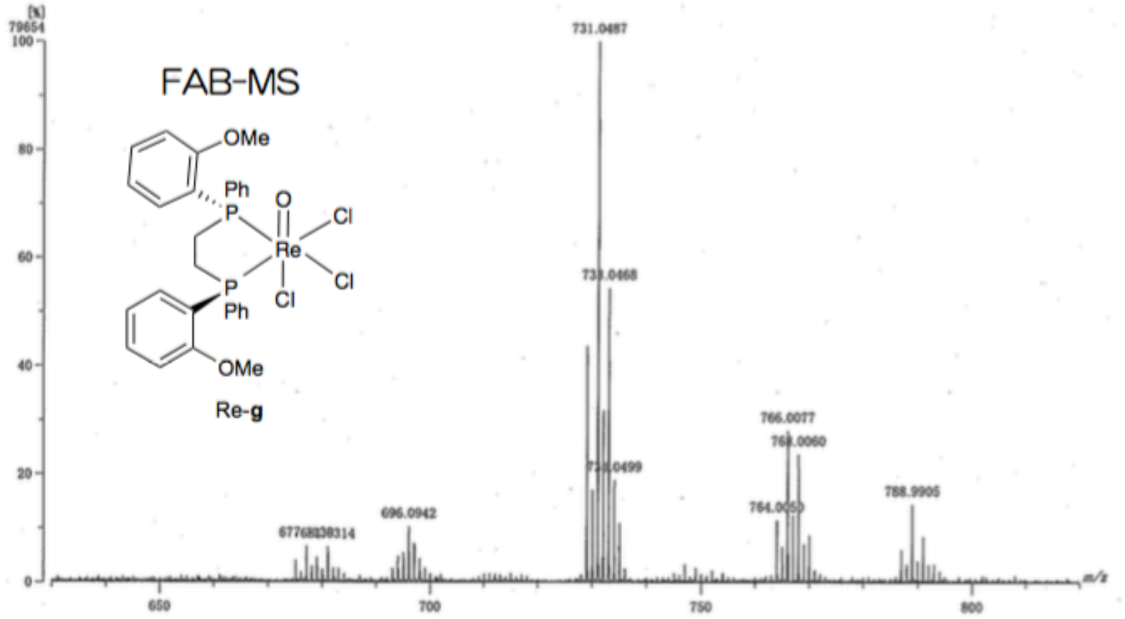

FAB-MS
OMe
Ph
P
O
Cl
Re
Cl
P
Ph
Cl
OMe
Re-**g**
731.0487
766.0077
696.0942
788.9905
650
700
750
800
m/z

5.2. NMR spectra of substrates synthesized

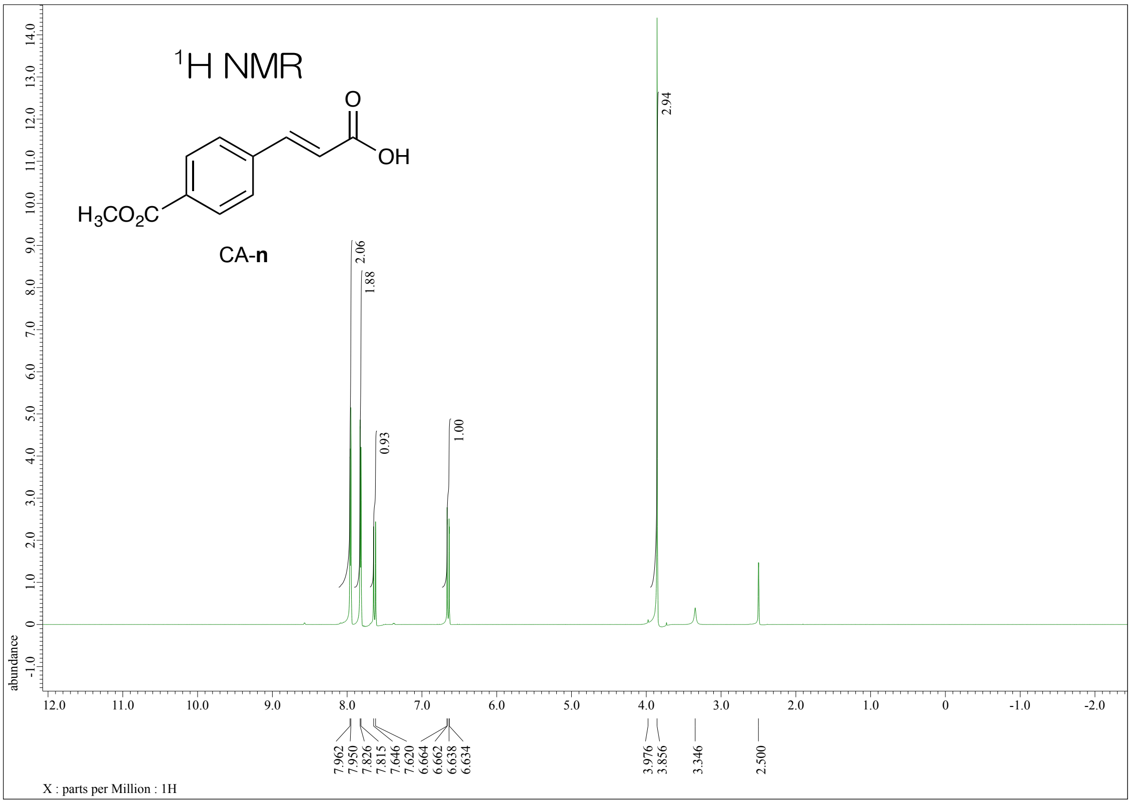

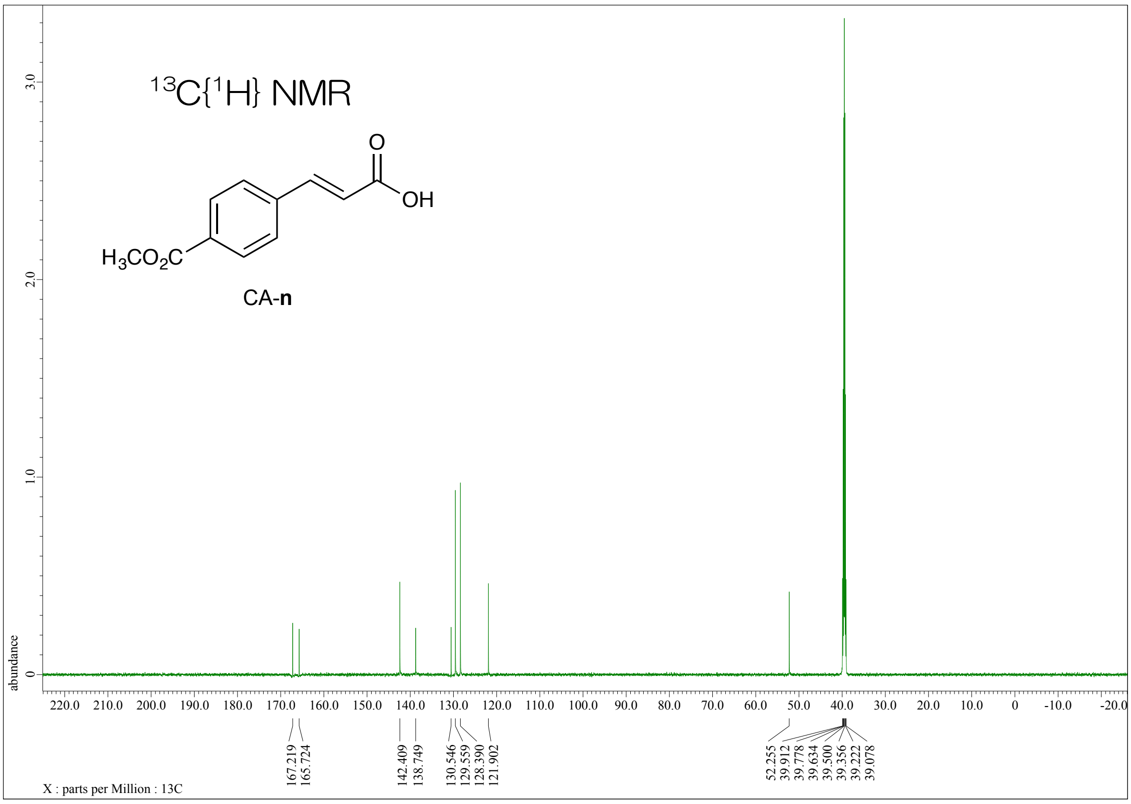

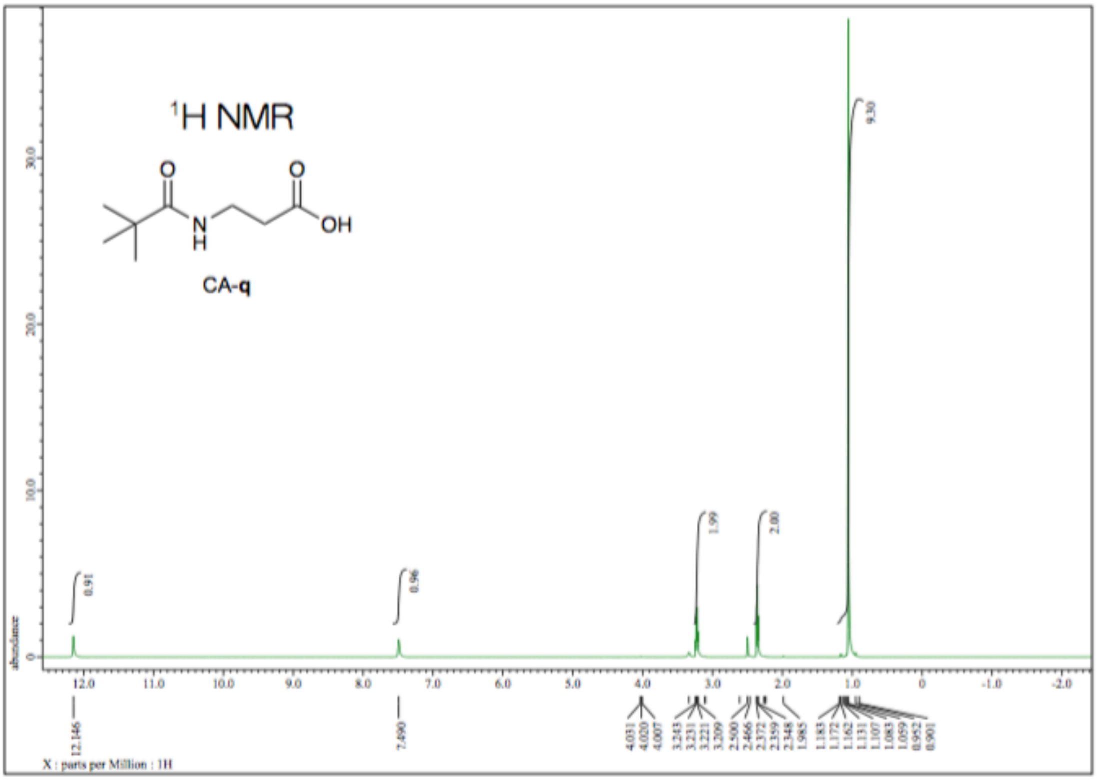

1H NMR
O
O
N
H
OH
CA-q
abundance
X : parts per Million : 1H
0.91
0.96
1.99
2.00
9.30
12.146
7.490
4.031
4.020
4.007
3.243
3.231
3.221
3.209
2.500
2.466
2.372
2.359
2.348
1.985
1.183
1.172
1.162
1.131
1.107
1.083
1.059
0.952
0.901

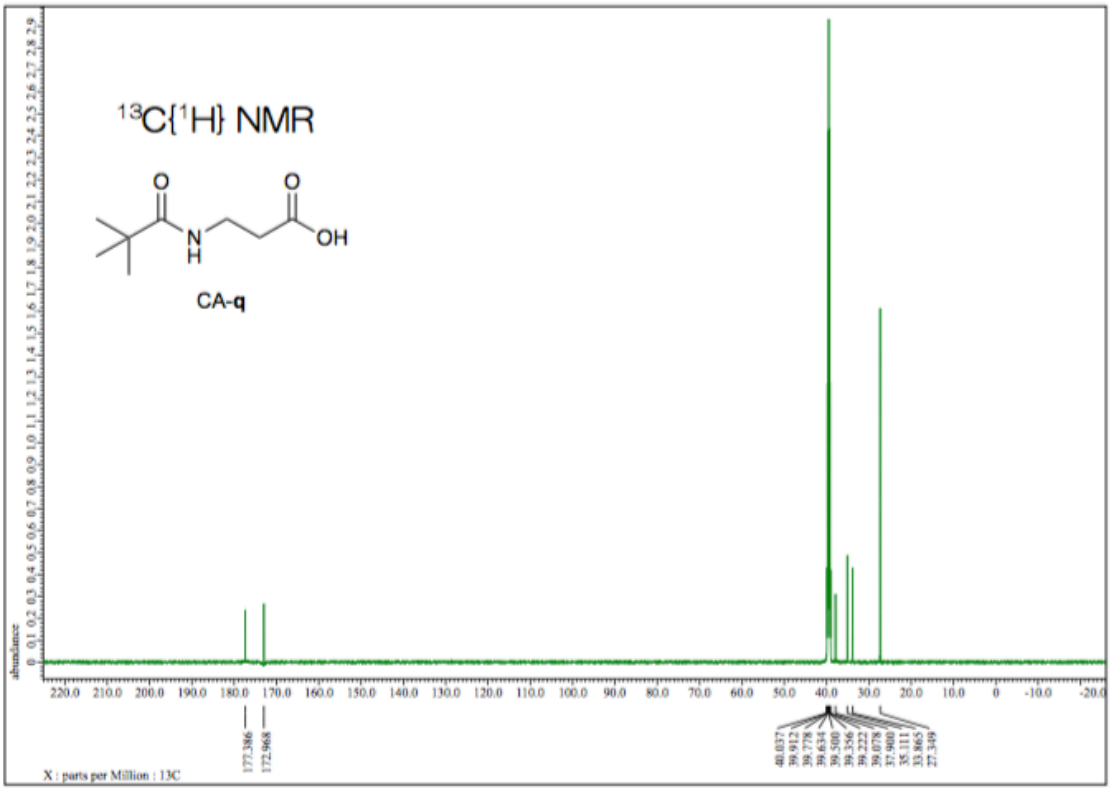

13C{1H} NMR
O
O
N
H
OH
CA-q
abundance
X : parts per Million : 13C
177.386
172.968
40.037
39.912
39.778
39.634
39.500
39.356
39.222
39.078
37.900
35.111
33.865
27.349

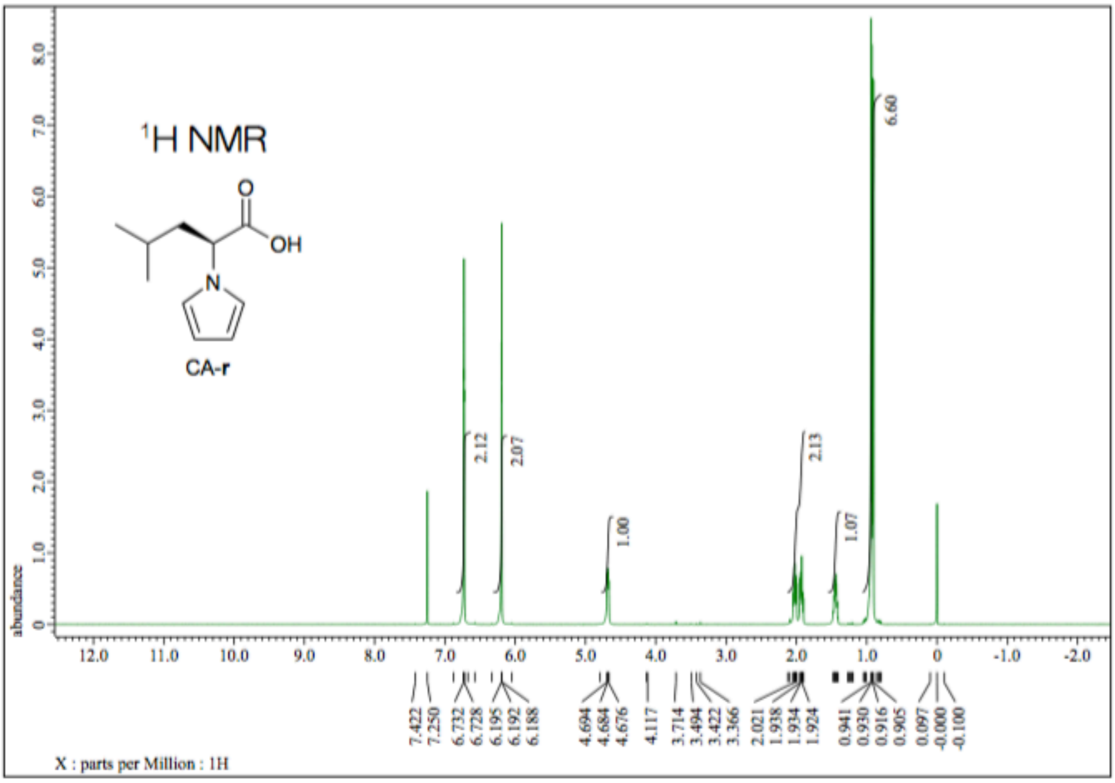

1H NMR
CA-r
X : parts per Million : 1H

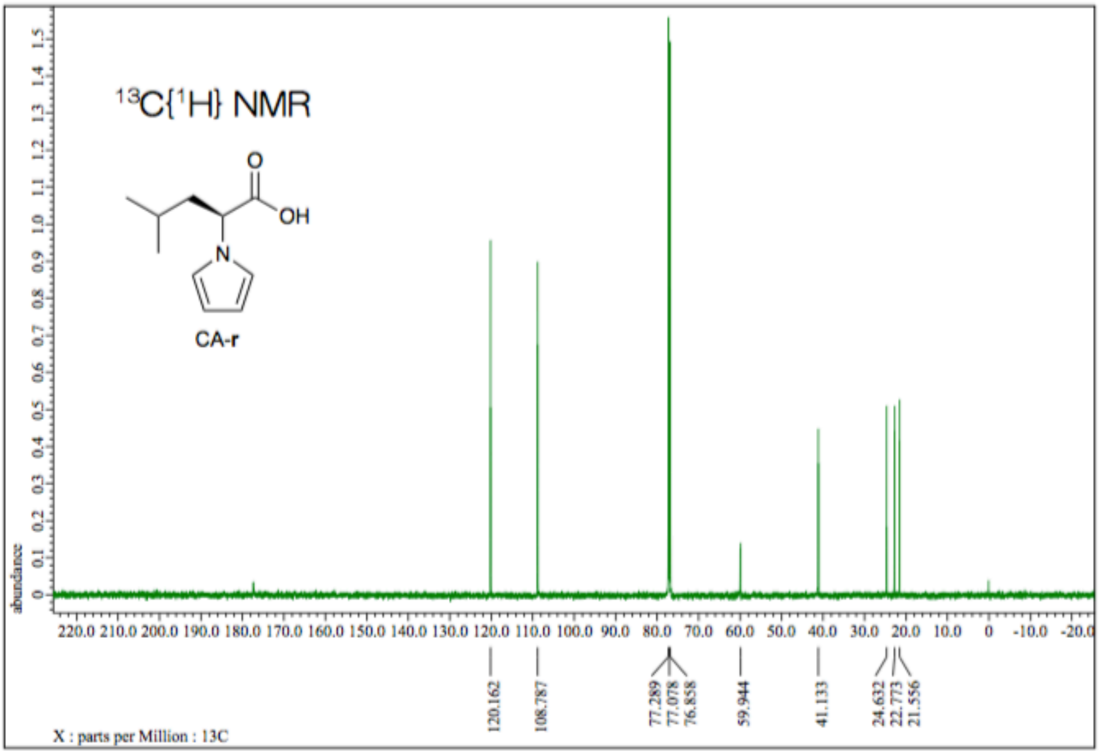

13C{1H} NMR
CA-r
X : parts per Million : 13C

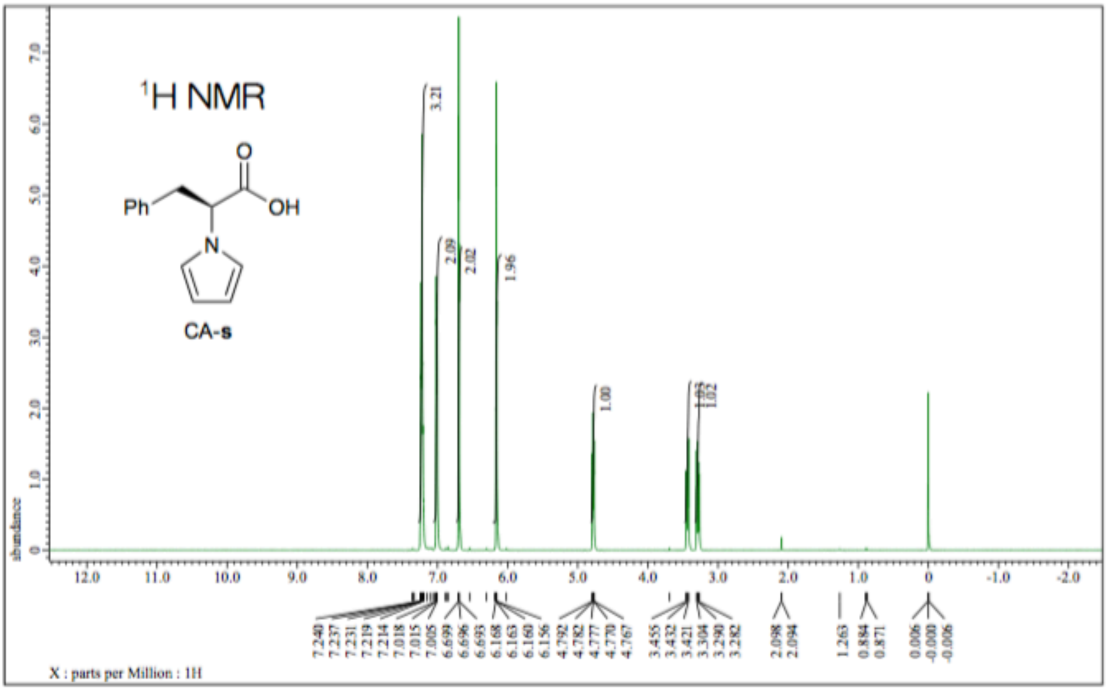
1H NMR
Ph
O
OH
N
CA-s
3.21
2.09
2.02
1.96
1.00
1.03
1.02
abundance
X : parts per Million : 1H
7.240 7.237 7.231 7.219 7.214 7.018 7.015 7.005 6.699 6.696 6.693 6.168 6.163 6.160 6.156 4.792 4.782 4.777 4.770 4.767 3.455 3.432 3.421 3.304 3.290 3.282 2.098 2.094 1.263 0.884 0.871 0.006 -0.000 -0.006

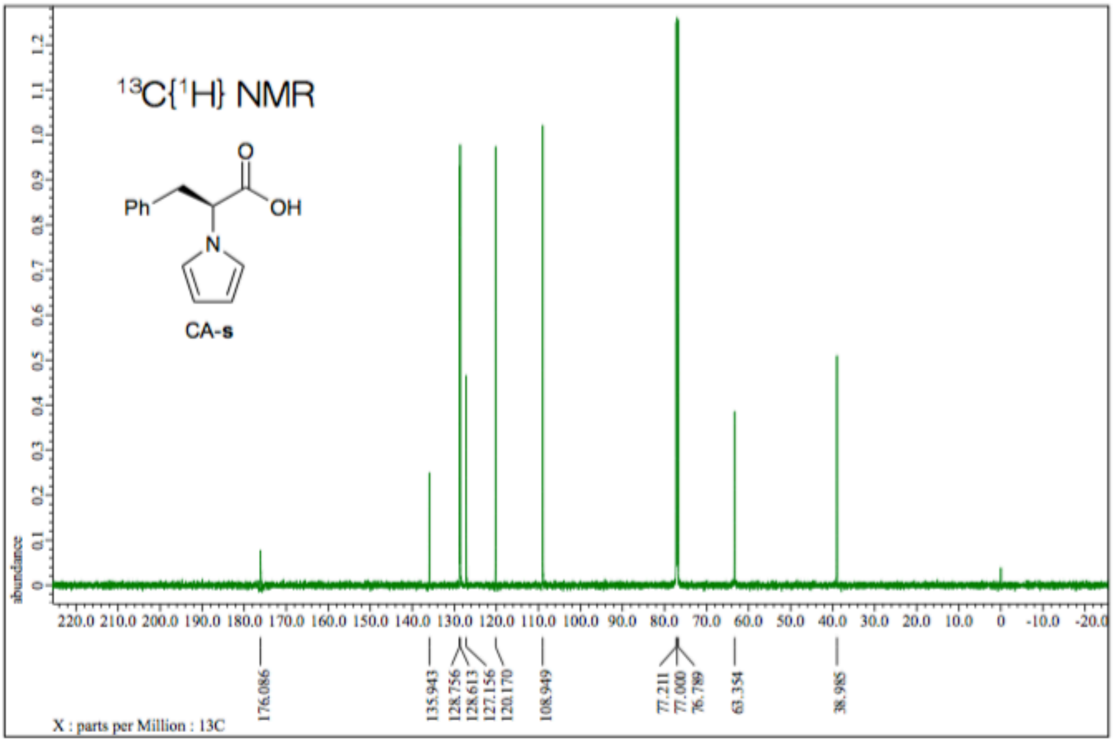
13C{1H} NMR
Ph
O
OH
N
CA-s
abundance
X : parts per Million : 13C
176.086 135.943 128.756 128.613 127.156 120.170 108.949 77.211 77.000 76.789 63.354 38.985

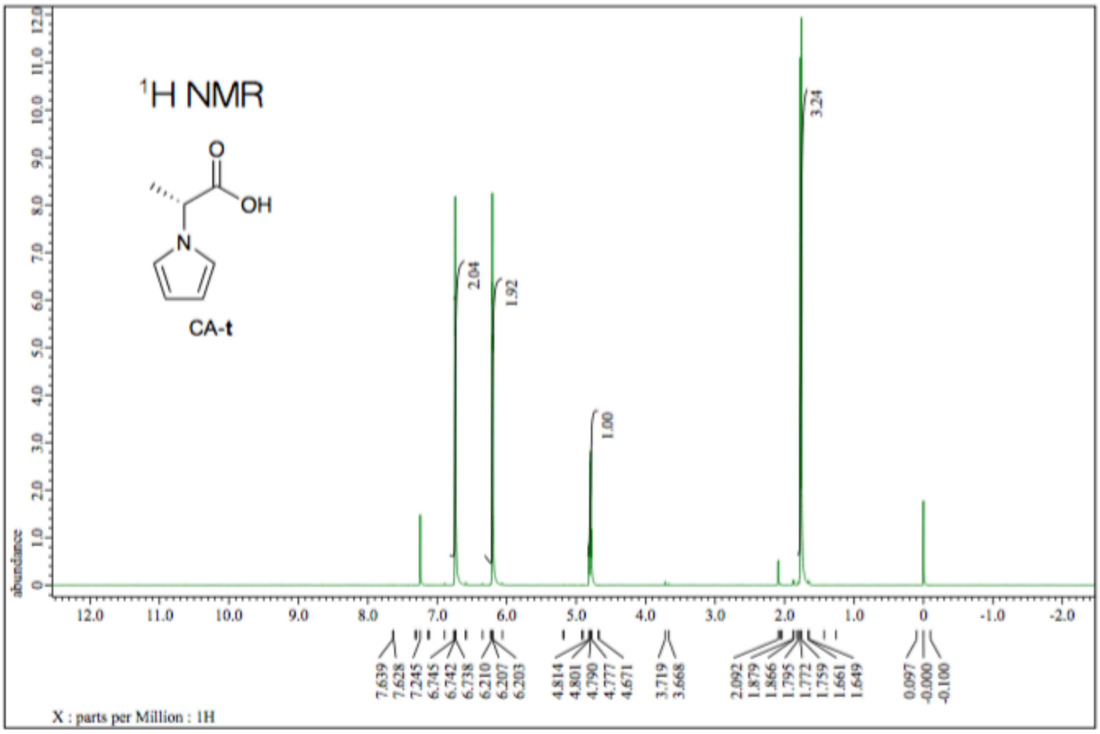

1H NMR
O
OH
N
CA-t
2.04
1.92
1.00
3.24
abundance
X : parts per Million : 1H

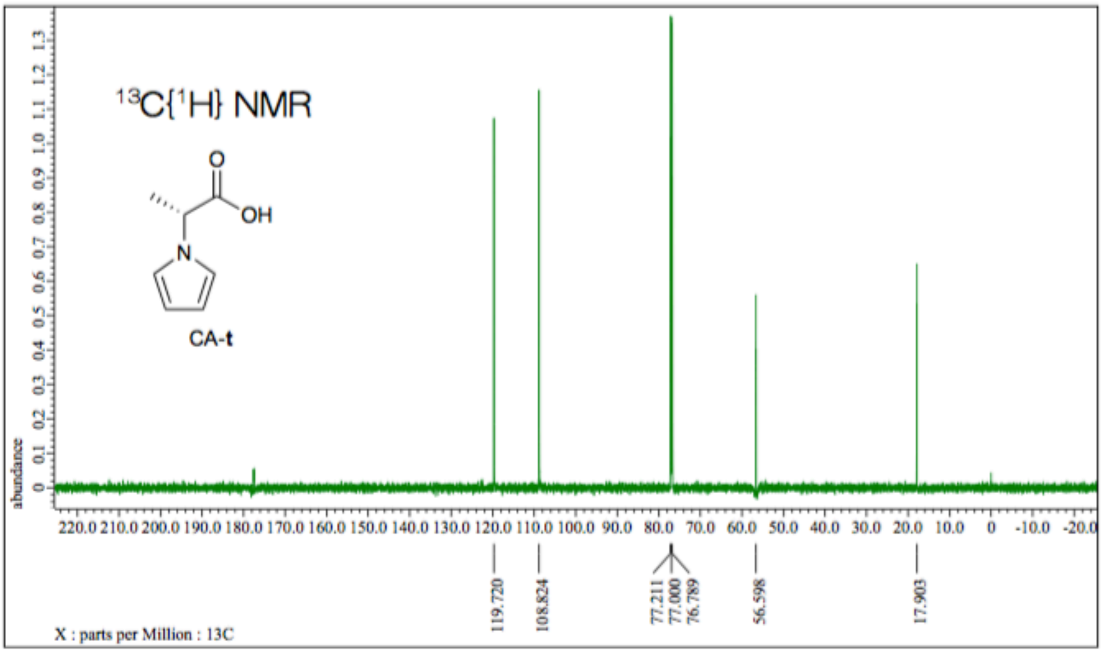

13C{1H} NMR
O
OH
N
CA-t
abundance
119.720
108.824
77.211
77.000
76.789
56.598
17.903
X : parts per Million : 13C

5.3. NMR spectra of new compounds obtained in hydrogenation and deuteration experiments

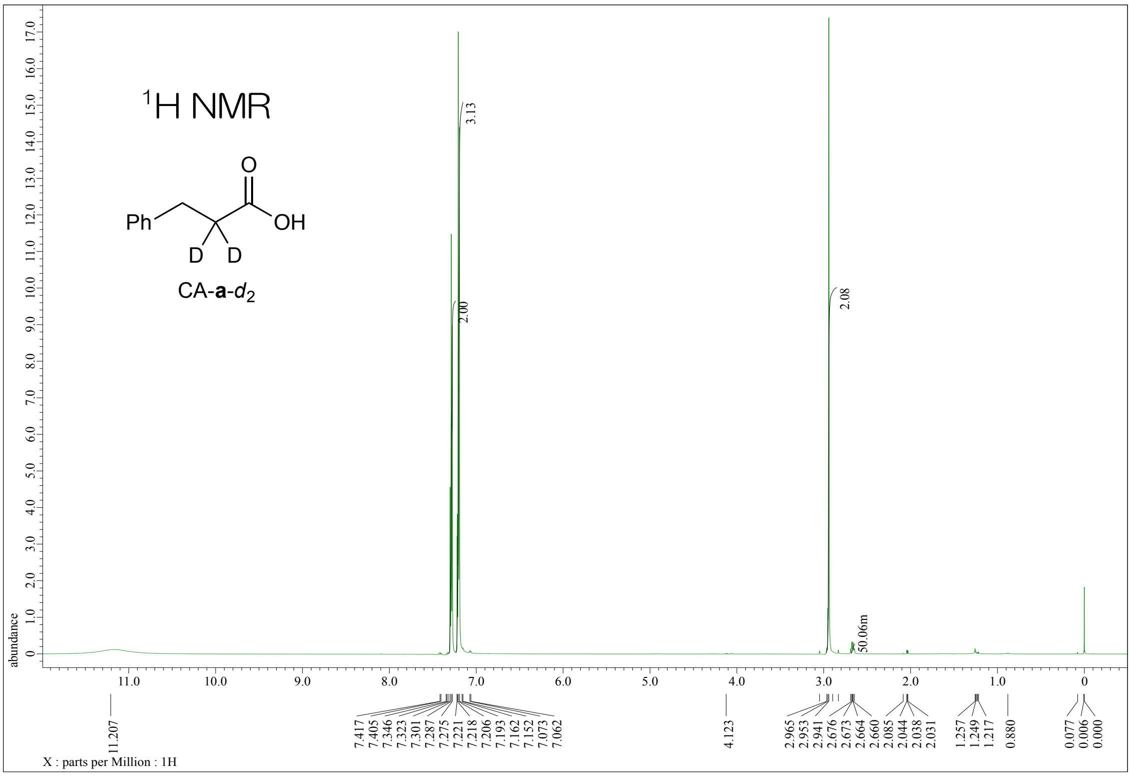

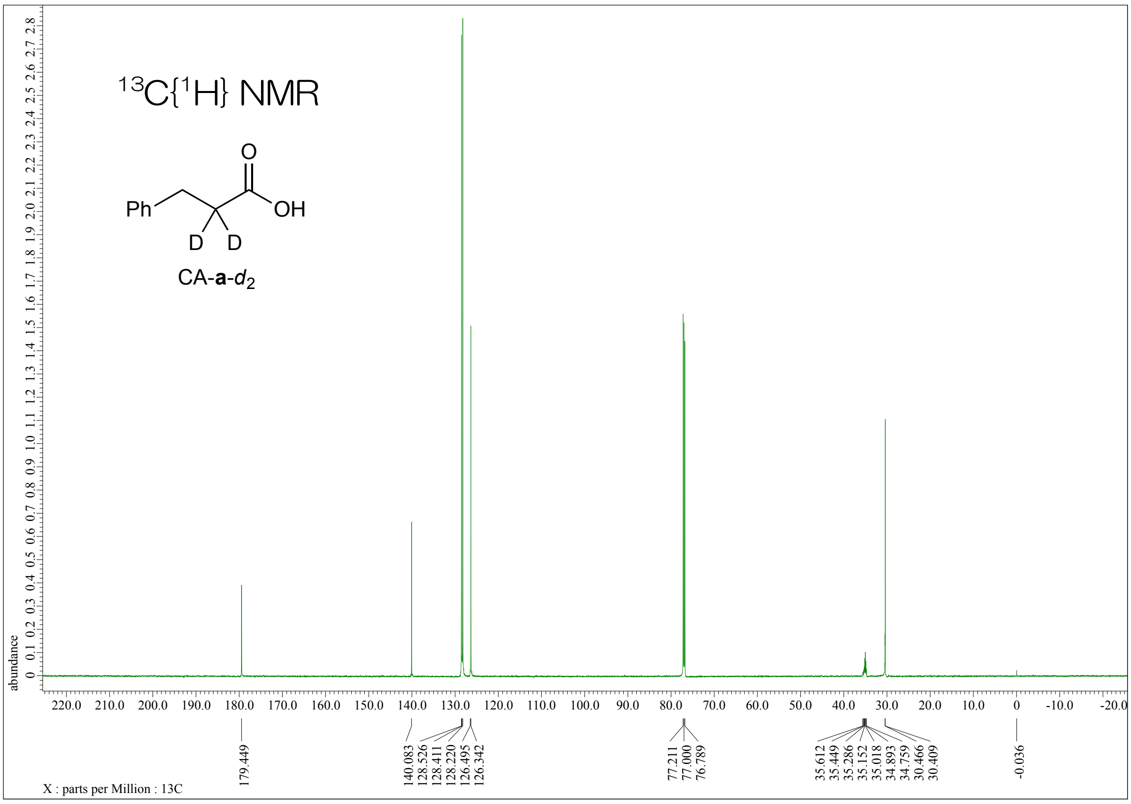

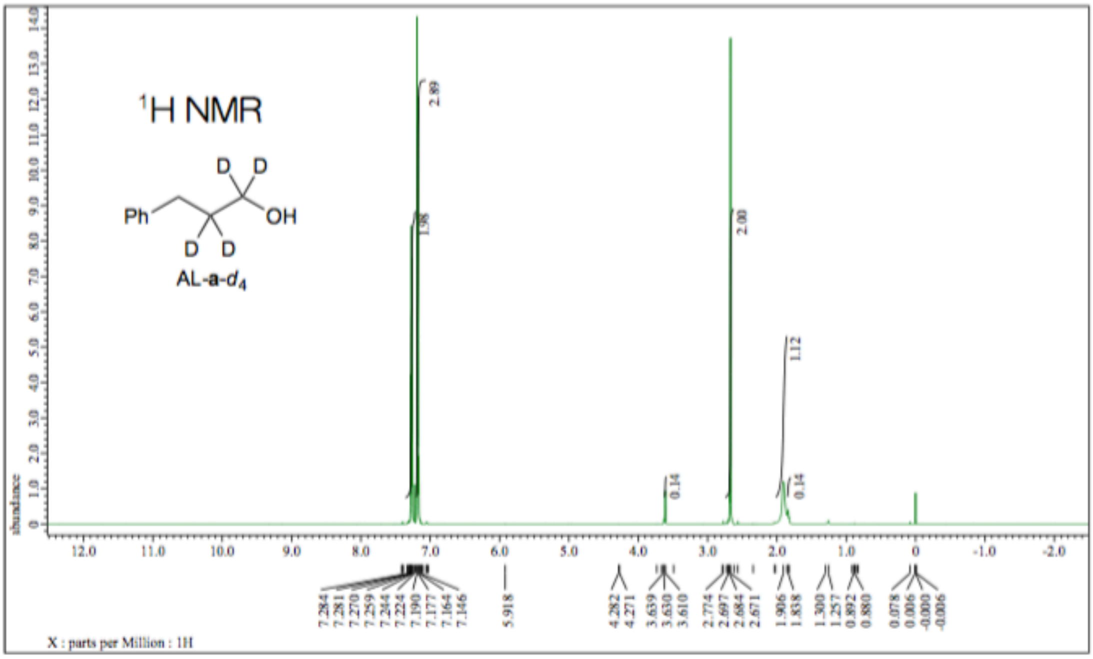

1H NMR
D D
Ph
OH
D D
AL-a-d4
abundance
X : parts per Million : 1H
2.89
1.98
2.00
1.12
0.14
0.14
7.284
7.281
7.270
7.259
7.244
7.224
7.190
7.177
7.164
7.146
5.918
4.282
4.271
3.639
3.630
3.610
2.774
2.697
2.684
2.671
1.906
1.838
1.300
1.257
0.892
0.880
0.078
0.006
-0.000
-0.006

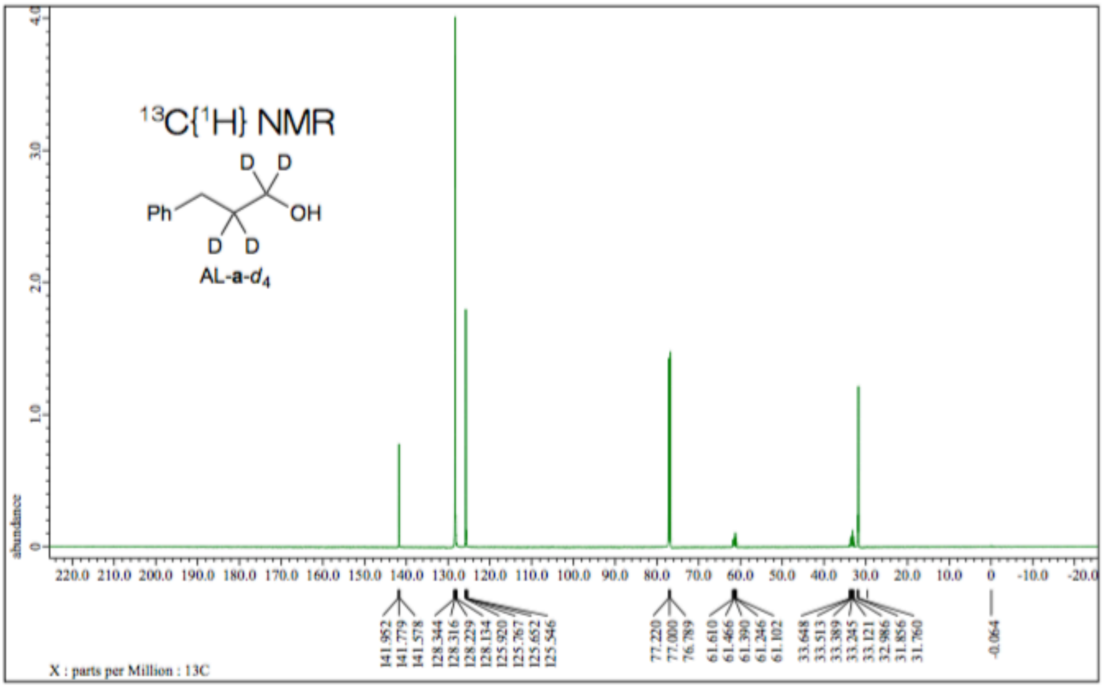

13C{1H} NMR
D D
Ph
OH
D D
AL-a-d4
abundance
X : parts per Million : 13C
141.952
141.779
141.578
128.344
128.316
128.229
128.134
125.920
125.767
125.652
125.546
77.220
77.000
76.789
61.610
61.466
61.390
61.246
61.102
33.648
33.513
33.389
33.245
33.121
32.986
31.856
31.760
-0.064

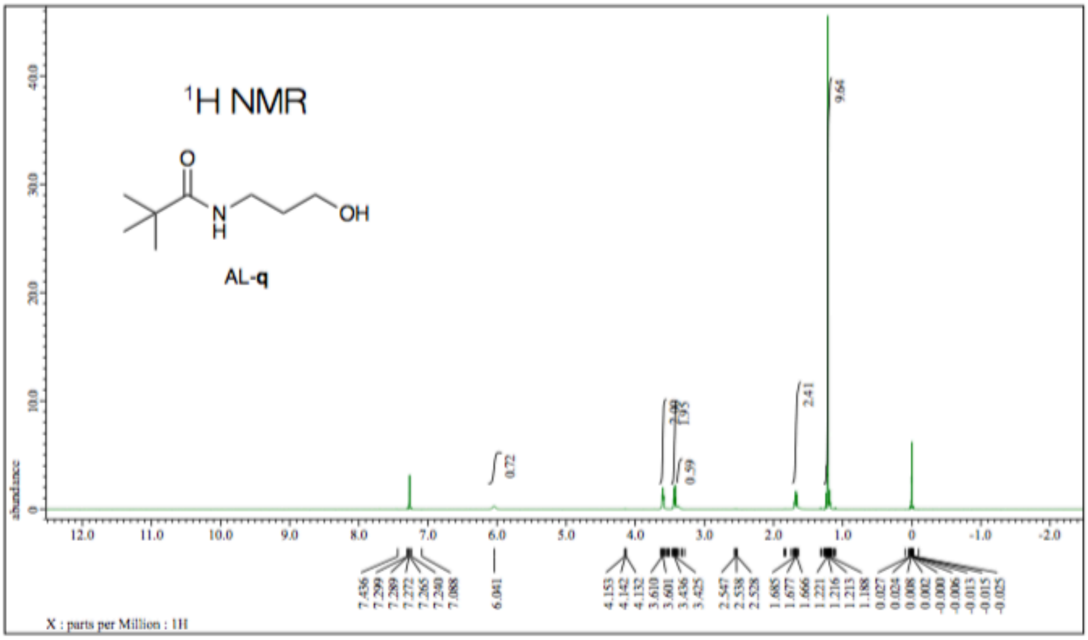

^{1}H NMR
AL-q
X : parts per Million : 1H

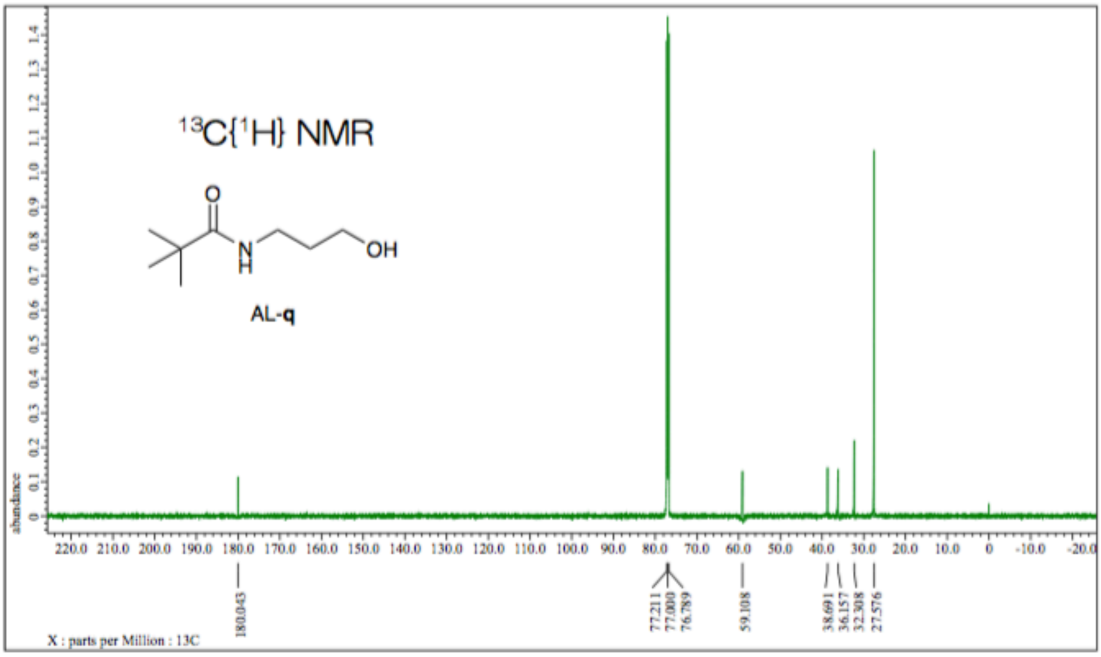

^{13}C{^{1}H} NMR
AL-q
X : parts per Million : 13C

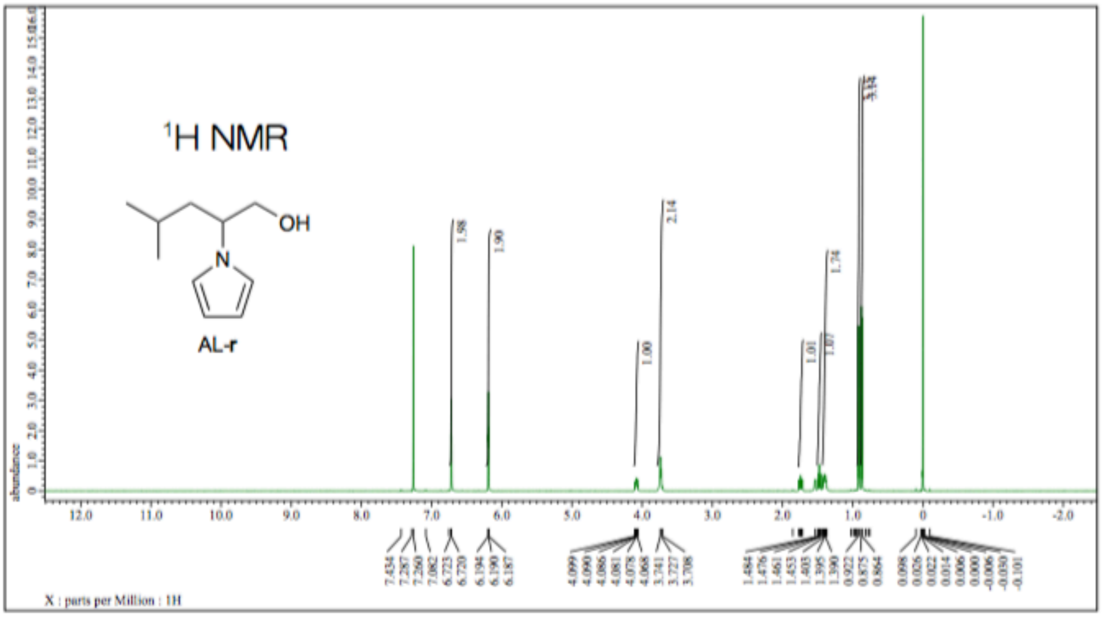

1H NMR
OH
N
AL-r
X : parts per Million : 1H

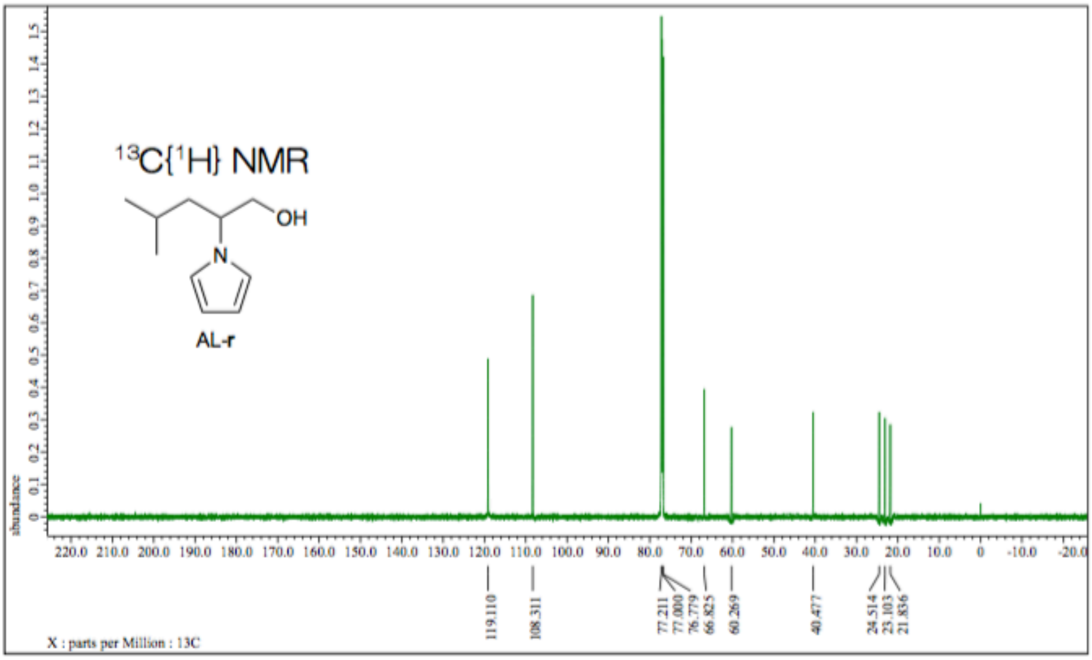

13C{1H} NMR
OH
N
AL-r
119.110
108.311
77.211
77.000
76.779
66.825
60.269
40.477
24.514
23.103
21.836
X : parts per Million : 13C

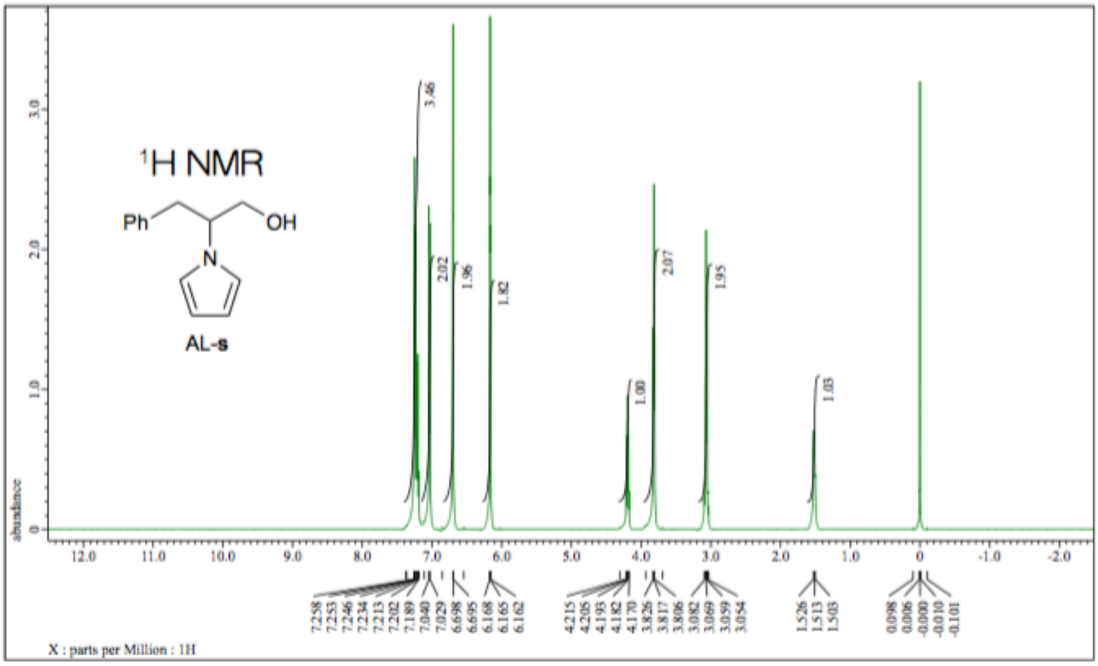

1H NMR
Ph
OH
N
AL-s
X : parts per Million : 1H
abundance

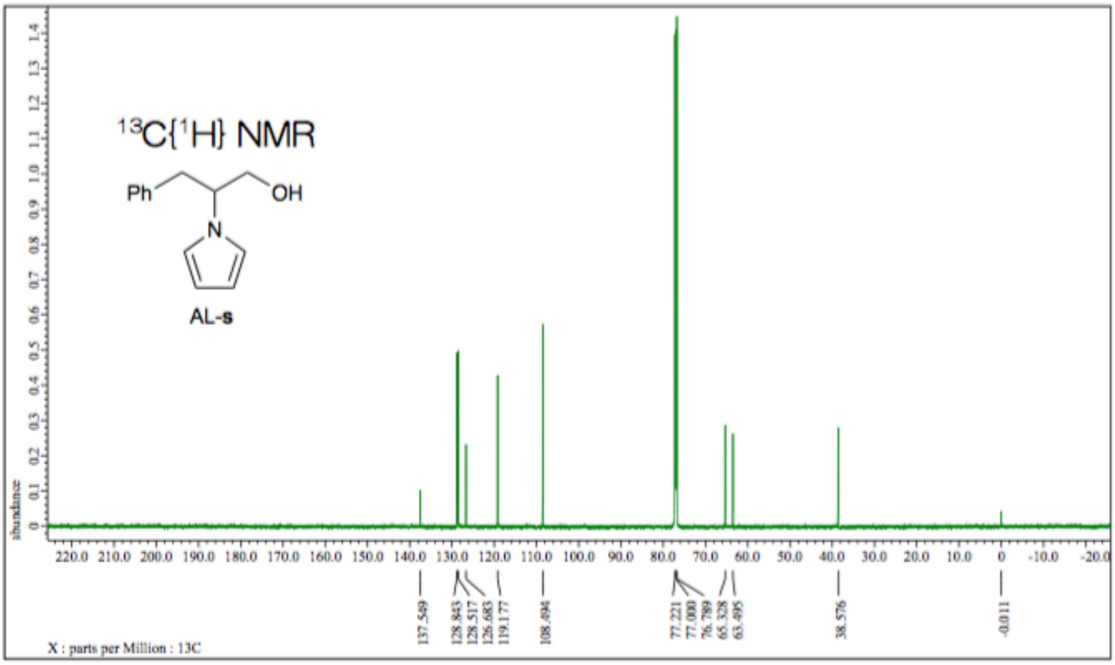

13C{1H} NMR
Ph
OH
N
AL-s
X : parts per Million : 13C
abundance

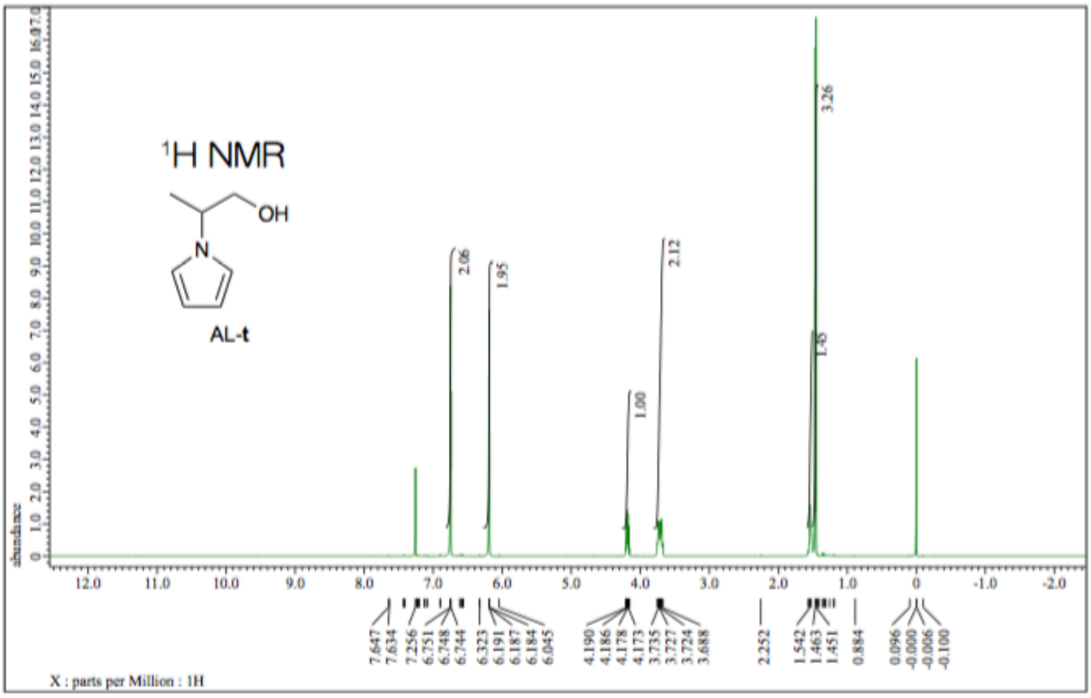
1H NMR
OH
N
AL-t
X : parts per Million : 1H

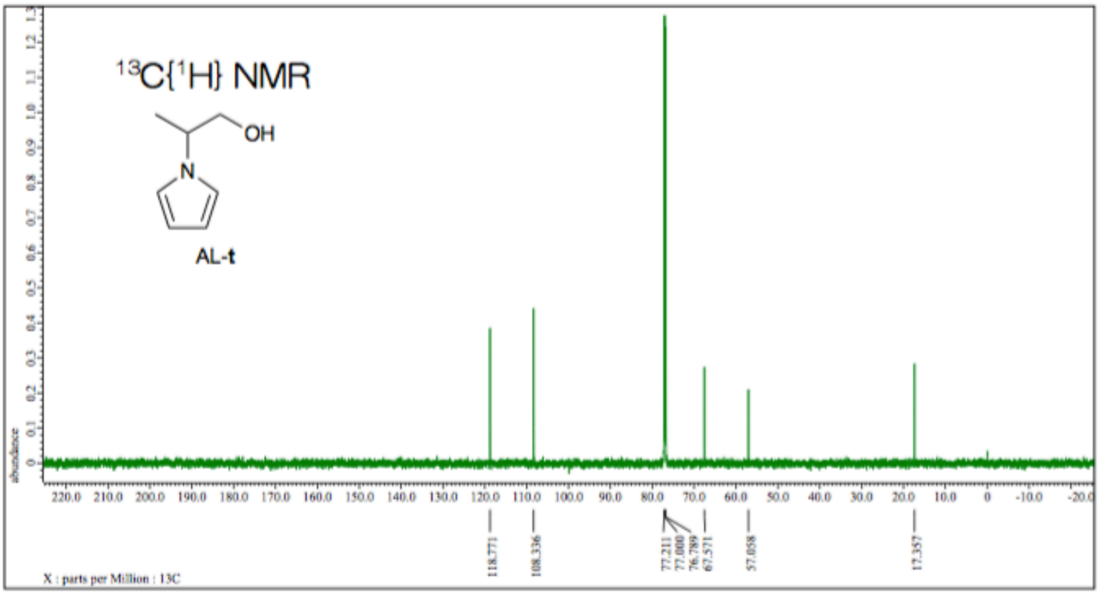
13C{1H} NMR
OH
N
AL-t
X : parts per Million : 13C

6. References

1. Sherry, B. D., Loy, R. N. & Toste, F. D. Rhenium(V)-catalyzed synthesis of 2-deoxy-α-glycosides. *J. Am. Chem. Soc.* **126,** 4510–4511 (2004).
2. Murphy, J. A., Schoenebeck, F., Findlay, N. J., Thomson, D. W., Zhou, S.-Z. & Garnier, J. One-carbon extrusion from a tetraazafulvalene. Isolation of aldehydes and a study of their origin. *J. Am. Chem. Soc*. **131,** 6475–6479 (2009).
3. Sakai, N., Kawana, K., Ikeda, R., Nakaike, Y. & Konakahara, T. $InBr_3$-Catalyzed Deoxygenation of Carboxylic Acids with a Hydrosilane: Reductive Conversion of Aliphatic or Aromatic Carboxylic Acids to Primary Alcohols or Diphenylmethanes *Eur. J. Org. Chem.* 3178–3183 (2011).
4. Kelly, C. B., Mercadante, M. A., Wiles, R. J. and Leadbeater, N. E. Oxidative Esterification of Aldehydes Using a Recyclable Oxoammonium Salt. *Org. Lett.* **15,** 2222–2225 (2013).
5. Yasuda, M., Onishi, Y., Ueba, M., Miyai, T. & Baba, A. Direct Reduction of Alcohols: Highly Chemoselective Reducing System for Secondary or Tertiary Alcohols Using Chlorodiphenylsilane with a Catalytic Amount of Indium Trichloride *J. Org. Chem.* **66,** 7741–7744 (2001).
6. Bodnar, B. S. & Vogt, P. F. An Improved Bouveault−Blanc Ester Reduction with Stabilized Alkali Metals. *J. Org. Chem.* **74,** 2598–2600 (2009).
7. Mangaleswaran, S. & Argade, N. P. A Facile Synthesis of Naturally Occurring Aminopeptidase Inhibitor Tyromycin A. *J. Org. Chem*. **66,** 5259–5261 (2001).
8. David, S. & Thieffry, A. Conversion of one hydroxy group in a diol to a phenyl ether with triphenylbismuth diacetate, a new glycol reaction showing strong axial preference in six-membered rings. *J. Org. Chem.* **48,** 441–447 (1983).
9. Gómez, C., Maciá, B., Lillo, V. J. and Yus, M. [1,2]-Wittig rearrangement from chloromethyl ethers. *Tetrahedron* **62,** 9832–9839 (2006).
10. Joncour, A., Décor, A., Thoret, A., Chiaroni, A. & Baudoin, O. Biaryl Axis as a Stereochemical Relay for the Enantioselective Synthesis of Antimicrotubule Agents *Angrew. Chem. Int. Ed.* **45,** 4149–4152 (2006).
11. Morales-Serna, J. A., García-Ríos, E., Bernal, J., Paleo, E., Gaviño, R. & Cárdenas, J. Reduction of Carboxylic Acids Using Esters of Benzotriazole as High-Reactivity Intermediates. *Synthesis* **9,** 1375–1382 (2011).

12. Taylor, E. C., & Gillespie, P. Further acyclic analogs of 5,10-dideaza-5,6,7,8-tetrahydrofolic acid. *J. Org. Chem.* **57,** 5757–5761 (1992).
13. Mamolo, M. G., Zampieri, D., Zanette, C., Florio, C., Collina, S., Urbano, M., Azzolina, O. & Vio, L. Substituted benzylaminoalkylindoles with preference for the σ_2 binding site. *Eur. J. Med. Chem.* **43,** 2073–2081 (2008).
14. Bach, A., Pizzirani, D., Realini, N., Vozella, V., Russo, D., Penna, I., Melzig, L., Scarpelli, R. & Piomelli, D. Benzoxazolone Carboxamides as Potent Acid Ceramidase Inhibitors: Synthesis and Structure−Activity Relationship (SAR) Studies. *J. Med. Chem.* **58,** 9258–9272 (2015).
15. Shaikh, N. S., Junge, K. & Beller, M. A Convenient and General Iron-Catalyzed Hydrosilylation of Aldehydes. *Org. Lett.* **9,** 5429–5432 (2007).
16. Dieskau, A. P., Begouin, J.-M. & Plietker, B. $Bu_4N[Fe(CO)_3(NO)]$-Catalyzed Hydrosilylation of Aldehydes and Ketones. *Eur. J. Org. Chem.* 5291–5296 (2011).
17. Egami, H. & Katsuki, T. Optimization of Asymmetric Oxidation of Sulfides with the Fe(salan) Complex in Water and the Expanded Scope of its Application. *Synlett.* **10,** 1543–1546 (2008).
18. Wypych, J.-C., Nguyen, T. M., Bénéchie, M. & Marazano, C. Reaction of Aldimine Anions with Vinamidinium Chloride: Three-Component Access to 3-Alkylpyridines and 3-Alkylpyridinium Salts and Access to 2-Alkyl Glutaconaldehyde Derivatives. *J. Org. Chem.* **73,** 1169–1172 (2008).
19. Yamamoto, N., Obora, Y. & Ishii, Y. Iridium-Catalyzed Oxidative Methyl Esterification of Primary Alcohols and Diols with Methanol. *J. Org. Chem.* **76,** 2937–2941 (2011).
20. Ghosh, S. C., Ngiam, J. S. Y., Seayad, A. M. & Tuan, D. T. Copper-Catalyzed Oxidative Amidation of Aldehydes with Amine Salts: Synthesis of Primary, Secondary, and Tertiary Amides. *J. Org. Chem.* **77,** 8007–8015 (2012).
21. Ambre, R., Yu, C.-Y., Mane, S. B., Yao, C.-F. & Hung, C.-H. Toward carboxylate group functionalized A_4, A_2B_2, A_3B oxaporphyrins and zinc complex of oxaporphyrins. *Tetrahedron* **67,** 4680–4688 (2011).
22. Taylor, D. C., Wightman, R. H., Wightman, F. & Wand, A. Synthesis of Di- and trichlorophenylalanines. *Bioorg. Chem.* **15,** 335–445 (1987).

23. Murai, N., Yonaga, M. & Tanaka, K. Palladium-Catalyzed Direct Hydroxymethylation of Aryl Halides and Triflates with Potassium Acetoxymethyltrifluoroborate. *Org. Lett.* **14,** 1278–1281 (2012).
24. Gabriele, B., Mancuso, R. & Salerno, G. A Novel Synthesis of 2-Functionalized Benzofurans by Palladium-Catalyzed Cycloisomerization of 2-(1-Hydroxyprop-2-ynyl)phenols Followed by Acid-Catalyzed Allylic Isomerization or Allylic Nucleophilic Substitution. *J. Org. Chem*. **73,** 7336–7341 (2008).
25. Ramadas, S. & Krupadanam, G. L. D. Enantioselective acylation of 2-hydroxymethyl-2,3-dihydrobenzofurans catalysed by lipase from *Pseudomonas cepacia* (Amano PS) and total stereoselective synthesis of (−)-(*R*)-MEM-protected arthrographol. *Tetrahedron: Asymmetry* **11,** 3375–3393 (2000).
26. An, J., Work, D. N., Kenyon. C., & Procter, D. J. Evaluating a Sodium Dispersion Reagent for the Bouveault–Blanc Reduction of Esters. *J. Org. Chem.* **79,** 6743–6747 (2014).
27. Gibson, S. P. & Lauret, C. Preparation of benzofuranyl antiparasitic agents for use in nonhum animals. US20050054630 A (2005).
28. Liu, Y., Yao, B., Deng, C.-L., Tang, R.-Y., Zhang, X.-G. & Li, J.-H. Palladium-Catalyzed Selective Heck-Type Diarylation of Allylic Esters with Aryl Halides Involving a β-OAc Elimination Process. *Org. Lett.* **13,** 1126–1129 (2011).
29. Jiménez, T., Barea, E., Oltra, J. E., Cuerva, J. M. & Justicia, J. Mn(0)-Mediated Chemoselective Reduction of Aldehydes. Application to the Synthesis of α-Deuterioalcohols. *J. Org. Chem.* **75,** 7022–7025 (2010).
30. Larionov, E., Lin, L., Guénée, L. & Mazet, C. Scope and Mechanism in Palladium-Catalyzed Isomerizations of Highly Substituted Allylic, Homoallylic, and Alkenyl Alcohols. *J. Am. Chem. Soc.* **136,** 16882–16894 (2014).
31. Li, Y., Xue, D., Lu, W., Wang, C., Liu, Z.-T. & Xiao, J. DMF as Carbon Source: Rh-Catalyzed α-Methylation of Ketones. *Org. Lett.* **16,** 66–69 (2014).
32. Zheng, H.-X., Xiao, Z.-F., Yao, C.-Z., Li, Q.-Q., Ning, X.-S., Kang, Y.-B., & Tang, Y. Transition-Metal-Free Self-Hydrogen-Transferring Allylic Isomerization. *Org. Lett.* **17,** 6102–6105 (2015).
33. K. -V. Tran & Bickar, D. Dalkin–West Synthesis of β-Aryl Ketones. *J. Org. Chem.* **71,** 6640–6643 (2006).

34. B.-Robert, F. & Beauchamp, A. L. Preparation and electronic properties of rhenium(V) complexes with bis(diphenylphosphino)ethane. *Can. J. Chem.* **81,** 1326–1340 (2003).
35. Luo, X,-L. & Crabtree, R. C. Synthesis and Structural Studies of Some New Rhenium Phosphine Heptahydride Complexes. Evidence for Classical Structures in Solution. *J. Am. Chem. Soc.* **112,** 4813–4821 (1990).
36. Parr, M. L., Parez-Acosta, C. & Faller, J. W. Synthesis, characterization and structural investigation of new rhenium-oxo complexes containing bidentate phosphine ligands: an exploration of chirality and conformation in chelate rings of small and large bite angle ligands. *New J. Chem.* **29,** 613–619 (2005).
37. Bianchini, C., Peruzzini, M. & Zanobini, F. Synthesis and characterization of rhenium polyhydrides stabilized by the tripodal ligand $MeC(CH_2PPh_2)_3$. *J. Organomet. Chem.* **451,** 97–106 (1993).
38. Pechlivanidis, Z., Hopf, H. & Ernst, L. Paracyclophanes: extending the bridges. Synthesis. *Eur. J. Org. Chem.* 223–237 (2009).
39. Yamamoto, T., Niwa, S., Iwayama, S., Kogenei, H., Fujita, S., Takeda, T, Kito, M., Ono, Y., Saitou, Y., Takahara, A., Iwata, S., Yamamoto, H. & Shoji, H. Discovery, structure–activity relationship study, and oral analgesic efficacy of cyproheptadine derivatives possessing N-type calcium channel inhibitory activity. *Bioorgan. Med. Chem.* **14,** 5333–5339 (2006).
40. Jefford, C. W., de Villedon de Naide, F., Sienkiewicz, K. The synthesis of chiral 1-(1*H*-pyrrole) derivatives. *Tetrahedron: Asymmetry* **7,** 1069–1076 (1996).
